# Screening of candidate regulators for cellulase and hemicellulase production in *Trichoderma reesei* and identification of a factor essential for cellulase production

**DOI:** 10.1186/1754-6834-7-14

**Published:** 2014-01-28

**Authors:** Mari Häkkinen, Mari J Valkonen, Ann Westerholm-Parvinen, Nina Aro, Mikko Arvas, Marika Vitikainen, Merja Penttilä, Markku Saloheimo, Tiina M Pakula

**Affiliations:** 1 VTT Technical Research Centre of Finland, PO Box 1000 Tietotie 2, Espoo FI-02044, VTT, Finland

**Keywords:** Carbohydrate active enzymes, Cellulase, Co-regulation, Gene regulation, Hemicellulase, Transcription factors, Transcriptional profiling

## Abstract

**Background:**

The soft rot *ascomycetal* fungus *Trichoderma reesei* is utilized for industrial production of secreted enzymes, especially lignocellulose degrading enzymes. *T. reesei* uses several different enzymes for the degradation of plant cell wall-derived material, including 9 characterized cellulases, 15 characterized hemicellulases and at least 42 genes predicted to encode cellulolytic or hemicellulolytic activities. Production of cellulases and hemicellulases is modulated by environmental and physiological conditions. Several regulators affecting the expression of cellulase and hemicellulase genes have been identified but more factors still unknown are believed to be present in the genome of *T. reesei*.

**Results:**

We have used transcriptional profiling data from *T. reesei* cultures in which cellulase/hemicellulase production was induced by the addition of different lignocellulose-derived materials to identify putative novel regulators for cellulase and hemicellulase genes. Based on this induction data, supplemented with other published genome-wide data on different protein production conditions, 28 candidate regulatory genes were selected for further studies and they were overexpressed in *T. reesei*. Overexpression of seven genes led to at least 1.5-fold increased production of cellulase and/or xylanase activity in the modified strains as compared to the parental strain. Deletion of gene 77513, here designated as *ace3*, was found to be detrimental for cellulase production and for the expression of several cellulase genes studied. This deletion also significantly reduced xylanase activity and expression of xylan-degrading enzyme genes. Furthermore, our data revealed the presence of co-regulated chromosomal regions containing carbohydrate-active enzyme genes and candidate regulatory genes.

**Conclusions:**

Transcriptional profiling results from glycoside hydrolase induction experiments combined with a previous study of specific protein production conditions was shown to be an effective method for finding novel candidate regulatory genes affecting the production of cellulases and hemicellulases. Recombinant strains with improved cellulase and/or xylanase production properties were constructed, and a gene essential for cellulase gene expression was found. In addition, more evidence was gained on the chromatin level regional regulation of carbohydrate-active enzyme gene expression.

## Background

Plant biomass, consisting mostly of cellulose, hemicellulose and lignin, is the most abundant renewable energy source on earth. Degradation of the biomass and continuation of the carbon cycle is maintained mainly by microbial action, especially by fungi of different species. The biomass-degrading enzymes produced by these organisms also have applications in different fields of industry, including biorefinery applications [1]. *Trichoderma reesei* (an anamorph of *Hypocrea jecorina*) is an extremely efficient producer of cellulose- and hemicellulose-degrading enzymes, and is therefore widely employed by the enzyme industry for the production of its own enzymes as well as for producing proteins from other sources [2,3]. The genome of *T. reesei* encodes nine characterized cellulase enzymes and 15 characterized hemicellulase enzymes. In addition, a large number of genes encoding candidate carbohydrate-active enzymes (CAZy) [4,5] have been identified from the genome [6,7]. According to an updated annotation, the genome encodes 201 glycoside hydrolase genes, 22 carbohydrate esterase genes and 5 polysaccharide lyase genes, of which at least 66 are known or predicted to encode cellulolytic and hemicellulolytic activities [8].

Energy efficient production of cellulases and hemicellulases is achieved by tight gene regulation governed by inducer-dependent expression of the genes and by repression of the genes in the presence of fast metabolized carbon sources (for reviews see [9,10]). In addition to the type of carbon source, additional environmental conditions are known to affect protein production together with the physiological state of the cells, such as pH [11], light [12], the specific growth rate and cell density of the fungus [13,14], and the physiological state of the mitochondria [15]. Furthermore, the expression of many cellulase and hemicellulase genes is shown to be under a feedback regulation mechanism that functions under conditions in which the capacity of the cells to fold and secrete proteins is limited and transcriptional down-regulation is required to reduce the amount of secreted protein produced [16].

The variety of environmental and physiological factors affecting the enzyme production of *T. reesei* infers that a complex signaling cascade and regulatory network is needed for the accurate timing of hydrolytic enzyme production. Several regulatory factors for cellulase and hemicellulase genes have been characterized, the most extensively studied of which are the transcription factor CRE1, which mediates carbon catabolite repression [17], and the major regulator needed for expression, XYR1 [18]. Other characterized factors are the positively acting ACE2 [19] and HAP2/3/5 complex [20], and the negatively acting factor ACE1 [21,22]. Recently, novel factors possibly affecting the regulation of genes encoding hydrolytic enzymes have been found from *Trichoderma* and other fungi. F-box proteins that have been suggested to be involved in the regulation of plant cell wall-degrading enzymes have been identified from *Aspergillus* and *Fusarium*[23,24]. Two putative regulators of cellulase and hemicellulase genes named CLR-1 and CLR-2 have been identified from *Neurospora crassa*[25] and a transcription factor BglR has been suggested to regulate β-glucosidase genes of *T. reesei*[26]. Another recent finding is that the putative methyltransferase LAE1 is essential for the formation of *T. reesei* cellulases and hemicellulases, although the precise mechanism is still unclear [27]. In the light of recent findings from *Trichoderma* and other fungi, it can be assumed that not all regulatory factors have been identified yet and that additional regulatory genes can still be found in the genome of *T. reesei*.

In this study, transcriptional profiling data from *T. reesei* cultivated in the presence of several lignocellulose substrates as well as other genome-wide data from different types of protein production conditions were used to identify putative regulators for cellulase and hemicellulase genes. Several candidate regulatory genes were identified, and shown to have an effect on cellulase and hemicellulase production when overexpressed in *T. reesei*. Furthermore, the genomic context of the CAZy genes and co-regulated candidate regulatory genes were analyzed. The data revealed co-regulated regions containing candidate regulatory genes and CAZy genes, as well as other genes relevant for the utilization of the carbon source, such as transporter genes. The relevance of the regions is discussed in the paper.

## Results

### Analysis of transcriptome data to identify candidates for regulators of cellulase and hemicellulase genes

**Figure 1:**
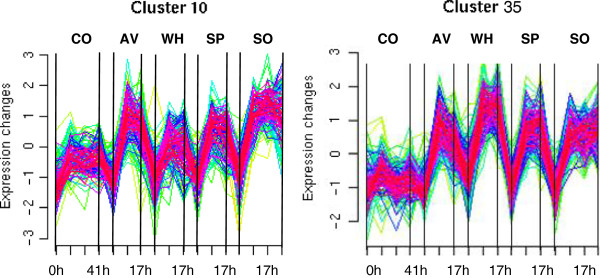
**Expression profiles of the clusters from Mfuzz clustering containing the majority of the cellulase and hemicellulase genes.** The expression array dataset on *T. reesei* cultures induced with Avicel cellulose, pretreated wheat, pretreated spruce or sophorose (described in [8]) were clustered using Mfuzz. AV, Avicel cellulose; CO, control cultivation; SO, sophorose; SP, spruce; WH, wheat straw.

Transcriptome analysis has previously been carried out to study the expression of CAZy genes in *T. reesei* cultures that were induced by the addition of different types of lignocellulose material, purified carbohydrate polymers or disaccharides (Avicel cellulose, pretreated wheat straw, pretreated spruce or sophorose) [8]. In the present study, data from the previous work were further analyzed and explored to identify candidate regulators for CAZy genes and, in particular, for cellulase and hemicellulase genes. The expression data were clustered using Mfuzz [28,29] to reveal groups of co-regulated genes. The majority of the genes encoding characterized enzymes and accessory factors involved in lignocellulose degradation were found in two clusters. Cluster 10 contained the major cellulase and β-glucosidase genes (*cbh1, cbh2, egl1, egl2, egl3, egl5, bgl1* and *bgl2*) together with a set of hemicellulase genes (*abf1, bga1, cip2, cel74a* and *xyn3*). Cluster 35 contained predominantly hemicellulase genes (*agl1, agl3, man1, aes1, axe1, bxl1, glr1, xyn1 and xyn4*) (Figure 1; for gene names, see [8]). Only a few characterized hemicellulase genes were found outside these clusters (α-galactosidase genes 1 and 2, and xylanase 2 gene). A large number of putative regulatory genes clustered together with the known cellulase and hemicellulase genes. In particular, many genes encoding putative fungal C6 zinc finger-type transcription factors (containing InterPro domains IPR001138 fungal transcriptional regulatory protein, N-terminal and/or IPR007219 transcription factor, fungi [30]) were enriched within the clusters (*P* = 0.00027). Within the clusters, 5.9% of the genes encoded the predicted fungal type transcription factors, whereas only 2.5% of the total genome content belonged to this class. In addition, the clusters contained genes encoding candidates for other types of Zinc finger proteins, kinases and proteins involved in chromatin remodeling or organization, as well as proteins with InterPro domains indicating different regulatory or signal transduction functions (for the classes of the genes, see Table 1). A few known regulators were among the co-expressed genes, such as *xyr1,* the major regulator for cellulase and hemicellulase expression [18], and the homologues for *N. crassa clr-2*[25], *Aspergillus nidulans creC*[31] and *Fusarium oxysporum frp1*[24].

**Table 1:** Classes, functional domains and domain descriptions of the candidate regulators encoded by the genes that are co-regulated with cellulase and/or hemicellulase genes

**Class**	**InterPro domain**	**Domain description**	**Candidate regulatory genes**
Fungal transcription factors (Zn2-C6 type)	IPR001138	Fungal transcriptional regulatory protein, N-terminal	31
	IPR007219	Transcription factor, fungi	
Transcription factors, basic-leucine zipper type	IPR004827	Basic-leucine zipper transcription factor	1
	IPR011616	Basic-leucine zipper transcription factor, bZIP-1	
Transcription factor, Tcf25 type repressor	IPR006994	Transcription factor 25	1
Zinc finger, C2H2 type	IPR007087	Zinc finger, C2H2-type	2
Zinc finger, other types	IPR002893	Zinc finger, MYND-type	1
	IPR000058	Zinc finger, AN1-type	1
	IPR002867	Zinc finger, C6HC-type	1
	IPR008913	Zinc finger, CHY-type	1
	IPR000571	Zinc finger, CCCH-type	1
Chromatin level regulation/remodeling	IPR013256	Chromatin SPT2	1
	IPR000953	Chromo domain	1
	IPR008251	Chromo shadow	
	IPR000182	GCN5-related N-acetyltransferase	6
	IPR013178	Histone H3-K56 acetyltransferase, RTT109	1
	IPR001214	SET domain	2
	IPR000330	SNF2-related	1
	IPR001025	Bromo adjacent homology domain	1
	IPR001487	Bromodomain	1
	IPR000210	BTB/POZ-like	
Protein kinases	IPR000719	Protein kinase, catalytic domain	7
	IPR011009	Protein kinase-like domain	
G protein signaling	IPR011021	Arrestin-like, N-terminal	1
	IPR011022	Arrestin-like, C-terminal	
	IPR000832	G protein-coupled reseptor, family 2, secretin-like	1
	IPR000342	Regulator of G protein signaling	2
Other regulators	IPR000095	PAK-box/P21-Rho-binding	
	IPR000387	Dual-specific/protein-tyrosine phosphatase, conserved region	1
	IPR000791	GPR1/FUN34/yaaH	1
	IPR009057	Homeodomain-like	1
	IPR001611	Leucine-rich repeat	1
	IPR008030	NmrA-like	1
	IPR008914	Phosphatidylethanolamine-binding protein	1
	IPR012093	Pirin	2
	IPR011989	Armadillo-like helical	2
	IPR001313	Pumilio RNA-binding repeat	
	IPR001251	CRAL-TRIO domain	1
	IPR005511	Senescence marker protein-30	1
	IPR001810	F-box domain, cyclin-like	1
	IPR003892	Ubiquitin system component Cue	1
	IPR001680	WD40 repeat	4

To cover putative regulatory genes induced by the substrates but showing different temporal patterns and extent of induction (and therefore not clustered together with the characterized cellulase and hemicellulase genes), the differentially expressed genes at each of the time points were identified by comparing the expression level in the induced cultures to the level in the uninduced control cultures (using Limma package (R, Bioconductor) [28,32], and the cut-off *P* <0.01 in the statistical analysis). Altogether, 89 genes with putative regulatory functions were either co-clustered with the characterized cellulase and hemicellulase genes or showed increased signal level in most of the inducing conditions studied.

In order to get further support for the relevance of the 89 candidate genes in cellulase and hemicellulase production and to narrow down the number of genes to be selected for further studies, the expression of the candidate genes was compared in additional datasets on different protein production conditions. Transcriptome and proteome data from chemostat cultures with different specific growth rate, cell density and specific protein production rate [14] were explored for expression of the candidate genes and production of the corresponding proteins. Expression of 14 candidate genes showed either positive or negative correlation (absolute value <0.5) to the specific protein production rate in the chemostat cultures. Proteome analysis of the same cultures [14] showed that the candidate GCN5-related N-acetyltransferase (123668) was more abundant in the cultures with higher protein (and cellulase) production level, whereas the SEC14-domain protein 81972 and the candidate GCN5-related N-acetyltransferase (120120) were more abundant in the cultures with low protein (cellulase) production. The results are in accordance with the positive and negative correlation of the expression of genes 123668 and 81972 with the specific protein production rate, respectively. Gene 120120 showed a slightly negative correlation with the specific protein production rate.

**Figure 2:**
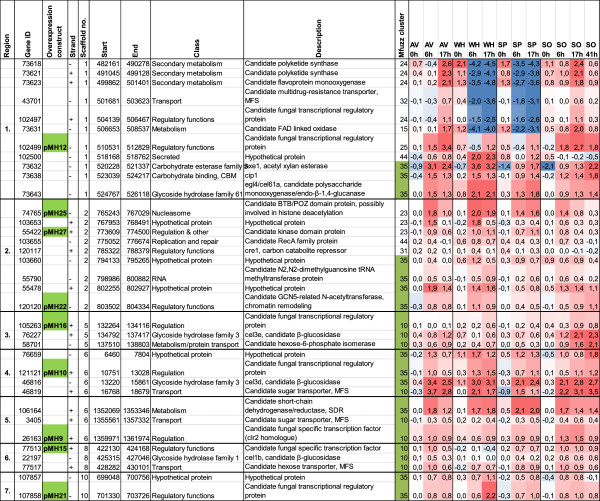
**Tightly co-expressed genomic regions with candidate regulatory genes.** The expression array dataset described in [8] was searched for genomic regions with co-expressed genes. The regions containing a selected candidate regulatory gene with adjacent genes belonging to the same Mfuzz gene expression clusters as the major cellulase and hemicellulase genes are shown. The genomic location of the genes is indicated as scaffold number, start and end position, and strand in the scaffold as in *T. reesei* database 2.0 [45]. Gene annotation is as in *T. reesei* database 2.0. The expression data of the genes in the induction dataset with cellulose, wheat and spruce material, and sophorose is shown as the expression cluster number (Mfuzz) and fold change of the transcript signals in the induced cultures as compared to the uninduced control cultures at the same time point. The intensity of the red color and blue color indicates the strength of positive and negative fold changes as compared to the uninduced control cultures, respectively. AV, Avicel cellulose; SO, sophorose; SP, spruce; WH, wheat straw.

The CAZy genes are not randomly positioned in the genome. It has been reported that 41% of CAZy genes are found in 25 discrete regions ranging from 14 kb to 275 kb in length, and cases of co-expressed adjacent or nearly adjacent genes have been shown [7]. The regions of high CAZy gene density were found to contain genes encoding proteins involved in secondary metabolism. Our study also revealed the presence of regulatory genes in close vicinity to CAZy genes. In some cases, co-expression of these regulatory genes with CAZy genes was also detected. This information was used in the selection of candidate regulatory genes for further studies. For example, genes 76677 and 121130 are located in a broad, partly co-regulated region containing several CAZy genes. These genes include a candidate GH27 α-galactosidase gene (59391), a candidate GH2 β-mannosidase gene (59689) and characterized β-glucosidase (*bgl1*) and β-xylosidase (*bxl1*) genes (data not shown). Gene 102499 has an interesting location between a very tightly co-regulated region of CAZy genes and putative secondary metabolism genes (Figure 2, region 1). Gene 120120 is located in a co-expressed region including four genes of the hemicellulase gene-enriched cluster (cluster 35), and close to a second co-expressed region containing the candidate regulatory genes 74765, 55422 and the repressor gene *cre1* (Figure 2, region 2).

Interestingly, we found several loci where a β-glucosidase and/or putative sugar transporter gene is located next to a gene with a putative regulatory function and co-expressed with it. Genes 77513, 105263 and 121121 are located next to candidate β-glucosidase genes *cel1b*, *cel3e* and *cel3d*, respectively. The regions including genes 77513, 121121 and 26163 (the closest homologue for *N. crassa clr-2*) contain a putative sugar transporter gene (Figure 2).

The focus in selection of candidate regulatory genes for further studies was on the genes encoding putative transcription factors. The selected genes fulfilled several of the following criteria: induction by three or more of the cellulase- or hemicellulase-inducing substrates used in the study; co-clustering with the characterized cellulase and hemicellulase genes in the Mfuzz clustering of the expression data; correlation of the expression signal with specific protein production rate in the chemostat study [14]; increased signal of the corresponding protein under good protein-producing conditions in proteome analysis of the chemostat cultures [14]; and co-localization with cellulase and hemicellulase genes in the genome and, preferably, also co-expression of the co-localized genes. In addition, representatives of genes with functional domains indicating different regulatory functions and fulfilling the same criteria were selected. Altogether 28 genes were selected for further studies (Table 2).

**Table 2:** Putative regulatory genes chosen for further studies and the functional domains present in the encoded proteins

**Gene ID**	**Construct**	**InterPro ID**	**Description**
108381	pMH8	IPR001138	Fungal transcriptional regulatory protein, N-terminal
26163	pMH9	IPR001138, IPR007219	Fungal transcriptional regulatory protein, N-terminal; Transcription factor, fungi
121121	pMH10	IPR001138	Fungal transcriptional regulatory protein, N-terminal
70351	pMH11	IPR001138	Fungal transcriptional regulatory protein, N-terminal
102499	pMH12	IPR001138, IPR007219	Fungal transcriptional regulatory protein, N-terminal; Transcription factor, fungi
62244	pMH13	IPR001138, IPR007219	Fungal transcriptional regulatory protein, N-terminal; Transcription factor, fungi
111742	pMH14	IPR001138,	Fungal transcriptional regulatory protein, N-terminal,
77513	pMH15	IPR007219	Transcription factor, fungi
105263	pMH16	IPR001138	Fungal transcriptional regulatory protein, N-terminal
112524	pMH17	IPR001138, IPR007219	Fungal transcriptional regulatory protein, N-terminal; Transcription factor, fungi
123668	pMH18	IPR000182	GCN5-related N-acetyltransferase
73792	pMH19	IPR001138	Fungal transcriptional regulatory protein, N-terminal
80291	pMH20	IPR001138, IPRO007219	Fungal transcriptional regulatory protein, N-terminal; Transcription factor, fungi
107858	pMH21	IPR001138	Fungal transcriptional regulatory protein, N-terminal
120120	pMH22	IPR000182	GCN5-related N-acetyltransferase
47317	pMH24	IPR001138, IPR007219	Fungal transcriptional regulatory protein, N-terminal; Transcription factor, fungi
74765	pMH25	IPR001487, IPR000210	Bromodomain; BTB/POZ-like
76677	pMH26	IPR001138, IPR007219	Fungal transcriptional regulatory protein, N-terminal; Transcription factor, fungi
55422	pMH27	IPR011009	Protein kinase-like domain
121130	pMH28	IPR001138	Fungal transcriptional regulatory protein, N-terminal
122523	pMH29	IPR001138	Fungal transcriptional regulatory protein, N-terminal
123019	pMH30	IPR000719	Protein kinase, catalytic domain
54703	pMH32	IPR007087	Zinc finger, C2H2-type
56077	pMH33	IPR001138	Fungal transcriptional regulatory protein, N-terminal
60215	pMH34	IPR001138	Fungal transcriptional regulatory protein, N-terminal
66966	pMH35	IPR001680	WD40 repeat
64608	pMH36	IPR001680	WD40 repeat
81972	pMH37	IPR001251	Cellular retinaldehyde-binding/triple function, C-terminal

Gene IDs are as in *T. reesei* database 2.0.

**Figure 3:**
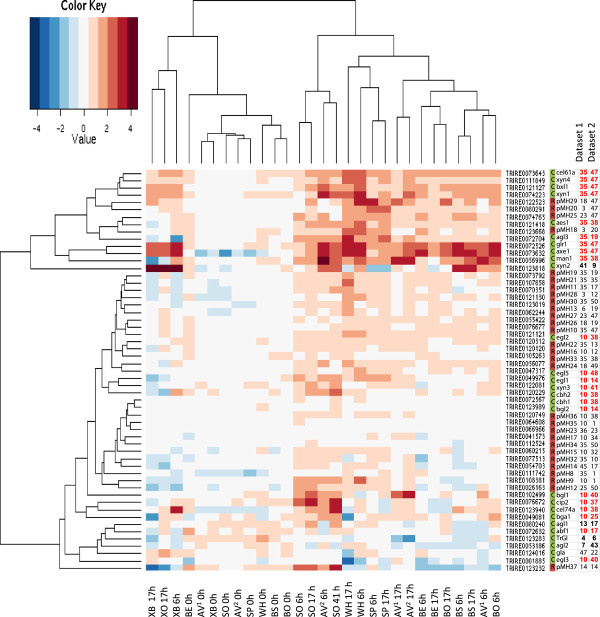
**Heat map visualization of expression data on the known cellulase and hemicellulase genes and the putative regulatory genes in cultures induced with different lignocellulose substrates.** The color key indicates the log2 scale fold change of the transcript signals in the induced cultures versus the uninduced control cultures at the same time point. The genes are shown as rows and the samples as columns. The legend on the right shows the gene ID and the cluster membership of the gene in Mfuzz clustering of the expression datasets. Dataset 1: Induction experiment with Avicel cellulose (0.75%), pretreated wheat straw, pretreated spruce or sophorose; Dataset 2: Induction experiment with Avicel cellulose (1%), bagasse, or xylans [8]. C: CAZy gene, R: regulatory gene. The legend below indicates the lignocellulose substrate in the culture and time point after addition of the substrate. AV^1^, 0.75% Avicel cellulose; AV^2^, 1% Avicel cellulose; BE, enzymatically hydrolyzed bagasse material; BO, untreated bagasse material; BS, steam-exploded bagasse material; SO, sophorose; SP, spruce; WH, wheat straw; XB, birch xylan; XO, oat spelt xylan.

The expression profiles of the selected candidate regulatory genes together with characterized cellulase and hemicellulase genes are represented as a heatmap in Figure 3. The heatmap shows fold change data of the signals in the induced cultures versus the signals in the uninduced cultures at the corresponding time points. Expression values of an additional dataset on cultures induced with a broader set of lignocellulose material (differently pretreated bagasse, oat spelt and birch xylans [8]) are also included. In the heatmap, the candidate regulatory genes are divided into three major groups. Genes 122523, 80291, 74765 and 123668 are co-expressed together with the gene cluster containing many of the known hemicellulase genes (cluster 35). The genes are moderately induced in the presence of the majority of the substrates used, but especially on wheat and spruce. The second group of candidate regulatory genes showed modest induction by the majority of the substrates (IDs 73792, 107858, 70351, 121130, 123019, 62244, 55422, 76677, 121121 and 56077). The third group clusters together with many of the genes in the cellulase-enriched cluster (cluster 10). This group includes genes induced mainly by sophorose, Avicel cellulose, wheat or spruce, but not with bagasse material, and genes hardly induced at all. Detailed transcriptional data of the genes is presented in Additional file 1.

### Primary screening of the effects of the candidate regulatory genes on the cellulase and xylanase production of *T. reesei*

**Figure 4:**
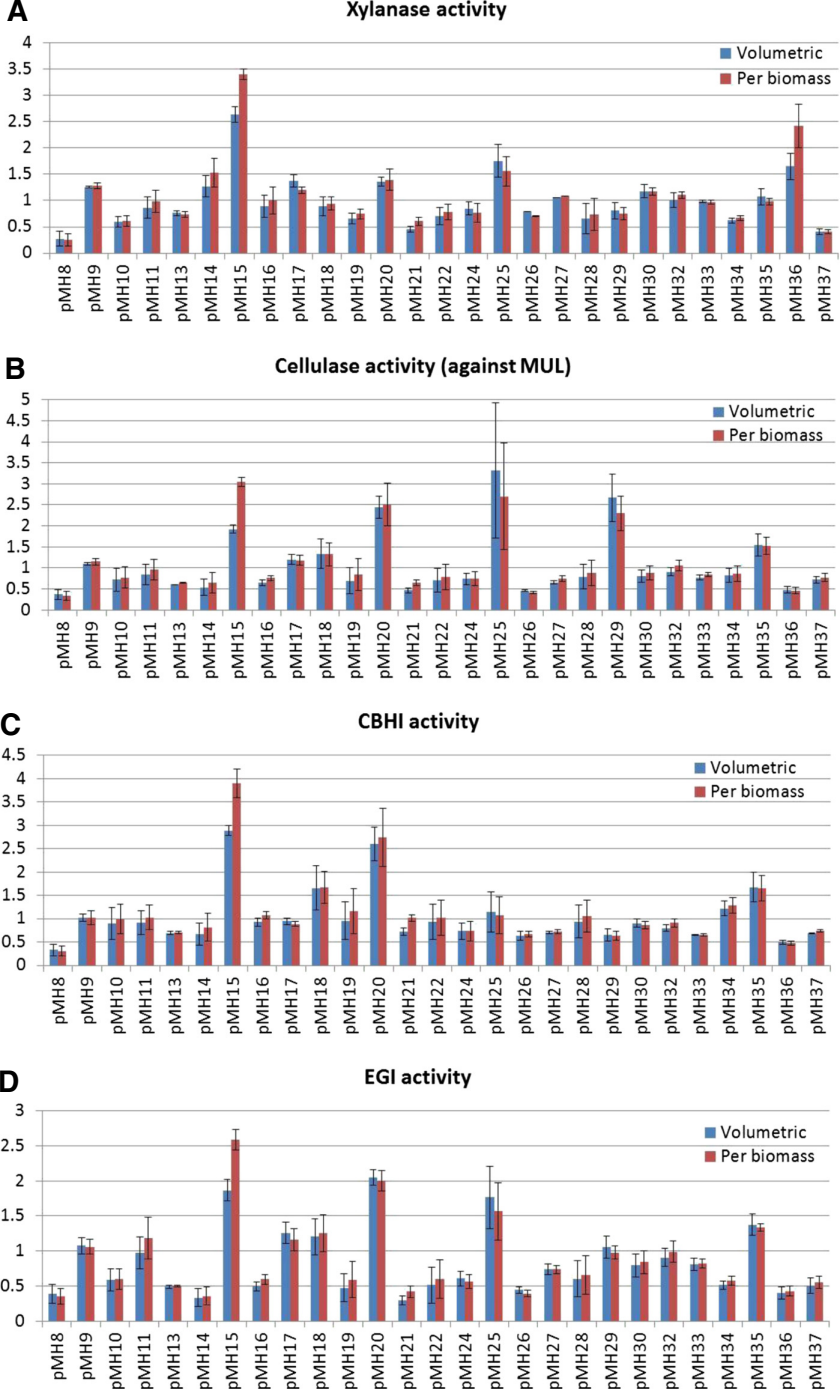
**Cellulase and xylanase production by*T. reesei*QM9414 recombinant strains overexpressing the candidate regulatory genes.** The volumetric enzyme production (blue bars) and production per biomass dry weight (red bars) are shown as the fold change of the maximum amount of activity produced in the cultures of the recombinant strains as compared to the maximum activity produced in the cultures of the parental strain. The values are means of three biological replicates. Error bars show the standard error of the mean. Panels **A** and **B** show the total xylanase activity against birch glucuronoxylan substrate and cellulase activity against 4-methylumbelliferyl-β-D-lactoside substrate, respectively. Panels **C** and **D** show the specific enzymatic activity produced by cellobiohydrolase 1 and endoglucanase 1. Detailed time course data on enzyme production in the cultures is shown in the Additional file 2. CBHI, cellobiohydrolase 1; EGI, endoglucanase 1; MUL, 4-methylumbelliferyl-β-D-lactoside.

In order to investigate the effects of the putative regulatory genes chosen from the data, *T. reesei* QM9414 strains overexpressing the genes were constructed. The genes were cloned to an expression vector under the *A. nidulans gpdA* promoter and the expression plasmids were transformed to QM9414. A β-glucan plate assay was used for preliminary evaluation of enzyme production by the transformants and for selection of representative clones from the transformation for further analysis. The recombinant strains were cultivated in shake flasks on lactose containing rich medium to analyze the effect of the genetic modification on growth and protein production. Produced cellulase and xylanase activities (Figure 4) were measured throughout the cultivation. The growth of the strain transformed with the construct pMH12 was clearly defective as compared to the parental strain and to other recombinant strains, and was therefore omitted from further studies. The enzyme activity produced during the cultivation of the recombinant strains as compared to the activity produced in the cultures of the parental strain is summarized in Figure 4. Detailed information on production of the enzymatic activities during the time course of cultivation is shown in Additional file 2.

**Figure 5:**
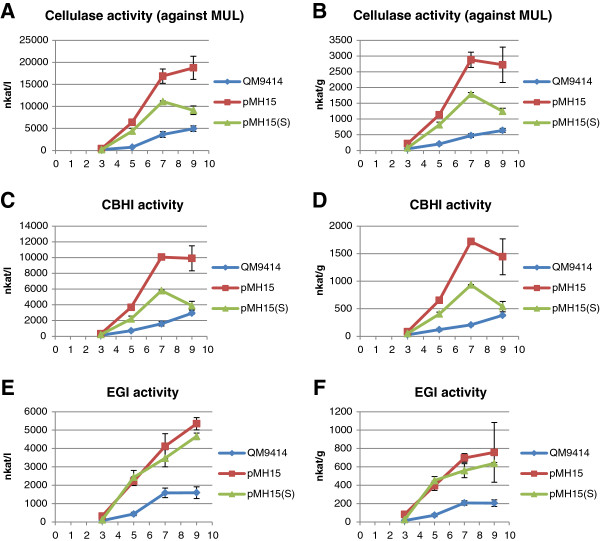
**Production of cellulase activity by two different transformants overexpressing gene 77513.** Transformants harboring the overexpression cassette as a single-copy (pMH15(S)) or as a double-copy (pMH15) were cultivated in shake flasks with lactose as a carbon source. Enzyme activity was measured at four different time points (3, 5, 7 and 9 days). The values are means of three biological replicates. Error bars show the standard error of the mean. Panels **A** and **B** show the volumetric and production per biomass dry weight of total cellulase activity against MUL substrate, respectively. Panels **C-F** show the specific enzymatic activity produced by CBHI and EGI. CBHI, cellobiohydrolase 1; EGI, endoglucanase 1; MUL, 4-methylumbelliferyl-β-D-lactoside.

**Figure 6:**
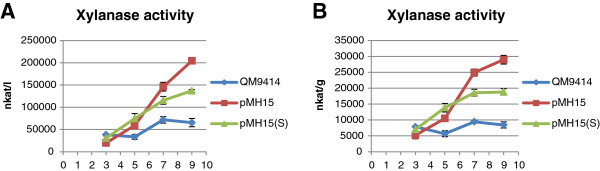
**Production of xylanase activity by two different transformants overexpressing gene 77513.** Transformants harboring the overexpression cassette as a single-copy (pMH15(S)) or as a double-copy (pMH15) were cultivated in shake flasks with lactose as a carbon source. Xylanase activity was measured at four different time points (3, 5, 7 and 9 days). The values are means of three biological replicates. Error bars show the standard error of the mean. Panels **A** and **B** show the volumetric and production per biomass dry weight of xylanase activity, respectively.

The strains overexpressing genes 77513, 74765, 80291, 66966, 123668, 64608 and 122523 (constructs pMH15, pMH25, pMH20, pMH35, pMH18, pMH36 and pMH29) produced cellulase and/or xylanase activity over 1.5-fold as compared to the parental strain in the shake flask cultures. The integrity of these seven strains and overexpression of the genes were confirmed by southern and northern blot analysis, respectively (Additional files 3 and 4). Most of the modified strains tested had the overexpression construct integrated as a single copy. The strain overexpressing the construct pMH35 had one to two copies according to the Southern hybridization. For the construct pMH15, both a single-copy and a double-copy transformant were analyzed (Figures 5 and 6). Northern analysis showed 1.4- to 23.6-fold overexpression of the gene for the strains analyzed (Additional file 4), except for gene 123668 (pMH18), which was expressed at a low level both in the overexpression strain and in the parental strain and therefore was not quantified. In addition, a number of the recombinant strains (transformed with constructs pMH8, pMH13, pMH21, pMH22, pMH24, pMH26 and pMH37) produced clearly less enzymatic activity than the parental strain. These genes were omitted from further studies.

Overexpression of gene 77513 (construct pMH15) had the most consistent and statistically significant (t-test; *P* <0.05) positive effect on both cellulase and xylanase production by *T. reesei*. The strain produced in the initial screening 3- to 4-fold cellobiohydrolase 1 (CBHI) activity, 2- to 2.5-fold endoglucanase 1 (EGI) activity and 2- to 3-fold combined activity as measured against the 4-methylumbelliferyl-β-D-lactoside (MUL) substrate (Figure 4). The strain also produced 2- to 3-fold more xylanase activity as measured against the parental strain.

The strain overexpressing gene 80291 (construct pMH20) produced 2.5-times more CBHI activity, 2-times more EGI activity and 2.5-times more total activity against the MUL substrate. However, the xylanase activity was only slightly improved in this recombinant strain (less than 1.5-fold) as compared to the parental strain. The change in the production levels by pMH20 overexpression was statistically significant (t-test; *P* <0.05).

The overexpression of gene 74765 (construct pMH25) produced the largest amount of cellulase activity as measured volumetrically against the substrate MUL, as compared to the other recombinant strains and to the parental strain (almost 3.5-times more than the parental strain). Production of xylanase activity was also increased more than 1.5 times in the recombinant strain. However, *T. reesei* EGI (CEL7B) has been shown to have activity against xylans as well and thus the increase in xylanase activity could be partly due to the increase in EGI production [33].

### Quantitative PCR of cellulase and hemicellulase genes

**Figure 7:**
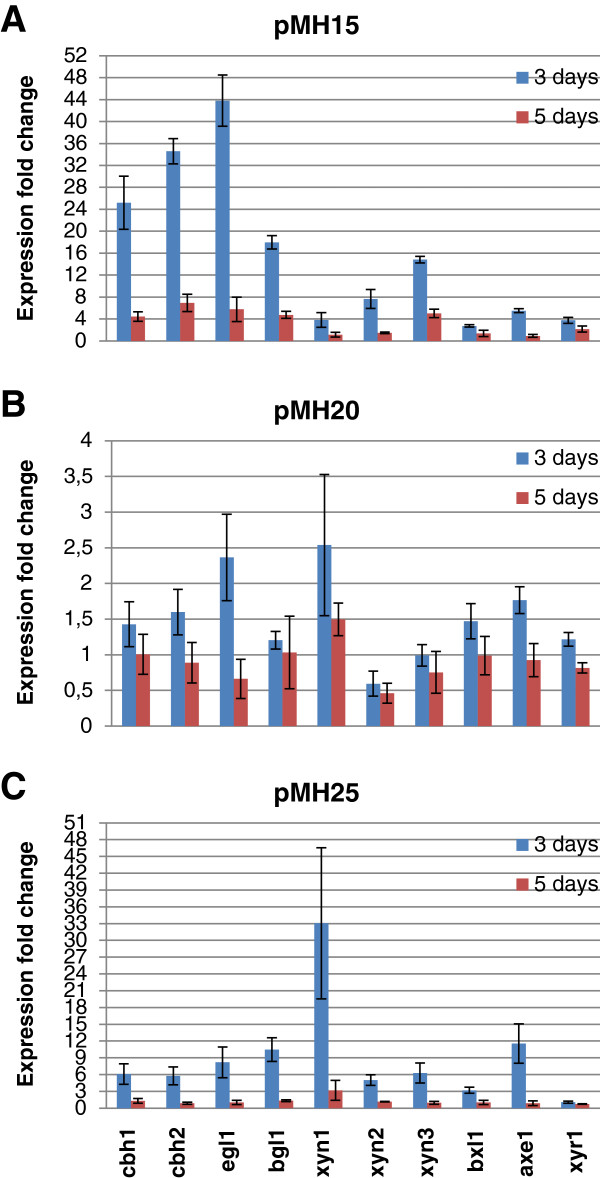
**Quantitative PCR analysis of cellulase and hemicellulase gene expression of strains overexpressing the constructs pMH15, pMH20 and pMH25.** Panels **A**, **B** and **C** show the expression levels of the analyzed genes for strains overexpressing the constructs pMH15, pMH20 and pMH25, respectively. Expression levels are normalized against the signal of *sar1* and are shown as a fold change as compared to the normalized expression level in the parental strain. RNA extracted after 3 days (blue bars) and 5 days (red bars) of cultivation was used as a template. The values are means of three biological replicates. Error bars show the standard error of the mean.

Based on the preliminary enzyme activity measurements, strains overexpressing genes 77513, 80291 and 74765 (constructs pMH15, pMH20 and pMH25) were selected for further studies. For clarity, the recombinant strains will be referred to by the construct names. A quantitative PCR analysis of *axe1*, *bxl1*, *xyn1*, *xyn2*, *xyn3*, *cbh1*, *cbh2, egl1, bgl1* and *xyr1* was carried out. The results are shown as a fold change of the signals as compared to the parental strain QM9414 (Figure 7). For all the strains, the expression of *cbh1*, *cbh2* and *egl1* was improved as compared to the parental strain, although for pMH20 and pMH25 the effect was more moderate and was detected for pMH20 only at the 3-day time point. The expression of the major β-glucosidase gene *bgl1* was clearly improved by the pMH15 and pMH25 constructs but not by pMH20. Similarly, the expression of the three xylanase genes was improved by pMH15 and pMH25. Regarding xylanase gene expression, the overexpression of gene 77513 (pMH15) seemed to have most effect on *xyn3*, whereas the two other candidate regulatory genes were more specific to *xyn1* (only xylanase gene with improved expression by pMH20). Particularly, overexpression of gene 74765 (pMH25) had a major effect on the transcription of *xyn1*. The expression of *bxl1* was moderately improved with pMH15 and pMH25. The clearest increase in *axe1* expression was seen with pMH25. The expression of *xyr1*, which encodes the major regulator of cellulase and hemicellulase genes, was higher in pMH15 than in the parental strain but was not affected in the other two strains.

### Overexpression and deletion of gene 77513, designated as *ace3*

Based on the quantitative PCR and enzyme production results of the recombinant strain overexpressing the construct pMH15, gene 77513 was selected for more detailed studies. A recombinant strain was constructed from which gene 77513 was deleted (designated Del77513). We also analyzed enzyme production by strains having both one (pMH15(S)) or two (pMH15) copies of the overexpression cassette and in the 77513 deletion strain (all the constructs were confirmed by Southern and Northern analyses, Additional files 3 and 4). Both overexpression strains were cultivated in parallel with the deletion strain and the parental strains. Produced cellulase activity against the MUL substrate and xylanase activity were measured throughout the cultivation.

**Figure 8:**
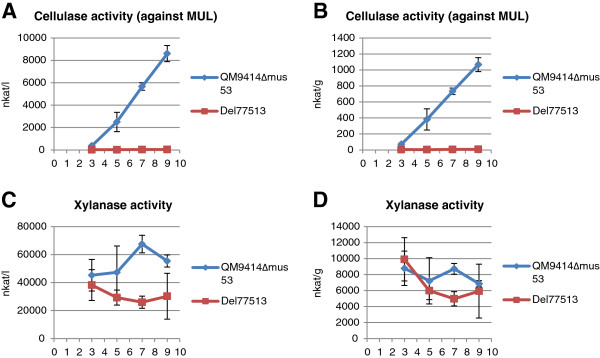
**Production of cellulase and xylanase activity by the 77513 deletion strain.** Del77513 was cultivated in shake flasks in lactose containing medium in parallel with the parental strain QM9414Δmus53. Enzyme activity was measured at four different time points (3, 5, 7 and 9 days). The values are means of three biological replicates. Error bars show the standard error of the mean. Panels **A** and **B** show the volumetric and production per biomass dry weight of total cellulase activity against MUL substrate, respectively. Panels **C** and **D** show the volumetric and production per biomass dry weight of xylanase activity, respectively. MUL, 4-methylumbelliferyl-β-D-lactoside.

Both overexpression strains produced significantly (t-test; *P* <0.05) more total MUL activity, CBHI, EGI and xylanase activity as compared to the parental strain (Figures 5 and 6). The improvement in cellulase and xylanase production was higher in the double-copy strain than in the single-copy strain, indicating that the possible double-integration of the expression cassette also amplified the positive effect of the overexpressed gene to cellulase and xylanase production. When gene 77513 was deleted, the production of total cellulase activity against the MUL substrate was abolished completely (Figure 8). Interestingly, production of xylanase activity decreased to approximately half that of the parental strain (most significant decrease at day 7), indicating that gene 77513 is not essential for the production of xylanase activity but does modulate it (Figure 8).

**Figure 9:**
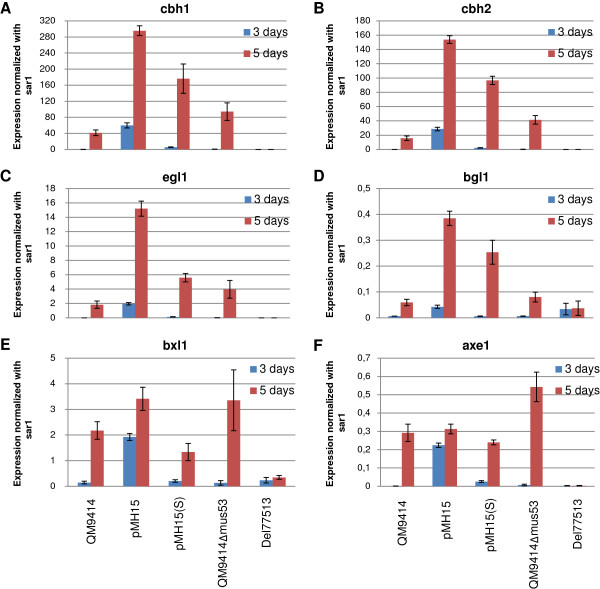
**Quantitative PCR analysis of cellulase and hemicellulase gene expression by the strains overexpressing gene 77513 and by the strain with gene 77513 deleted.** pMH15 is harboring the expression cassette as a double-copy and pMH15(S) as a single-copy. Panels **A**, **B**, **C**, **D**, **E** and **F** show the expression levels of *cbh1*, *cbh2*, *egl1*, *bgl1*, *bxl1* and *axe1* genes, respectively. Expression levels are shown as normalized against the signal of *sar1*. RNA extracted after 3 days (blue bars) and 5 days (red bars) of cultivation was used as a template. The values are means of three biological replicates. Error bars show the standard error of the mean.

**Figure 10:**
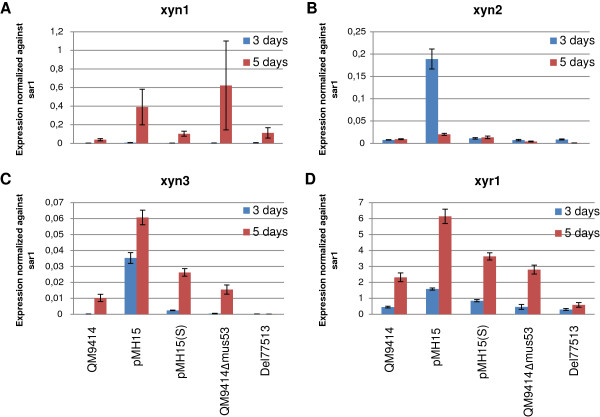
**Quantitative PCR analysis of xylanase and*xyr1*gene expression by the strains overexpressing gene 77513 and by the strain with gene 77513 deleted.** pMH15 is harboring the expression cassette as a double-copy and pMH15(S) as a single-copy. Panels **A**, **B**, **C** and **D** show the expression levels of *xyn1*, *xyn2*, *xyn3* and *xyr1* genes, respectively. The expression levels are shown as normalized against the signal of *sar1*. RNA extracted after 3 days (blue bars) and 5 days (red bars) of cultivation was used as a template. The values are means of three biological replicates. Error bars show the standard error of the mean.

A quantitative PCR analysis of *axe1*, *bxl1*, *xyn1*, *xyn2*, *xyn3*, *cbh1*, *cbh2, egl1, bgl1* and *xyr1* was carried out for samples collected from the cultivation of strains pMH15, pMH15(S) and Del77513. Due to the different parental strains of the overexpression strains and the deletion strain, the results are shown normalized with the signal of *sar1* (Figures 9 and 10). The expression of *cbh1*, *cbh2*, *egl1*, *bgl1*, *xyn1*, *xyn2*, *xyn3* and *xyr1* was higher in the overexpression strains as compared to the parental strain. In accordance with the enzymatic activity measurements, the increase in the gene expression was higher in the double-copy strain than in the single-copy strain. The expression of *bxl1* was improved only in the double-copy strain.

Expression of *cbh1*, *cbh2*, *egl1*, *axe1* and *xyn3* was almost undetectable in the deletion strain as compared to the parental strain. The expression of *bxl1*, *xyn1*, *xyn2, bgl1* and *xyr1* was also lower as compared to the parental strain. In the light of the enzymatic activity and quantitative PCR results for the two strains overexpressing gene 77513 and for the strain with the gene deleted, this gene was named *activator of cellulase expression 3 (ace3*).

## Discussion

The double-lock gene regulation mechanism, in which a master transcription factor regulates an additional trans-acting regulatory factor gene together with its actual target genes, is well-documented in filamentous fungi. In particular, carbon catabolite repression has been reported to be mediated by such a mechanism. In the model organism *A. nidulans*, the carbon catabolite repressor CREA regulates the ethanol utilization genes by repressing both the positively acting regulatory gene *alcR* and its target, *alcA*[34]. CREA also regulates lignocellulolytic genes by repressing the major activator (*xlnR*) as well as many of its target genes, for example, *xlnD* and *xlnB*[35]. Similarly, the major regulator of cellulolytic and xylanolytic genes in *T. reesei* (*xyr1*, a homologue of *xlnR)* is repressed by the carbon catabolite repressor CRE1 together with many *xyr1* target genes [18,36].

In this study, we utilized the principle of the double-lock mechanism to find new regulators of cellulase and hemicellulase genes, presuming that these regulators would be regulated in a similar manner as their target genes. We analyzed transcriptome data from *T. reesei* cultures induced with different lignocellulose-derived substances to search for candidate regulatory genes. This led to identification of 89 candidate genes that were co-induced with many of the known cellulase or hemicellulase genes in the presence of different lignocellulose-derived materials. We selected 28 genes for overexpression screening by taking into account supporting evidence from other genome-wide datasets, such as transcriptome and proteome analysis of chemostat cultures with different protein production rates [14], as well as location of the genes in the genome.

Clustering of the biosynthesis genes for fungal secondary metabolites together with their regulatory genes in the genome, as well as the regulatory cascades including chromatin-mediated regulation of the genomic regions, is relatively well-characterized in fungi (for a review, see [37]). Recent studies have indicated that chromatin level regulation also takes place in the regulation of CAZy genes of *T. reesei*. The putative methyltransferase LAE1, a homologue of LaeA functioning in chromatin level regulation of secondary metabolism in *Aspergilli*, has been shown to be involved in controlling cellulase gene expression in *T. reesei,* although the actual mechanism is not fully understood [38]. Furthermore, genes with significant up- or down-regulation during conidiation [39] as well as genes whose expression levels correlate with the specific production rate of extracellular proteins [14] have been shown to be non-randomly distributed in the *T. reesei* genome. Genes encoding, for example, secondary metabolism proteins, CAZys, putative transporters and putative transcription factors have been identified from such genomic clusters. In addition, the protein families of these regulators and the protein families of CAZys and secondary metabolism-related enzymes have recently expanded in the evolution of filamentous fungi, (*Pezizomycotina)*[40]. Thus, positioning of the regulatory genes in the close vicinity of their target genes (or other genes involved in the same process) may not be limited to the secondary metabolism genes, but could involve the genes active in lignocellulose degradation as well.

The transcriptome data on the cultures induced with different lignocellulosic material showed genomic regions that are co-regulated in an inducer-specific manner. Of the genes that were co-expressed with the major cellulase and hemicellulase genes according to the Mfuzz clustering, 22.7% were located in enriched genomic regions (≥ three genes within a window of nine genes, with a maximal distance of five genes). Of these, 9.1% (32 genes) were located next to each other in patches of three or more genes and were tightly co-regulated.

In addition to the known regulatory gene for hemicellulase and cellulase genes, *xyr1*, nine candidate regulatory genes were located in these tightly co-regulated regions or within close vicinity (Figure 2). Interestingly, four of the genes were located next to a putative sugar transporter and/or a β-glucosidase gene. In addition to the release of glucose from cellobiose by extracellular β-glucosidases and transport of sugars into the cells, the sugar transporters and β-glucosidases may have a special role in the onset of the CAZy gene induction. Sugar units derived from the complex carbon source may be transported inside the cells and further modified by intracellular β-glucosidases to form an inducing compound, such as sophorose via a transglycosylation reaction. The gene *cel3e,* located next to gene 105263 (pMH16), encodes a predicted extracellular β-glucosidase. By contrast, *cel3d* and *cel1b,* located next to genes 121121 (pMH10) and 77513/*ace3* (pMH15), respectively, are predicted to encode intracellular enzymes. Interestingly, the sugar transporter genes located next to genes *ace3* and 26163 have recently been suggested to be involved in lactose uptake and cellulase production in lactose-containing media [41,42].

Co-location of a putative regulatory gene with a β-glucosidase gene and a transporter gene is not a unique feature of the *T. reesei* genome. For example, the homologues of 77513/*ace3* (pMH15) in *A. fumigatus* (AFUA_016410) and in *A. clavatus* (ACLA_01970) are accompanied by a candidate β-glucosidase gene (AFUA_1G16400/ACLA_01980) and a candidate hexose transporter gene (AFUA_1G16390/ACLA_019190) next to it in the genome. Similarly, the homologues of gene 121121 (pMH10) in *A. fumigatus* (AFUA_7G00210) and in *A. nidulans (*ANIA_02615) are located next to a candidate hexose transporter gene (AFUA_7G00220/ANIA_02614), a candidate major facilitator superfamily multidrug transporter gene (AFUA_7G2613/ANIA_02614), and a β-glucosidase gene (AFUA_7G00240/ANIA_026142) [43].

In a recent study, it was suggested that, in *N. crassa*, the cellulase/hemicellulase regulator CLR-1 would promote the expression of cellodextrin transporters and β-glucosidase genes as well as a second regulatory gene, *clr-2,* which in turn activates cellulase genes [25]. In *N. crassa*, *clr-2* is essential for cellulase production in the presence of Avicel cellulose [25]. In *T. reesei*, the homologue of *clr-2,* gene 26163 (construct pMH9), is located next to a co-regulated sugar transporter gene that has recently been described as a lactose permease essential for the induction of *cbh1* and *cbh2*[42]. Overexpression of gene 26163 alone resulted only in a minute enhancement in production of cellulase and xylanase activity. However, no close homologue for *clr-1* can be identified from *T. reesei*, suggesting an important difference in the activation mechanisms of *clr-2*/26163 and/or the accompanying transporter genes in *N. crassa* and in *T. reesei*.

Overexpression of genes 105263 (pMH16) and 121121 (pMH10) did not have a significant effect on protein production under the conditions studied. However, overexpression of *ace3*, which is located next to a co-regulated β-glucosidase gene (*cel1b)* and a candidate sugar transporter gene in its original locus, resulted in a significantly increased production of cellulase and xylanase activity as compared to the parental strain. Deletion of the gene was detrimental to the production of cellulase activity and decreased the production of xylanase activity. Quantitative PCR analysis of transcript levels of cellulase and xylanase genes supported the enzymatic activity measurements. Therefore, *ace3* can be considered to code for a novel master regulator of cellulase expression and a modulator of xylan degrading enzyme expression. Thus its role appears to be different from that of XYR1/XlnR, which has a major role in both xylan and cellulose degradation [18,44]. Interestingly, the Mfuzz clustering of *ace3* reflects the quantitative PCR results to some extent. The gene clustered together with *egl1*, *cbh1*, *cbh2*, *bgl1* and *xyn3, which* were most affected by *ace3* modifications, whereas *axe1*, *bxl1*, *xyn1* and *xyn2* are in different clusters.

Transcription of *xyr1* was increased in the strains overexpressing *ace3* and decreased in the deletion strain, indicating that the effects on the target genes observed could be at least partly mediated via *xyr1*. However, the deletion of *ace3* did not totally abolish *xyr1* transcription. Therefore, the absence of XYR1 is not an explanation for the total lack of cellulase activity and gene expression exhibited by the deletion strain.

## Conclusions

Combining genome-wide data on cultures with different protein production properties is a useful method for identifying novel regulatory genes relevant for cellulase and xylanase production in *T. reesei*. Altogether, overexpression of seven of the candidate regulatory genes resulted in improved (>1.5 fold) production of cellulase and/or xylanase activity as compared to the parental strain. Further studies are required to confirm the role of most of these genes in cellulase and hemicellulase gene regulation and to elucidate the actual regulatory mechanisms. However, our data show a positive effect of cellulase and/or xylanase gene expression for three of the candidate regulatory genes. The deletion of one of these genes, *ace3*, totally abolished cellulase expression and reduced xylan degrading enzyme expression, thus identifying it as a novel master regulator of lignocellulose degradation. Furthermore, our data reveal genomic regions enriched in co-regulated CAZy genes and candidate regulatory genes, therefore supporting the hypothesis that chromatin-level regional regulation plays a role, at least in part, in the expression of CAZy genes in *T. reesei*.

## Methods

### Strains, media and culture conditions

*Escherichia coli* DH5α (*fhuA2* Δ*(argF-lacZ)U169 phoA glnV44 Φ80* Δ*(lacZ)M15 gyrA96 recA1 relA1 endA1 thi-1 hsdR17*) was used for propagation of the plasmids. *T. reesei* Rut-C30 (ATCC 56765, VTT-D-86271), QM6a (ATCC13631, VTT-D-071262 T) and QM9414 (ATCC 26921, VTT-D-74075) were obtained from VTT Culture Collection (Espoo, Finland). Spore suspensions were prepared by cultivating the fungus on potato-dextrose plates (BD, Sparks, Maryland, USA ) for 5 days, after which the spores were harvested, suspended in a buffer containing 0.8% NaCl, 0.025% Tween20 and 20% glycerol, filtered through cotton, and stored at −80°C. For DNA isolation, the fungus was grown in a medium containing 0.2% proteose peptone (BD), 2% glucose, 7.6 g/l (NH_4_)_2_SO_4_, 15.0 g/l KH_2_PO_4_, 2.4 mM MgSO_4_.7H_2_O, 4.1 mM CaCI_2_.H_2_O, 3.7 mg/l CoCI_2_, 5 mg/l FeSO_4_.7H_2_O, 1.4 mg/l ZnSO_4_.7H_2_O and 1.6 mg/l MnSO_4_.7H_2_O, pH 4.8.

### Transcriptional profiling data

Transcriptional profiling data used in the study have been described elsewhere [8]. In short, pre-cultures of *T. reesei* Rut-C30 were first cultivated on a minimal medium containing sorbitol as a carbon source. Cellulase and hemicellulase gene expression was induced by addition of different lignocellulose material, purified lignocellulose-derived polymers or specific disaccharides (Cultivation set 1: addition of Avicel cellulose, pretreated wheat straw, pretreated spruce or sophorose; Cultivation set 2: addition of Avicel cellulose, birch xylan, oat spelt xylan, or differentially pretreated bagasse). Wheat straw and spruce were pretreated using steam explosion. Three different pretreatment methods were applied to bagasse, including grinding of the untreated bagasse material, steam explosion, or steam explosion followed by enzymatic treatment. Enzymatic pretreatment was done with a commercial cellulase and hemicellulase mixture followed by a protease treatment. Samples for transcriptional profiling were collected at different time points of induction (0, 6 or 17 h).

Custom-made microarray slides from RocheNimbleGen were used for transcriptional profiling. Sample preparation, hybridization onto microarray slides and collection of raw data was carried out as instructed by Roche. The microarray data were analyzed using the R package Oligo for preprocessing of the data and the package Limma for identifying differentially expressed genes [28,32]. In the analysis of the differentially expressed genes, the signals in the samples of the induced cultures were compared to the ones in the uninduced control cultures at the corresponding time point as described in [8]. Four biological replicates of each condition and time point were analyzed. The cut-off used for statistical significance was *P* <0.01, and an additional cut-off for the log2 scale fold change was set as 0.4. In addition, the expression array datasets were clustered using the R package Mfuzz [29]. Co-expressed genomic clusters were determined by enrichment of Mfuzz cluster members in the genomic regions. Three or more gene members of the expression cluster within a window of nine neighboring genes and with the maximal distance of five genes were considered as a genomic region enriched with co-regulated genes. In addition, genomic regions with multiple adjacent genes belonging to the same expression cluster were searched for.

The expression of the selected candidate regulatory genes was compared to the transcriptome and proteome data described in [14].

### Construction of *T. reesei* strains overexpressing candidate regulatory genes

The regulatory genes were amplified by PCR using Gateway compatible primers (Table 3) and the genomic DNA of *T. reesei* QM6a as a template. For the majority of the genes, the open reading frame (ORF) predictions used were as in the genome version 2.0 [45] with the following exceptions: the primers for genes 26163 and 64608 and the N-terminal primer for gene 47317were designed according to the ORF prediction in archived genome version 1.0 [46], and the ORF prediction for gene 64608 was modified by taking into account expressed sequence tag sequence data. In order to construct the plasmid vectors for overexpression of the genes in *T. reesei*, the PCR fragments were inserted in the expression vector pMS204 using the Gateway recombination system (One-Tube protocol) according to the manufacturer’s instructions (Invitrogen, Carlsbad, California, USA). The expression vector contains the hygromycin resistance gene (ZP_12918108) under the *A. nidulans gpdA* promoter [47] and *trpC* terminator [48], as well as an additional copy of the *gpdA* promoter and *trpC* terminator for expression of the gene of interest (the vector map is illustrated in Additional file 5). The plasmids were linearized using *HindIII*, *PciI* or *SpeI* enzyme (New England BioLabs, Ipswich, Massachusetts, USA) and transformed to *T. reesei* QM9414 by polyethylene glycol-mediated protoplast transformation [49]. The transformants were selected for hygromycin resistance on plates containing 150 μg ml^-1^ of hygromycin B (Calbiochem, San Diego, California, USA). Stable transformants were obtained by streaking on plates containing 125 μg ml^-1^ of hygromycin B for two successive rounds, after which single colonies were obtained by plating dilutions of spore suspensions. Integration was verified by PCR with one primer binding the *gpdA* promoter and one binding the ORF of the overexpressed gene (the primers used are listed in Table 4). The cellulase production levels of transformants from each construct were assayed on β-glucan plates (see below). Southern blot analysis was carried out for additional confirmation of the transformants showing improved protein production as compared to the parental strain. Genomic DNA was isolated using an Easy-DNA Kit (Invitrogen) according to manufacturer’s instructions. Southern blotting and hybridization on nitrocellulose filters (Hybond N, GE Healthcare, Little Chalfont, UK) were carried out according to standard procedures [50]. Probe fragments were PCR-amplified from the genomic DNA. The signals were detected using a phosphorimager (Typhoon imager, GE Healthcare).

**Table 3:** Gateway compatible primers for the cloning of the putative regulatory genes

**Construct**	**5′primer**	**3′primer**
pMH8	5′GGGGACAAGTTTGTACAAAAAAGCAGGCTTGCGCATCATGGCGCTCTTTGTCTGCTTGG	3^′^GGGGACCACTTTGTACAAGAAAGCTGGGTCCTATTGTTGCTGCCCGCCCCA
pMH9	5′GGGGACAAGTTTGTACAAAAAAGCAGGCTACATCATGTTCTACACATGTGG	3′GGGGACCACTTTGTACAAGAAAGCTGGGTTTCACGACGGCGGTAGAGC
pMH10	5′GGGGACAAGTTTGTACAAAAAAGCAGGCTCGCACTAGAGCACAATGGAGAC	3′GGGGACCACTTTGTACAAGAAAGCTGGGTGCTACTTCTGTATACACTTAATCAC
pMH11	5′GGGGACAAGTTTGTACAAAAAAGCAGGCTTGCGCATCATGGCCTCCAATGCCAAC	3′GGGGACCACTTTGTACAAGAAAGCTGGGTTTCATAATCAGACCAGCTCTTTC
pMH12	5′GGGGACAAGTTTGTACAAAAAAGCAGGCTTGCGCATCATGGGGAGCAGCGCCA	3′GGGGACCACTTTGTACAAGAAAGCTGGGTTCTAGCCGTAAATCTATGTAGTTGA
pMH13	5^′^GGGGACAAGTTTGTACAAAAAAGCAGGCTTGCGCATCATGCCACGCCCAAAAGTCCACC	3′GGGGACCACTTTGTACAAGAAAGCTGGGTATCAGAACCCAAACGCCCGCGG
pMH14	5′GGGGACAAGTTTGTACAAAAAAGCAGGCTTGCGCATCATGGCGTCCTCTTACGGCACTC	3′GGGGACCACTTTGTACAAGAAAGCTGGGTGTTAGAATACTAAACTCTTCGC
pMH15	5′GGGGACAAGTTTGTACAAAAAAGCAGGCTTGCGCATCATGCTGCGCTACTCCCCCGTCT	3′GGGGACCACTTTGTACAAGAAAGCTGGGTTTTAGCCAACAACGGTAGTGGA
pMH16	5′GGGGACAAGTTTGTACAAAAAAGCAGGCTTGCGCATCATGACCAGCTCGGACGATTCCA	3′GGGGACCACTTTGTACAAGAAAGCTGGGTCTCAGGTGAAGGAGGGCGGTAT
pMH17	5′GGGGACAAGTTTGTACAAAAAAGCAGGCTTGCGCATCATGGCTGGATCGCCTGCTGCTG	3′GGGGACCACTTTGTACAAGAAAGCTGGGTACACATTCATCCCTGCGCCCAG
pMH18	5′GGGGACAAGTTTGTACAAAAAAGCAGGCTTGCGCATCATGCCTCTCGTTGTCGTCCCAG	3′GGGGACCACTTTGTACAAGAAAGCTGGGTCTTAATTGAGCAGCGGCTCGCG
pMH19	5′GGGGACAAGTTTGTACAAAAAAGCAGGCTTGCGCATCATGGATCTGCAATCCTTTGACA	3′GGGGACCACTTTGTACAAGAAAGCTGGGTCCTACAGACGCTTTCCGAAAAAG
pMH20	5′GGGGACAAGTTTGTACAAAAAAGCAGGCTTGCGCATCATGGGCCGGCAACCGAGACAAC	3′GGGGACCACTTTGTACAAGAAAGCTGGGTATTATATAAACGGGGCATCAAT
pMH21	5′GGGGACAAGTTTGTACAAAAAAGCAGGCTTGCGCATCATGGTTCGAGGCACCGGATC	3′GGGGACCACTTTGTACAAGAAAGCTGGGTTCTAAGAAACATCTTCCGACCTGA
pMH22	5′GGGGACAAGTTTGTACAAAAAAGCAGGCTTGCGCATCATGTCCCGCCAAATCTCC	3′GGGGACCACTTTGTACAAGAAAGCTGGGTCTTACTCGGTGCTGATACTTCT
pMH24	5′GGGGACAAGTTTGTACAAAAAAGCAGGCTTGCGCATCATGTCGTCAAACGCTTCACCG	3′GGGGACCACTTTGTACAAGAAAGCTGGGTCCTAGCCCAAATGGCCCATATTG
pMH25	5′GGGGACAAGTTTGTACAAAAAAGCAGGCTTGCGCATCATGACTTCTGAAGCCCCCTCTC	3′GGGGACCACTTTGTACAAGAAAGCTGGGTCCTACTCGCCCTCTTCGCCTC
pMH26	5′GGGGACAAGTTTGTACAAAAAAGCAGGCTTGCGCATCATGGCCGACACCCCGACTC	3′GGGGACCACTTTGTACAAGAAAGCTGGGTTTTAGAAGCCCGCCTGCTCTGC
pMH27	5′GGGGACAAGTTTGTACAAAAAAGCAGGCTTGCGCACAATGCCTCGCCGCGCCT	3′GGGGACCACTTTGTACAAGAAAGCTGGGTTTCATTCATCGCCCCAGAACAA
pMH28	5′GGGGACAAGTTTGTACAAAAAAGCAGGCTTGCGCATCATGAACATGACGACAACGCT	3′GGGGACCACTTTGTACAAGAAAGCTGGGTTCTATCTATAACTTGGTATTTTGC
pMH29	5′GGGGACAAGTTTGTACAAAAAAGCAGGCTTGCGCATCATGGTAGCACATAGTCTACCCTCT	3′GGGGACCACTTTGTACAAGAAAGCTGGGTCTCATATCGGCACCATGTCG
pMH30	5′GGGGACAAGTTTGTACAAAAAAGCAGGCTTGCGCATCATGCTCATCAACAACCTCGATCC	3′GGGGACCACTTTGTACAAGAAAGCTGGGTGTCAGACGAAACGCCGCCAG
pMH32	5′GGGGACAAGTTTGTACAAAAAAGCAGGCTTGCGCATCATGTTCCCGTACGGTGCC	3′GGGGACCACTTTGTACAAGAAAGCTGGGTCTCACATACCCATAATCATTCCTC
pMH33	5′GGGGACAAGTTTGTACAAAAAAGCAGGCTTGCGCATCATGACATCAATAACGCATCCCTC	3′GGGGACCACTTTGTACAAGAAAGCTGGGTTGCCTTCATCTCCTGGTGGAAT
pMH34	5′GGGGACAAGTTTGTACAAAAAAGCAGGCTTGCGCATCATGTTCGGACAGTACTCACTCG	3′GGGGACCACTTTGTACAAGAAAGCTGGGTATTACGACAATGGCAAGATCCT
pMH35	5′GGGGACAAGTTTGTACAAAAAAGCAGGCTTGCGCATCATGGCCAAGAAGGCGCGTC	3′GGGGACCACTTTGTACAAGAAAGCTGGGTGCTAGGCGCCGTTGACGACTC
pMH36	5′GGGGACAAGTTTGTACAAAAAAGCAGGCTTGCGCATCATGTCCAGATTCTGTCCGCT	3′GGGGACCACTTTGTACAAGAAAGCTGGGTGCATCAATAGGCCGTATCAGAG
pMH37	5′GGGGACAAGTTTGTACAAAAAAGCAGGCTTGCGCATCATGACGGCCGAGTACGAAG	3′GGGGACCACTTTGTACAAGAAAGCTGGGTCTCGTTCATGCAGCAGCAG

**Table 4:** Primers for the PCR screening of the overexpression strains

**Primer**	**Sequence 5′- 3′**
pgpdA	GGCAGTAAGCGAAGGAGAATG
pMH8R1	ACACGGCTTCTTATATCTCGACC
pMH9R3	ATGGTCTCGATGTGGCTGCT
pMH10R1	CTGCGAGAGCAGCTAGGAGC
pMH11R2	CGTCGATTCGCGCTTGAACA
pMH12R2	GATGCACGCCGCCATCGAGT
pMH13R2	TCGTTCTCCTCGTAGATTCAG
pMH14R1	GCTGGCTCTTCTCCCTCACAC
pMH15R3	TGAGTATAGCGGCTGACTTGTCG
pMH16R3	CTCGTTGACTTGCAGGCCTTG
pMH17R1	CTGAGGGCTGTAGACGCACTC
pMH18R1	TTACAGAGGTGAGACTTTCCCT
pMH19R1	TTGCGTTGCGCCTTTACC
pMH20R3	TCGAGACGATGCAGCGATAG
pMH21R1	TGGTTCTGGATCACTCGTCA
pMH22R1	TTCGTCCTCCGTCTTGAGCA
pMH24R2	CTCACCTCGTCGTACACACTA
pMH25R1	ATGCGGTTGACTTGACAGAT
pMH26R2	GGTTGACTCTGGATGTTGGA
pMH27R1	ATCTTGACGTCCTTGTCGAT
pMH28R1	GCGAATCGACCAGATCGTGT
pMH29R1	GTCCTTGCACCGCTTACACG
pMH30R2	GTAGAAGCGCAATGCGGTGG
pMH32R2	CAGATGCACGTCTTCCAGAT
pMH33R1	TCTGGTCTCGATTGCTCGTG
pMH34R1	CATCAGCCTCGTCTCCAGCA
pMH35R3	CATCATCAATGTCCTCGAAG
pMH36R1	GTCAGGATAGCGCCTGTCTG
pMH37R1	GTCCGGTACAGCGTGTCAAT

Primer named pgpdA was used in combination with the gene specific primers.

### Plate assay for β-glucan hydrolysis using Congo red staining

For detection of enzymatic activity against the β-glucan produced by fungal colonies, spores were mixed with 50°C top agar containing 0.1% β-glucan (Megazyme, Bray, Wicklow, Ireland), 2% lactose (Fagron, Rotterdam, the Netherlands), 0.05% proteose peptone (BD), 7.6 g/l (NH_4_)_2_SO_4_, 15.0 g/l KH_2_PO_4_, 2.4 mM MgSO_4_.7H_2_0, 4.1 mM CaCI_2_.H_2_O, 3.7 mg/l CoCI_2_, 5 mg/l FeSO_4_.7H_2_O, 1.4 mg/l ZnSO_4_.7H_2_O, 1.6 mg/l MnSO_4_.7H_2_O, 0.1% Triton TX-100 (Fluka, St Louis, Missouri, USA) and 3% agar Noble (BD), pH 5.5, and plated on solid medium (composition of the medium was the same as that of the top agar except that β-glucan was omitted and the concentration of agar Noble was 1.8% (w/v)). After 4 days of cultivation at 28°C, the plates were rinsed with 0.9% NaCl, submerged in 0.1% Congo red (Merck, Darmstadt, Germany) in 1 M Tris-HCl (pH 9.5), and incubated for 30 min with shaking at 100 rpm. After the incubation, the plates were washed with 0.9% (w/v) NaCl, and the diameter of the colonies and the halo around them were measured. The size of the halo compared to the colony size was calculated and compared to the corresponding size of the parental strain QM9414.

### Construction of a deletion strain

The deletion cassette for the deletion of gene 77513 was constructed by Golden Gate cloning [51]. The construct contained the hygromycin resistance cassette (gpdA promoter, hygromycin resistance gene, trpC terminator) flanked by 1.523 kb and 1.024 kb fragments from the 5′ and 3′ sides of the ORF of 77513, respectively. The 5′-flanking region fragment was amplified by PCR with oligos 5′-GCGCGGTCTCCGGGTGGCGAGGTGGGAGAAGGGGA-3′ and 5′-GCGCGGTCTCGCATGGGAAGACGAGGTCGGTGTTG-3′. The 3′-flanking region was amplified by PCR with oligos 5′-GCGCGGTCTCCGAGAAAGCGGTCGGGGAAATGGCG-3′ and 5′-GCGCGGTCTCGGCGGTTGCGTGGGCGTTGCTCGAT-3′. The fragments of the marker cassette and the flanks were first ligated to a pBsV2 vector [52] and subsequently cloned to a modified pBluescript vector (lacking the *BsaI* site). The deletion cassette was digested from the vector with *PmeI* enzyme and transformed to *T. reesei* QM9414∆mus53 strain (QM9414 strain from which gene 58509 had been deleted) with high targeted integration frequency.

### Cultivation of *T. reesei* in shake flasks

*T. reesei* QM9414 and representative clones from transformations of each of the regulatory factor constructs were cultivated on medium containing 4% lactose (Fagron), 2% spent grain extract, 7.6 g/l (NH_4_)_2_SO_4_, 15.0 g/l KH_2_PO_4_, 2.4 mM MgSO_4_.7H_2_O, 4.1 mM CaCI_2_.H_2_O, 3.7 mg/l CoCI_2_, 5 mg/l FeSO_4_.7H_2_O, 1.4 mg/l ZnSO_4_.7H_2_O and 1.6 mg/l MnSO_4_.7H_2_O, pH adjusted to 5.2 with KOH. The culture medium was inoculated with 2 × 10^7^ spores per 200 ml of the medium, and grown at 28°C in conical flasks with shaking at 250 rpm for 10 days. The strains were cultivated in triplicate. Samples were collected after 3, 5, 7 and 9 or 10 days of cultivation. For RNA isolation, mycelium was collected by filtering the samples, and the mycelium was washed with equal volume of 0.7% NaCl, frozen immediately in liquid nitrogen and stored at −80°C. For measurement of the biomass dry weight, the filtered and washed mycelium samples were dried at 105°C to constant weight (24 h). Filtered culture media was used for enzymatic assays and for measuring pH.

### Enzyme assays

Cellulase activity against the MUL substrate, CBHI and EGI activity was determined by detecting the fluorescent hydrolysis product methylumbelliferone released from the substrate MUL (Sigma-Aldrich, Steinheim, Germany) as described in [53]. The combined activity of EGI and CBHI was measured by inhibiting β-glucosidase activity with glucose. EGI activity was measured by adding cellobiose to inhibit CBHI and glucose to inhibit β-glucosidase. CBHI activity was deduced by subtracting EGI activity from the combined CBHI and EGI activity. Endo-β-1.4-xylanase activity was assayed using 1.0% birch glucuronoxylan as a substrate [54]. The released reducing sugars were detected with 2-hydroxy-3,5-dinitrobenzoic acid. Pure xylose (Sigma-Aldrich) was used as a standard.

### Northern analysis

Total RNA was isolated from the mycelium samples using the Trizol™ Reagent (Gibco BRL, Carlsbad, California, USA), essentially according to manufacturer’s instructions. Northern blotting and hybridization on nitrocellulose filters (Hybond N, GE Healthcare) were carried out according to standard procedures [50]. Fragments of the genes to be analyzed were PCR amplified from the genomic DNA and used as probes in the Northern analysis. The signals in the northern blots were quantified using a phosphorimager (Typhoon imager, GE Healthcare), and the signals were normalized with those of actin.

### Quantitative PCR

Total RNA was isolated from the mycelial samples of three parallel cultivations collected at the cultivation time points 3 and 5 days using Trizol™ Reagent (Gibco BRL), essentially according to manufacturer’s instructions. Single stranded cDNA was synthesized using a QuantiTect Reverse Transcription Kit (Qiagen, Hilden, Germany), with 1.5 μg of total RNA as a template. The cDNA samples were diluted 1:10 to 1:50 and quantitative PCR reactions of two technical replicates were performed using a LightCycler 480 SYBR Green I Master Kit (Roche, Mannheim, Germany) according to the instructions of the manufacturer. The instrument used for quantitative PCR was Light Cycler 480 II and the results were analyzed with LightCycler 480 Software release 1.5.0. (version 1.5.0.39) using *sar1* signal for normalization. The primers used in the quantitative PCR are listed in Table 5.

**Table 5:** Primers for the quantitative PCR analysis

**Gene**	**5′ primer**	**3′ primer**
*cbh1*	GCGGATCCTCTTTCTCAG	ATGTTGGCGTAGTAATCATCC
*cbh2*	TCCTGGTTATTGAGCCTGAC	GCAACATTTGGAAGGTTCAG
*egl1*	GTCTACTACGAACTCGAC	GTAGTAGTCGTTGCTATACTG
*bgl1*	GCCTCCAAGATCAGCTATCC	ACCTCCTCACCGATGAACTG
*xyn1*	AAACTACCAAACTGGCGG	TTGATGGGAGCAGAAGATCC
*xyn2*	CGGCTACTTCTACTCGTACTG	TTGATGACCTTGTTCTTGGTG
*xyn3*	TACAAGGGCAAGATTCGTG	ACTGGCTTCCAATACCGT
*axe1*	TAAAGCAGCAATCTTCATGG	GCAGTAAGACTTGATCTTGG
*bxl1*	GTCACTCTTCCAAGCTCAG	ATCGTTACCTCTTCTCCCA
*xyr1*	GAGTATCAGCGCAACTTTAGCA	CATCGGTATAGTGCAAGAAGCTC
*sar1*	TCTCCACCCTACTTCTGAG	CTTGTTGCCCAGGATGAC

## Abbreviations

CAZy: carbohydrate active enzyme; CBHI: cellobiohydrolase 1; EGI: endoglucanase 1; MUL: 4-methylumbelliferyl-β-D-lactoside; ORF: open reading frame; PCR: polymerase chain reaction.

## Competing interests

The authors declare that they have no competing interests.

## Authors’ contributions

MH carried out cloning of the genes, participated in the construction and cultivation of the recombinant strains, enzymatic activity measurements and quantitative PCR analysis, carried out fungal cultivations and microarray detection of the expression signals for the second cultivation set, and drafted the manuscript. MJV carried out the Southern and northern hybridizations. AWP participated in the cloning of the genes, construction and cultivation of the recombinant strains and enzymatic activity measurements. NA carried out quantitative PCR analysis of transcript levels. MA analyzed the transcriptome data from the chemostat cultivations. MV participated in the data analysis and constructed the deletion strain. MP and MS conceived of the study, and participated in its design and coordination. TMP participated in the design and coordination of the study, carried out microarray data analysis including the selection of the candidate genes, and helped to draft the manuscript. All authors read and approved the final manuscript.

## Supplementary Material

Additional file 1 **Transcriptional profiling data of the putative regulatory genes.** Gene identifiers are as in *T. reesei* database version 2.0. Functional Interpro domain identifiers are as in InterPro database. Fold changes (log2 scale compared to uninduced control culture at a corresponding time point), signal intensities (log2 scale) and significance test (R package limma, *P* <0.01, log2 fold change >0.4) are shown for the genes. 1 indicates induction and −1 repression. The intensity of the red color and blue color indicates the strength of positive and negative fold changes, respectively. Color scales of yellow, red and green indicate different intensities of signals, red represents the strongest signals and green the weakest signals. AV1, 1% Avicel cellulose; AV0.75, 0.75% Avicel cellulose; BE, enzymatically hydrolyzed steam-exploded bagasse; BO, ground bagasse; BS, steam-exploded bagasse; SO, sophorose; SP, steam-exploded spruce; WH, steam-exploded wheat straw; XB, birch xylan; XO, oat spelt xylan. Click here for file

Additional file 2 **Production of total proteins and cellulase and xylanase activity by the recombinant strains at different time points of the cultivation.** Results are shown for each strain volumetrically (nkat/l) and per biomass dry weight (nkat/g). The values are means of three biological replicates. Error bars show the standard error of the mean. BGL, β-glucosidase activity; CBHI, cellobiohydrolase activity; EGI, endoglucanase activity; MUL, total cellulase activity measured against the substrate 4-methylumbelliferyl-β-D-lactoside; XYN, xylanase activity. Click here for file

Additional file 3 **Results of Southern hybridizations.** Position of the molecular weight size marker is shown as kb on the left. The restriction enzymes used for the digestion in the analysis are indicated by the letters: A, *NcoI* + *BstXI*; B, *BglII*; C, *SpeI* + *BclI*; D, *ClaI* + *BamHI*; E, *SacI*; F, *NaeI*; G, *ClaI* + *XbaI*; H, *SnaBI* + *XbaI*; I, *StuI*; J, *SacI*; K, *StuI*; L, *XmnI*; M, *BstEII*; N, *SspI*; O, *StuI*; P, *SspI*; Q, *StuI*. For Del77513 strain two different probes were used: hygromycin selection marker (hph) open reading frame (N and O) and fragment of the gene 77513 open reading frame (P and Q). Click here for file

Additional file 4 **Results of Northern hybridizations.** Northern blot analysis of the expression of the candidate regulatory genes in the recombinant strains. **(A)** mRNA signals of genes 123668, 80291, 74765, 122523, 66966 and 64608 in cultures of the strains harboring the corresponding overexpression cassettes pMH18, pMH20, pMH25, pMH29, pMH35 and pMH36, respectively, are shown on the top. The mobility of the transcript encoded by the overexpression construct is indicated by an arrow in the blot. Samples collected after 3 days of cultivation (two biological replicates) were analyzed. The northern hybridization signal of actin and staining of total RNA with the SYBR Green II in the same gels are shown below each of the northern blots, as indicated. **(B)** mRNA signals of gene 77513 in cultures of overexpression strains pMH15 and pMH15(S), and in the Del77513 strain. Samples collected after 3 and 5 days of cultivation (two biological replicates) were analyzed. The northern hybridization signal of actin and staining of total RNA with the SYBR Green II in the same gel are shown below, as indicated. **(C)** Signal fold change of the northern signals in the recombinant strain versus the control strain. Signal intensities were normalized using the actin signal. Click here for file

Additional file 5 **pMS204 vector with hygromycin resistance gene and gateway cloning cassette under gpdA promoter and trpC terminator.** AmpR, ampicillin resistance gene; attR1/attR2, att sites for recombination; ccdB, ccdB gene for negative selection; CmR, chloramphenicol resistance gene; hph, hygromycin resistance gene; MCS, multiple cloning site; ORI, origin of replication. Click here for file
