# Supplementary material for: Screening of candidate regulators for cellulase and hemicellulase production in Trichoderma reesei and identification of a factor essential for cellulase production
Source: Biotechnol Biofuels. 2014 Jan 28;7:14. doi: 10.1186/1754-6834-7-14 (PMC3922861; doi:10.1186/1754-6834-7-14)
Supplement: Additional file 2 — Production of total proteins and cellulase and xylanase activity by the recombinant strains at different time points of the cultivation. Results are shown for each strain volumetrically (nkat/l) and per biomass dry weight (nkat/g). The values are means of three biological replicates. Error bars show the standard error of the mean. BGL, β-glucosidase activity; CBHI, cellobiohydrolase activity; EGI, endoglucanase activity; MUL, total cellulase activity measured against the substrate 4-methylumbelliferyl-β-D-lactoside; XYN, xylanase activity. [file 1754-6834-7-14-S2.pdf]

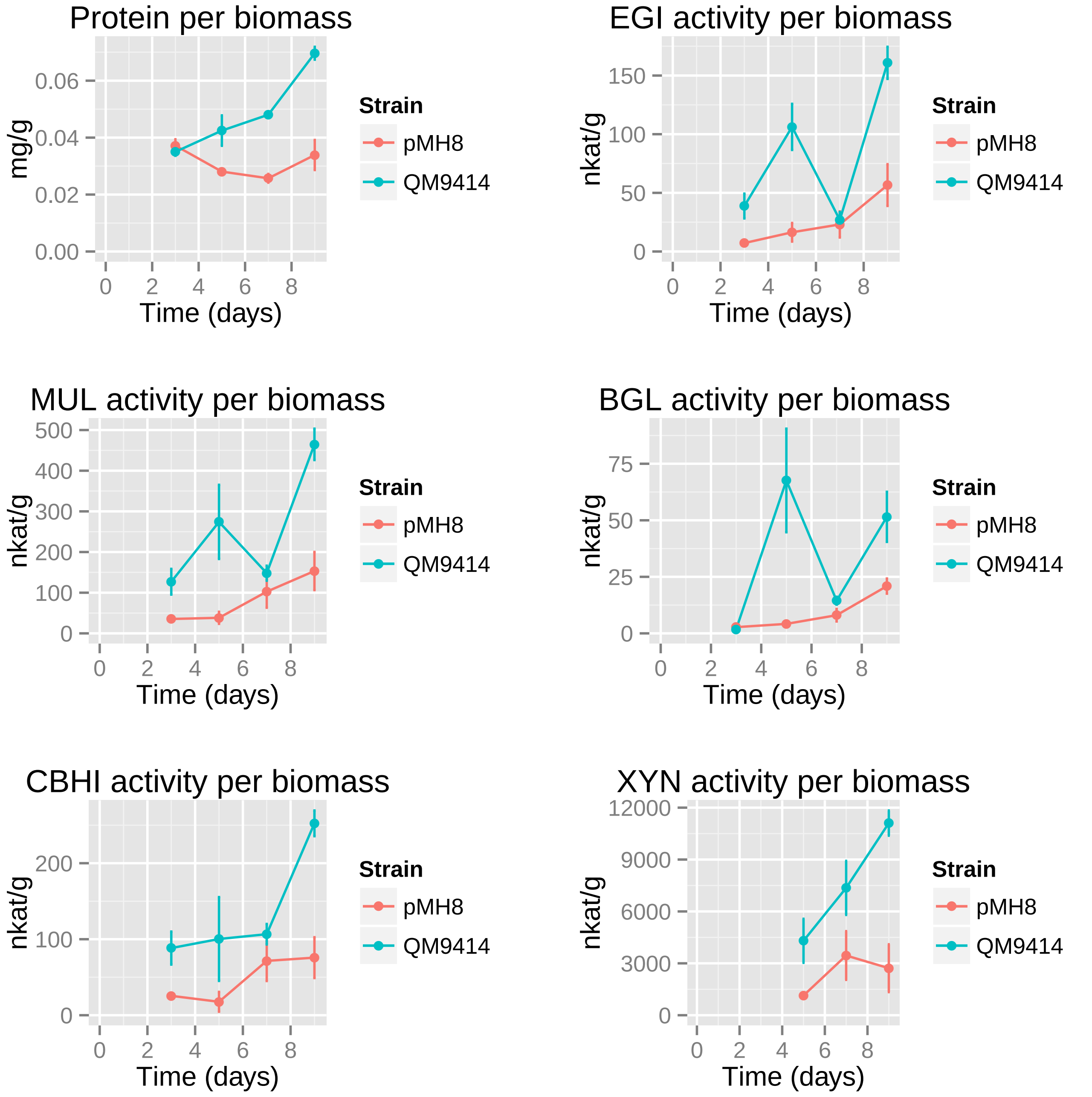

Protein per biomass
mg/g
0.00
0.02
0.04
0.06
Time (days)
0
2
4
6
8
Strain
pMH8
QM9414
EGI activity per biomass
nkat/g
0
50
100
150
Time (days)
Strain
pMH8
QM9414
MUL activity per biomass
nkat/g
0
100
200
300
400
500
Time (days)
Strain
pMH8
QM9414
BGL activity per biomass
nkat/g
0
25
50
75
Time (days)
Strain
pMH8
QM9414
CBHI activity per biomass
nkat/g
0
100
200
Time (days)
Strain
pMH8
QM9414
XYN activity per biomass
nkat/g
0
3000
6000
9000
12000
Time (days)
Strain
pMH8
QM9414

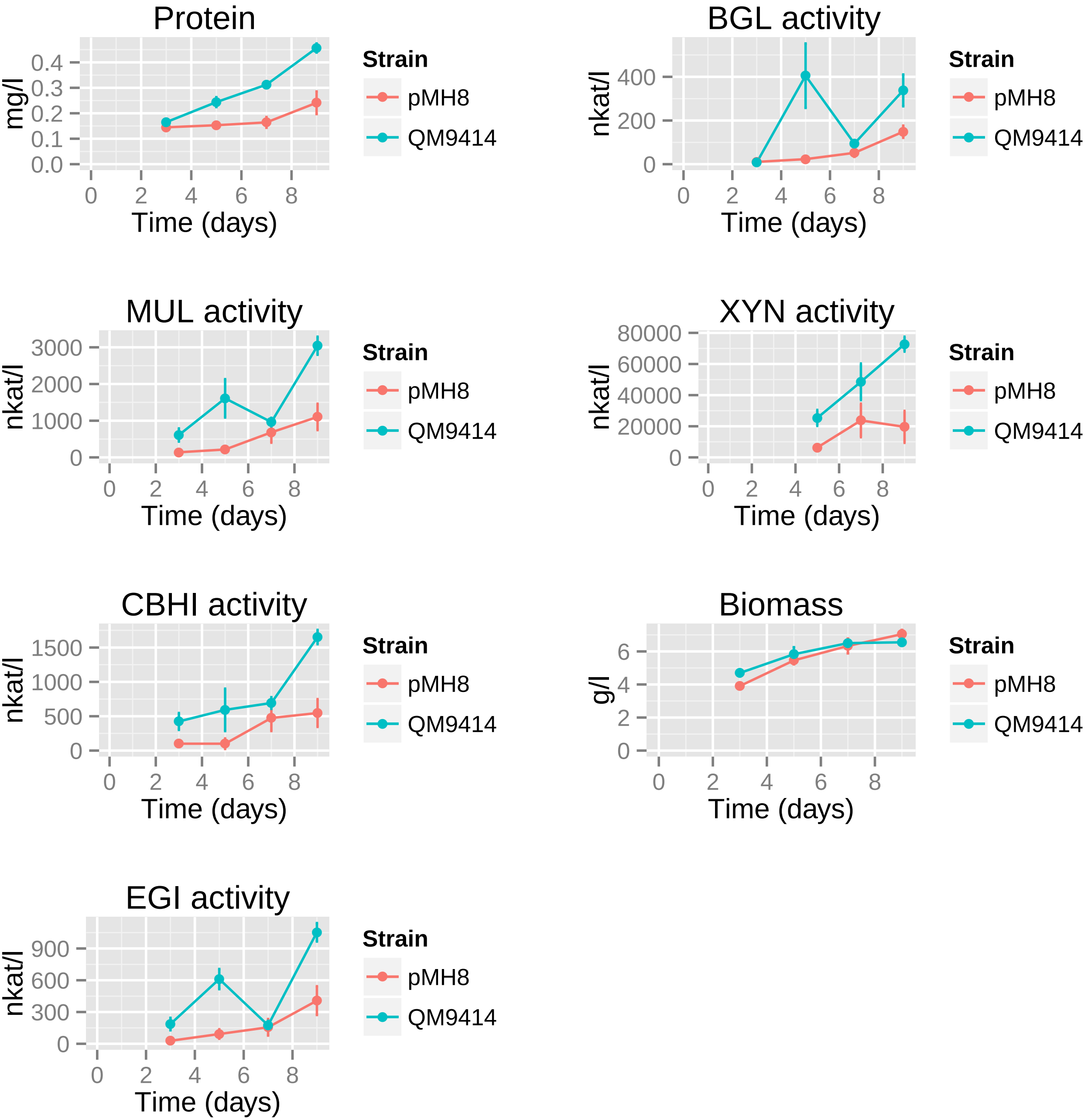
Protein
mg/l
0.0
0.1
0.2
0.3
0.4
0
2
4
6
8
Time (days)
Strain
pMH8
QM9414
BGL activity
nkat/l
0
200
400
0
2
4
6
8
Time (days)
Strain
pMH8
QM9414
MUL activity
nkat/l
0
1000
2000
3000
0
2
4
6
8
Time (days)
Strain
pMH8
QM9414
XYN activity
nkat/l
0
20000
40000
60000
80000
0
2
4
6
8
Time (days)
Strain
pMH8
QM9414
CBHI activity
nkat/l
0
500
1000
1500
0
2
4
6
8
Time (days)
Strain
pMH8
QM9414
Biomass
g/l
0
2
4
6
0
2
4
6
8
Time (days)
Strain
pMH8
QM9414
EGI activity
nkat/l
0
300
600
900
0
2
4
6
8
Time (days)
Strain
pMH8
QM9414

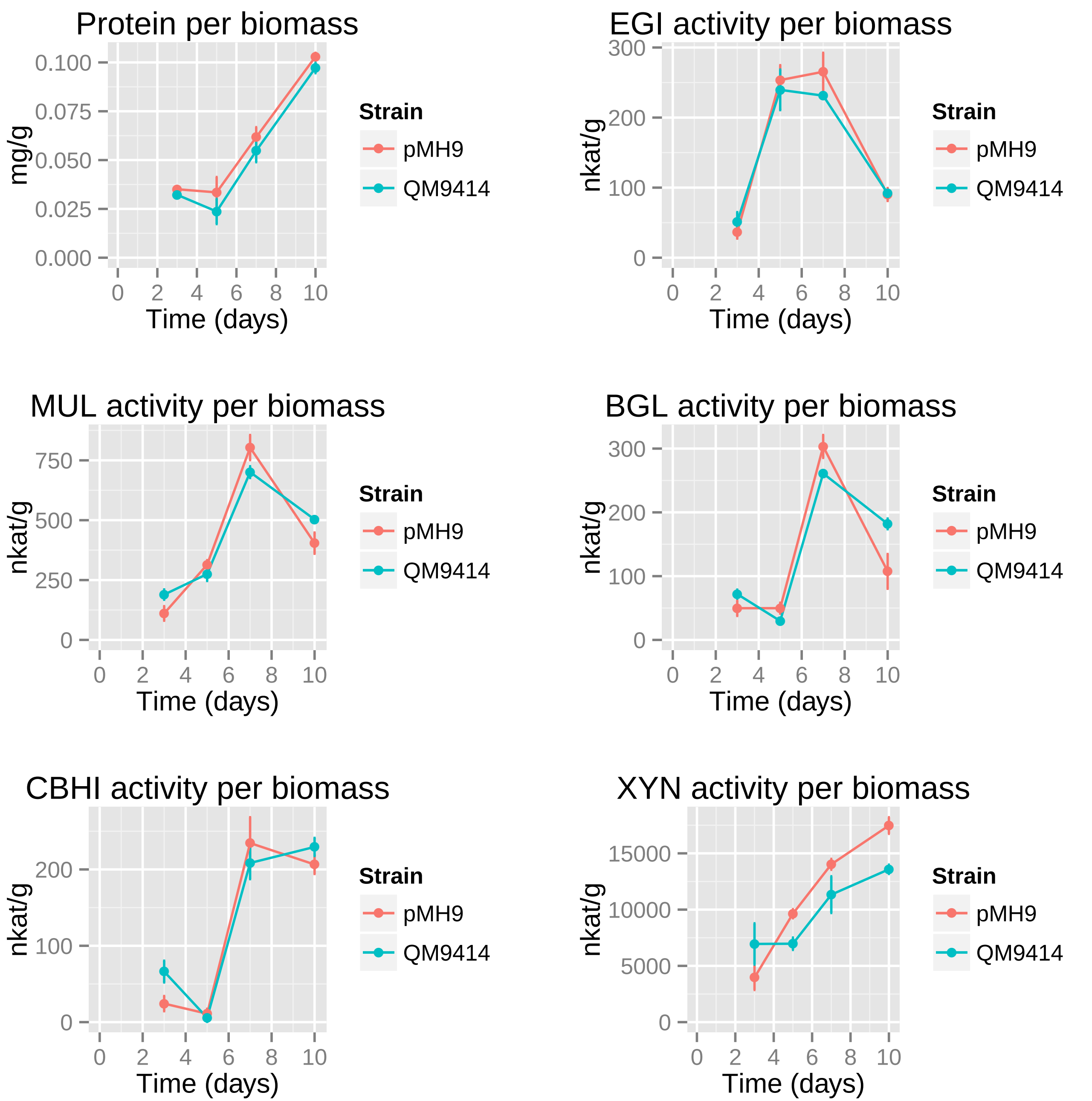
Protein per biomass
mg/g
0.100
0.075
0.050
0.025
0.000
0
2
4
6
8
10
Time (days)
Strain
pMH9
QM9414
EGI activity per biomass
nkat/g
300
200
100
0
MUL activity per biomass
750
500
250
BGL activity per biomass
CBHI activity per biomass
XYN activity per biomass
15000
10000
5000

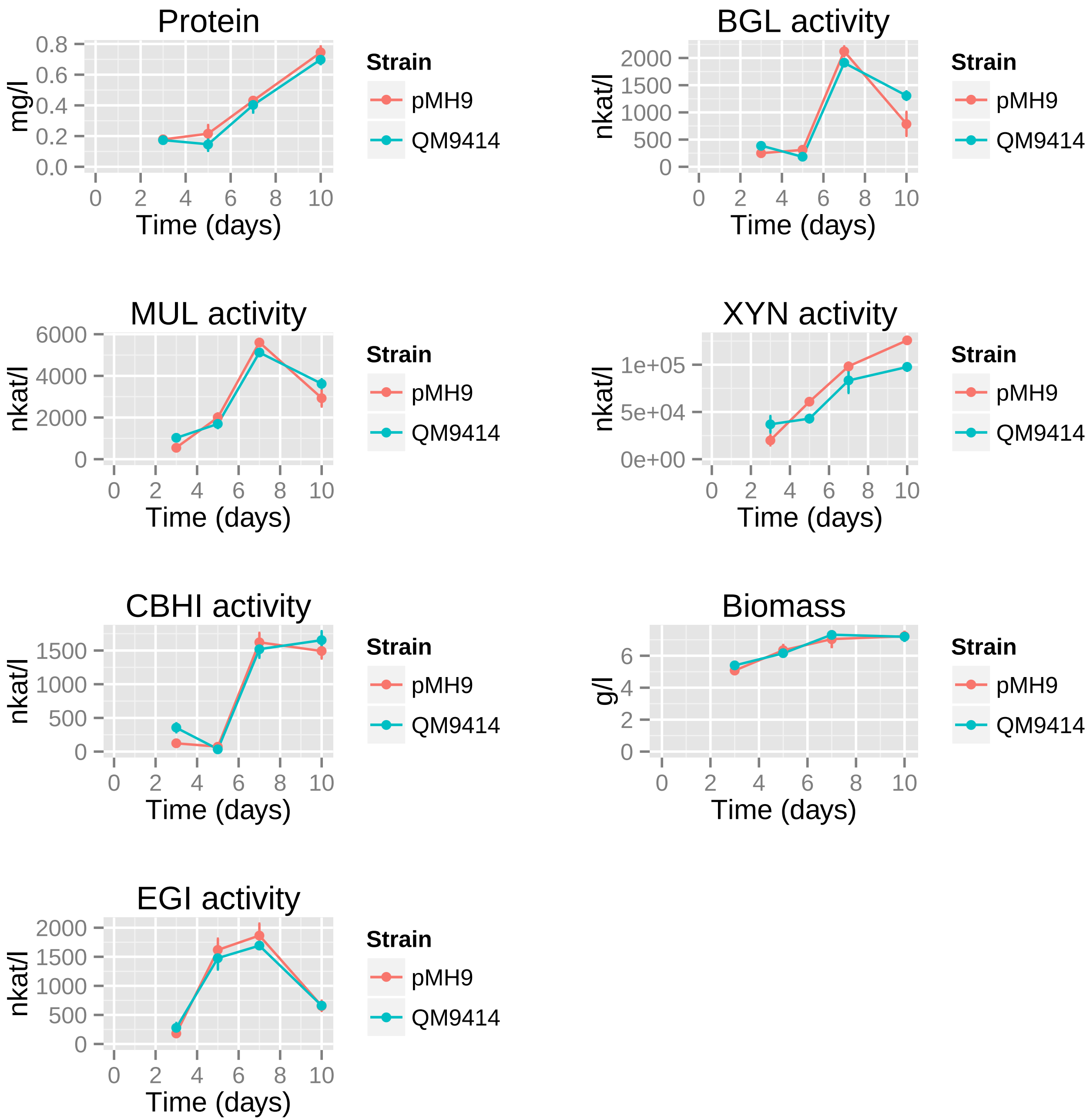
Protein
mg/l
0.0
0.2
0.4
0.6
0.8
0 2 4 6 8 10
Time (days)
Strain
pMH9
QM9414
BGL activity
nkat/l
0
500
1000
1500
2000
0 2 4 6 8 10
Time (days)
Strain
pMH9
QM9414
MUL activity
nkat/l
0
2000
4000
6000
0 2 4 6 8 10
Time (days)
Strain
pMH9
QM9414
XYN activity
nkat/l
0e+00
5e+04
1e+05
0 2 4 6 8 10
Time (days)
Strain
pMH9
QM9414
CBHI activity
nkat/l
0
500
1000
1500
0 2 4 6 8 10
Time (days)
Strain
pMH9
QM9414
Biomass
g/l
0
2
4
6
0 2 4 6 8 10
Time (days)
Strain
pMH9
QM9414
EGI activity
nkat/l
0
500
1000
1500
2000
0 2 4 6 8 10
Time (days)
Strain
pMH9
QM9414

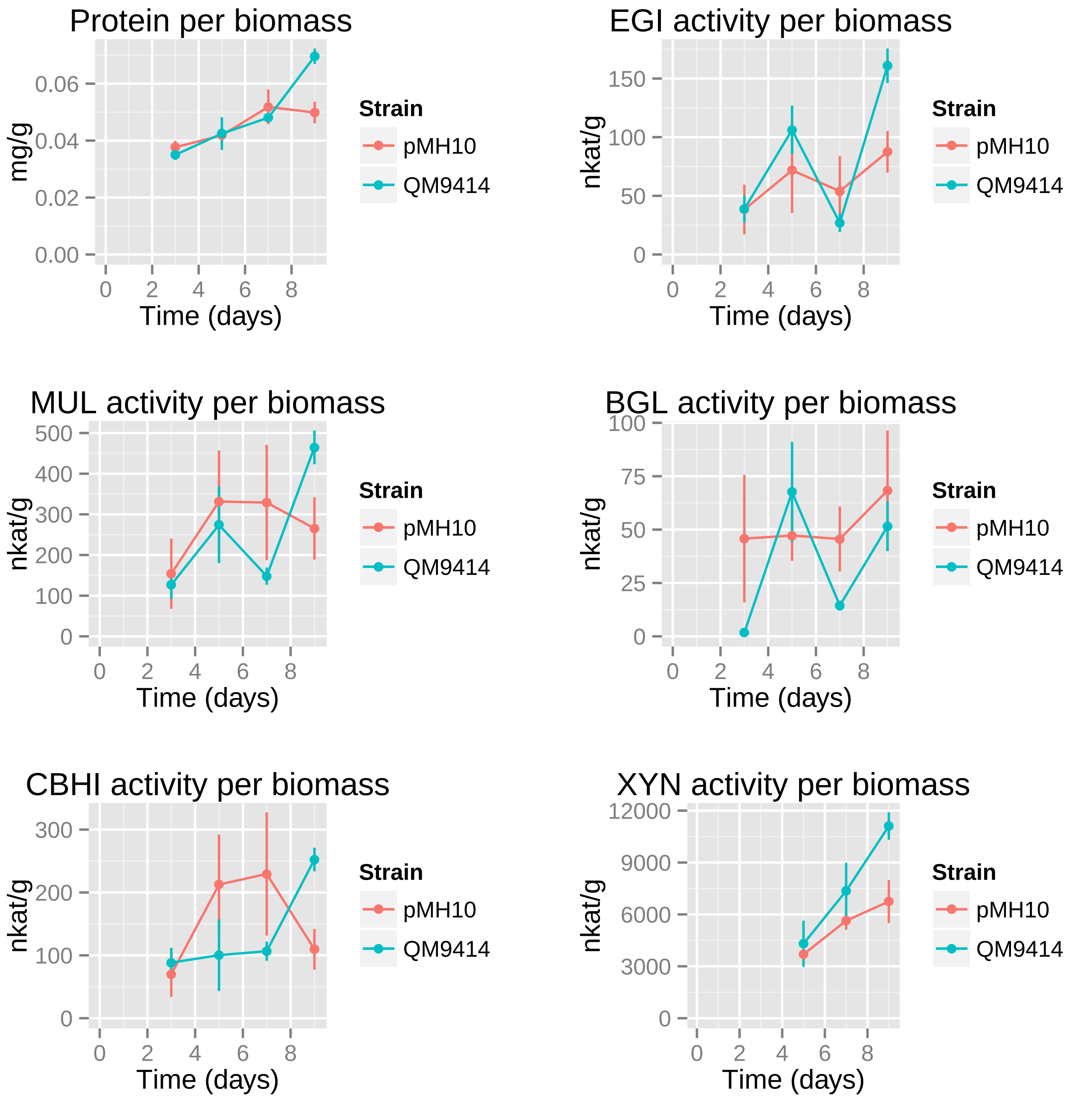
Protein per biomass
mg/g
0.00
0.02
0.04
0.06
0
2
4
6
8
Time (days)
Strain
pMH10
QM9414
EGI activity per biomass
nkat/g
0
50
100
150
0
2
4
6
8
Time (days)
Strain
pMH10
QM9414
MUL activity per biomass
nkat/g
0
100
200
300
400
500
0
2
4
6
8
Time (days)
Strain
pMH10
QM9414
BGL activity per biomass
nkat/g
0
25
50
75
100
0
2
4
6
8
Time (days)
Strain
pMH10
QM9414
CBHI activity per biomass
nkat/g
0
100
200
300
0
2
4
6
8
Time (days)
Strain
pMH10
QM9414
XYN activity per biomass
nkat/g
0
3000
6000
9000
12000
0
2
4
6
8
Time (days)
Strain
pMH10
QM9414

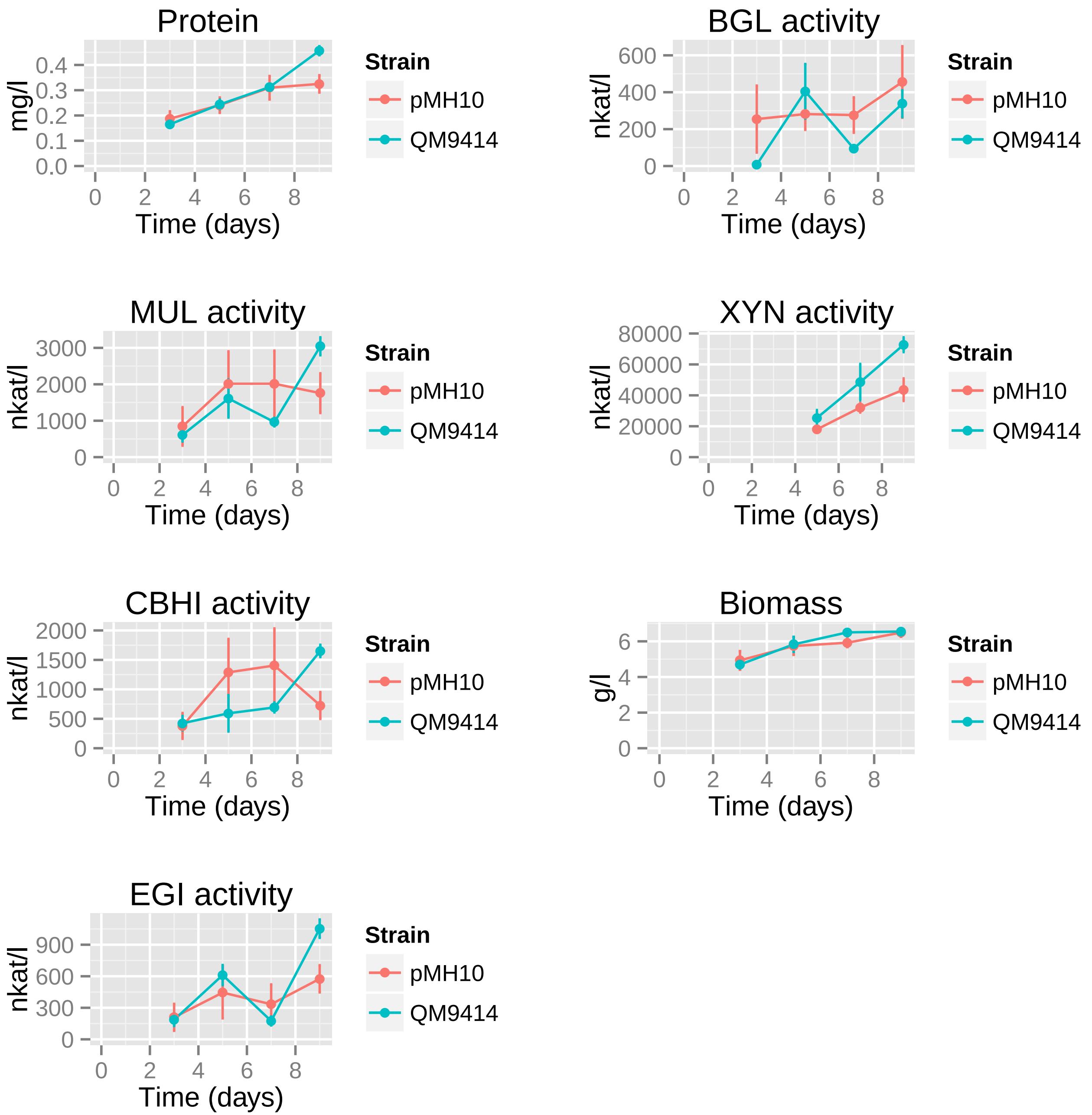

Protein
mg/l
Time (days)
Strain
pMH10
QM9414
BGL activity
nkat/l
Time (days)
Strain
pMH10
QM9414
MUL activity
nkat/l
Time (days)
Strain
pMH10
QM9414
XYN activity
nkat/l
Time (days)
Strain
pMH10
QM9414
CBHI activity
nkat/l
Time (days)
Strain
pMH10
QM9414
Biomass
g/l
Time (days)
Strain
pMH10
QM9414
EGI activity
nkat/l
Time (days)
Strain
pMH10
QM9414

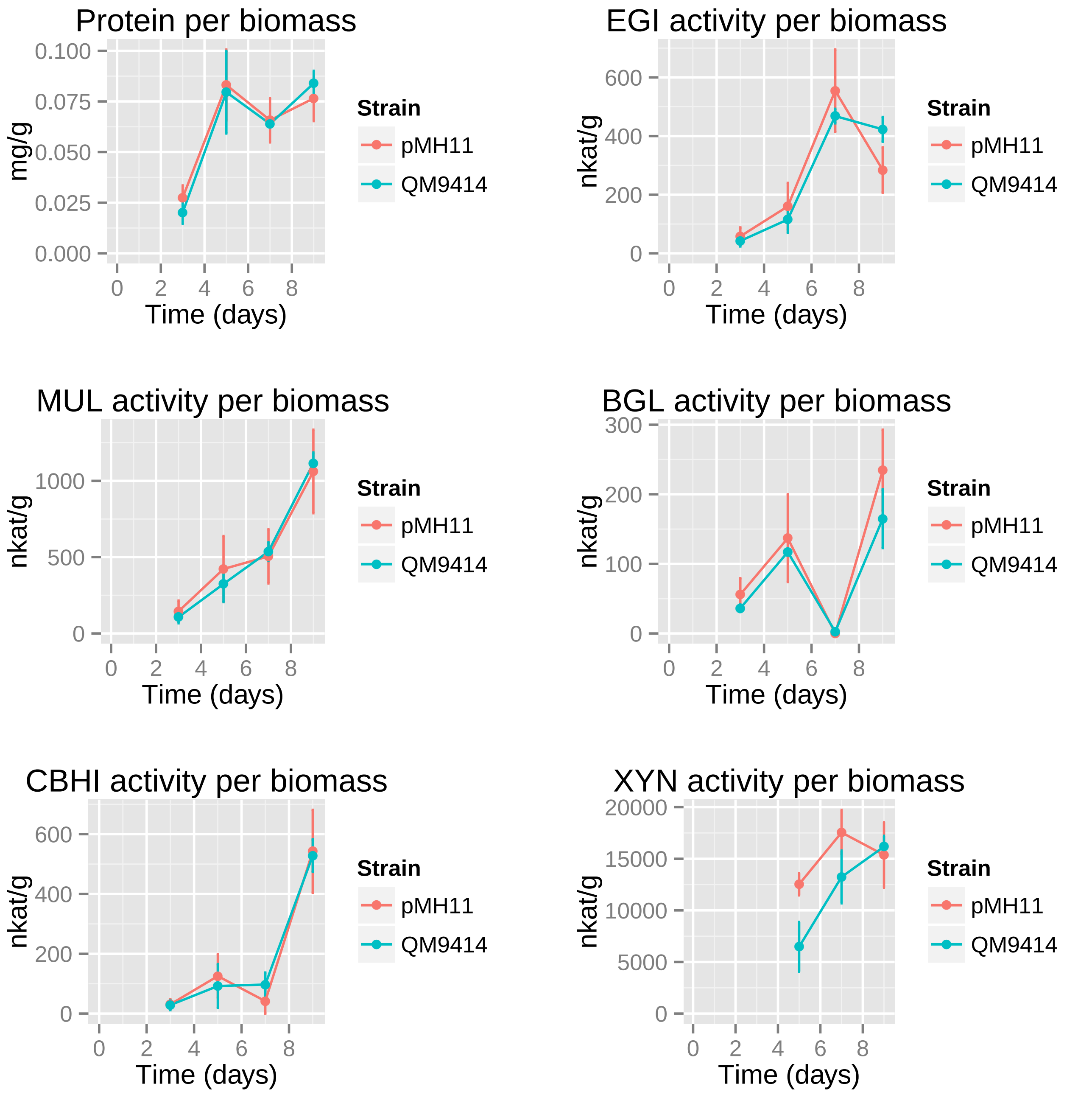
Protein per biomass
mg/g
0.100
0.075
0.050
0.025
0.000
0
2
4
6
8
Time (days)
Strain
pMH11
QM9414
EGI activity per biomass
nkat/g
600
400
200
0
Time (days)
Strain
pMH11
QM9414
MUL activity per biomass
nkat/g
1000
500
0
Time (days)
Strain
pMH11
QM9414
BGL activity per biomass
nkat/g
300
200
100
0
Time (days)
Strain
pMH11
QM9414
CBHI activity per biomass
nkat/g
600
400
200
0
Time (days)
Strain
pMH11
QM9414
XYN activity per biomass
nkat/g
20000
15000
10000
5000
0
Time (days)
Strain
pMH11
QM9414

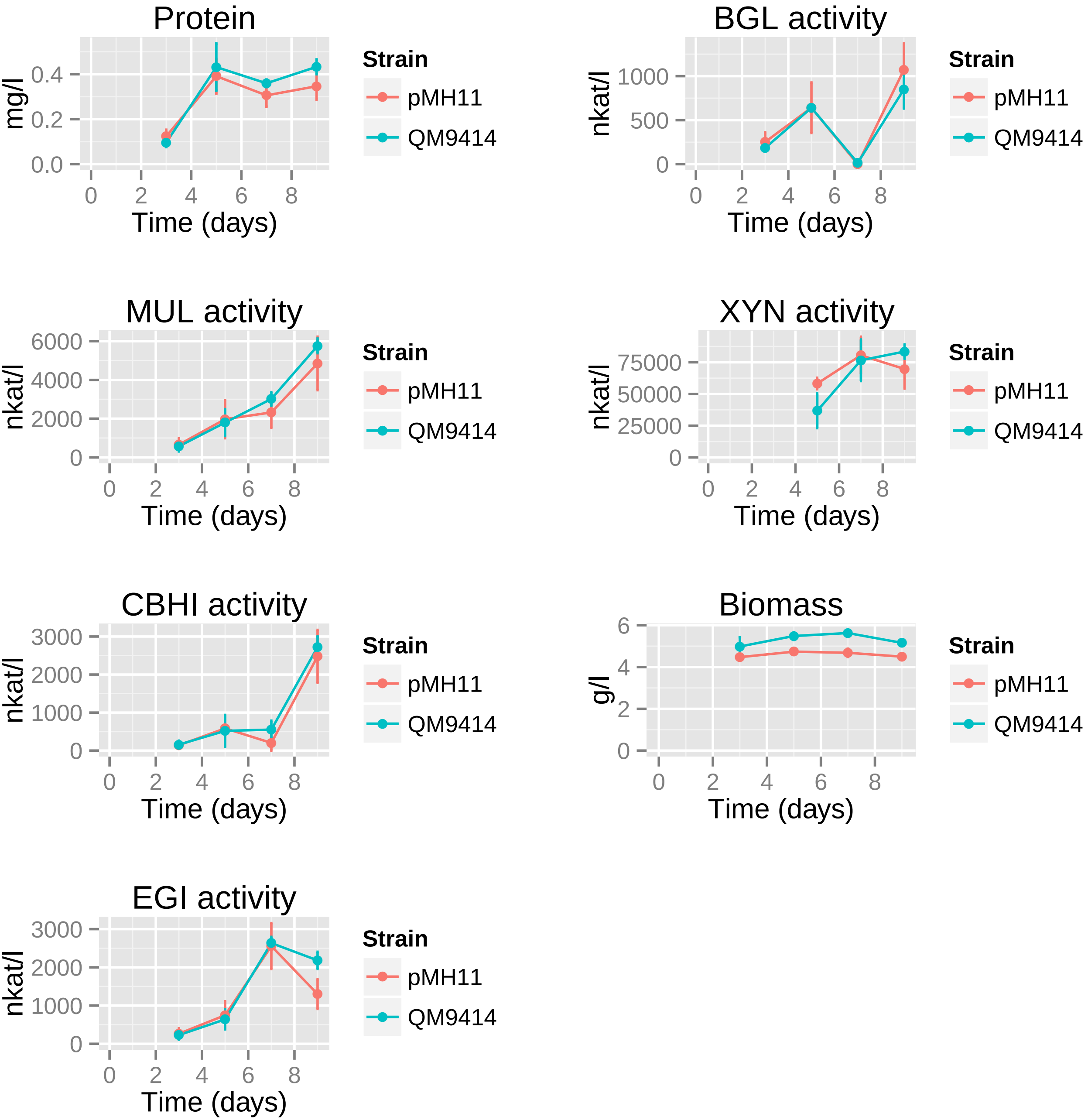
Protein
mg/l
Time (days)
BGL activity
nkat/l
MUL activity
XYN activity
CBHI activity
Biomass
g/l
EGI activity
Strain
pMH11
QM9414

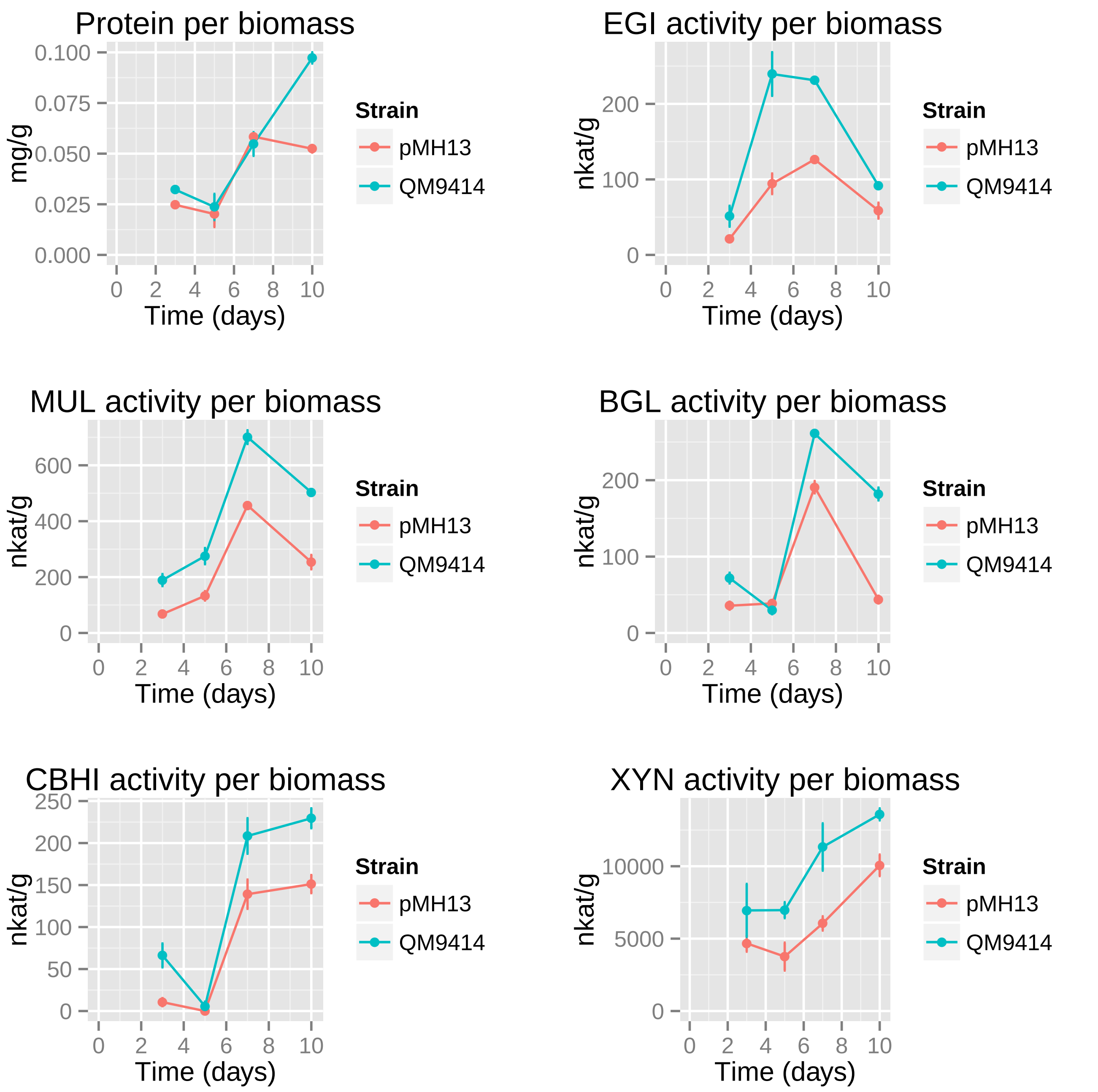
Protein per biomass
mg/g
0.100
0.075
0.050
0.025
0.000
0 2 4 6 8 10
Time (days)
Strain
pMH13
QM9414
EGI activity per biomass
nkat/g
200
100
0
0 2 4 6 8 10
Time (days)
Strain
pMH13
QM9414
MUL activity per biomass
nkat/g
600
400
200
0
0 2 4 6 8 10
Time (days)
Strain
pMH13
QM9414
BGL activity per biomass
nkat/g
200
100
0
0 2 4 6 8 10
Time (days)
Strain
pMH13
QM9414
CBHI activity per biomass
nkat/g
250
200
150
100
50
0
0 2 4 6 8 10
Time (days)
Strain
pMH13
QM9414
XYN activity per biomass
nkat/g
10000
5000
0
0 2 4 6 8 10
Time (days)
Strain
pMH13
QM9414

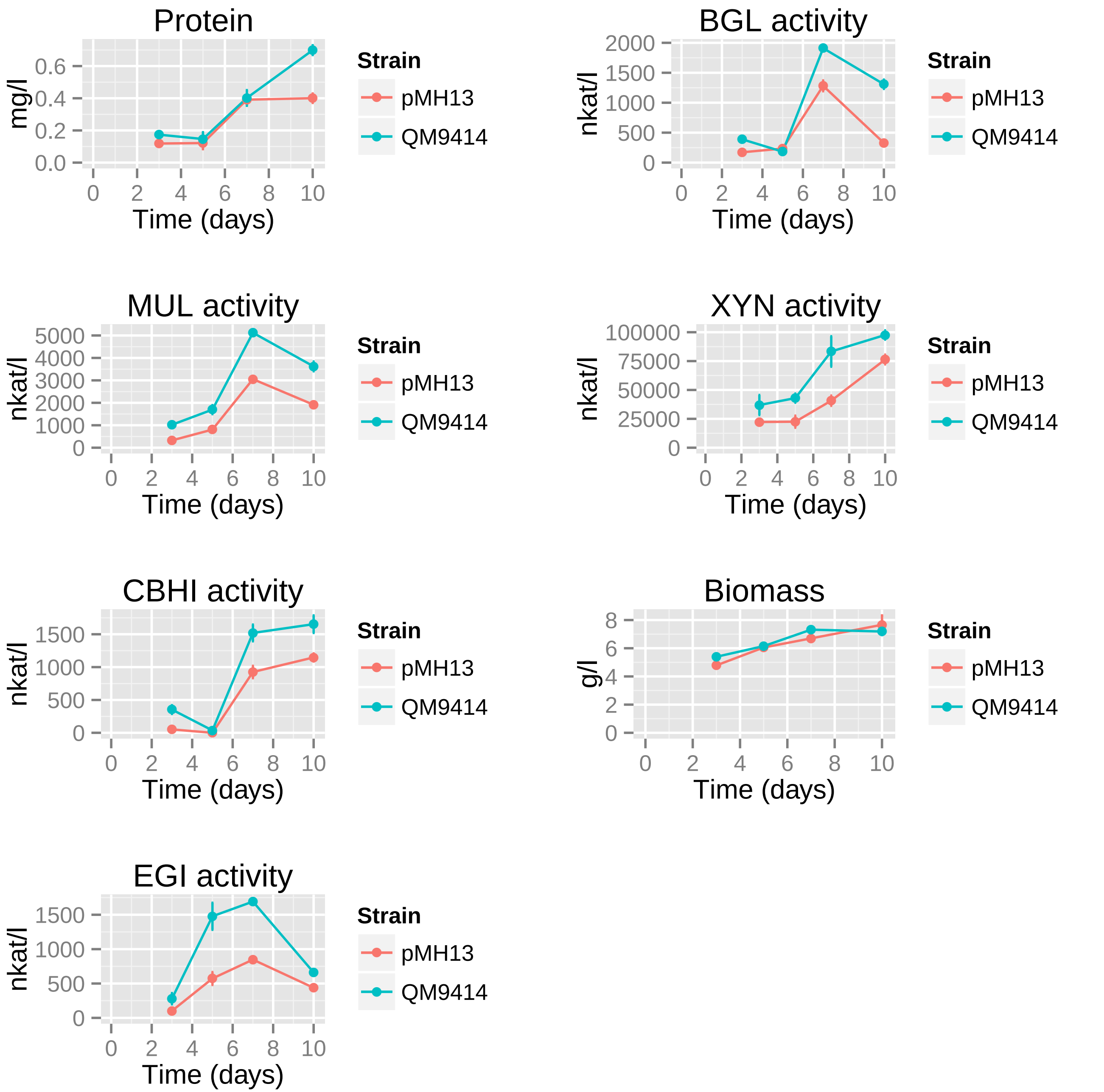

Protein
mg/l
0.0
0.2
0.4
0.6
Time (days)
0 2 4 6 8 10
Strain
pMH13
QM9414
BGL activity
nkat/l
0
500
1000
1500
2000
Time (days)
0 2 4 6 8 10
Strain
pMH13
QM9414
MUL activity
nkat/l
0
1000
2000
3000
4000
5000
Time (days)
0 2 4 6 8 10
Strain
pMH13
QM9414
XYN activity
nkat/l
0
25000
50000
75000
100000
Time (days)
0 2 4 6 8 10
Strain
pMH13
QM9414
CBHI activity
nkat/l
0
500
1000
1500
Time (days)
0 2 4 6 8 10
Strain
pMH13
QM9414
Biomass
g/l
0
2
4
6
8
Time (days)
0 2 4 6 8 10
Strain
pMH13
QM9414
EGI activity
nkat/l
0
500
1000
1500
Time (days)
0 2 4 6 8 10
Strain
pMH13
QM9414

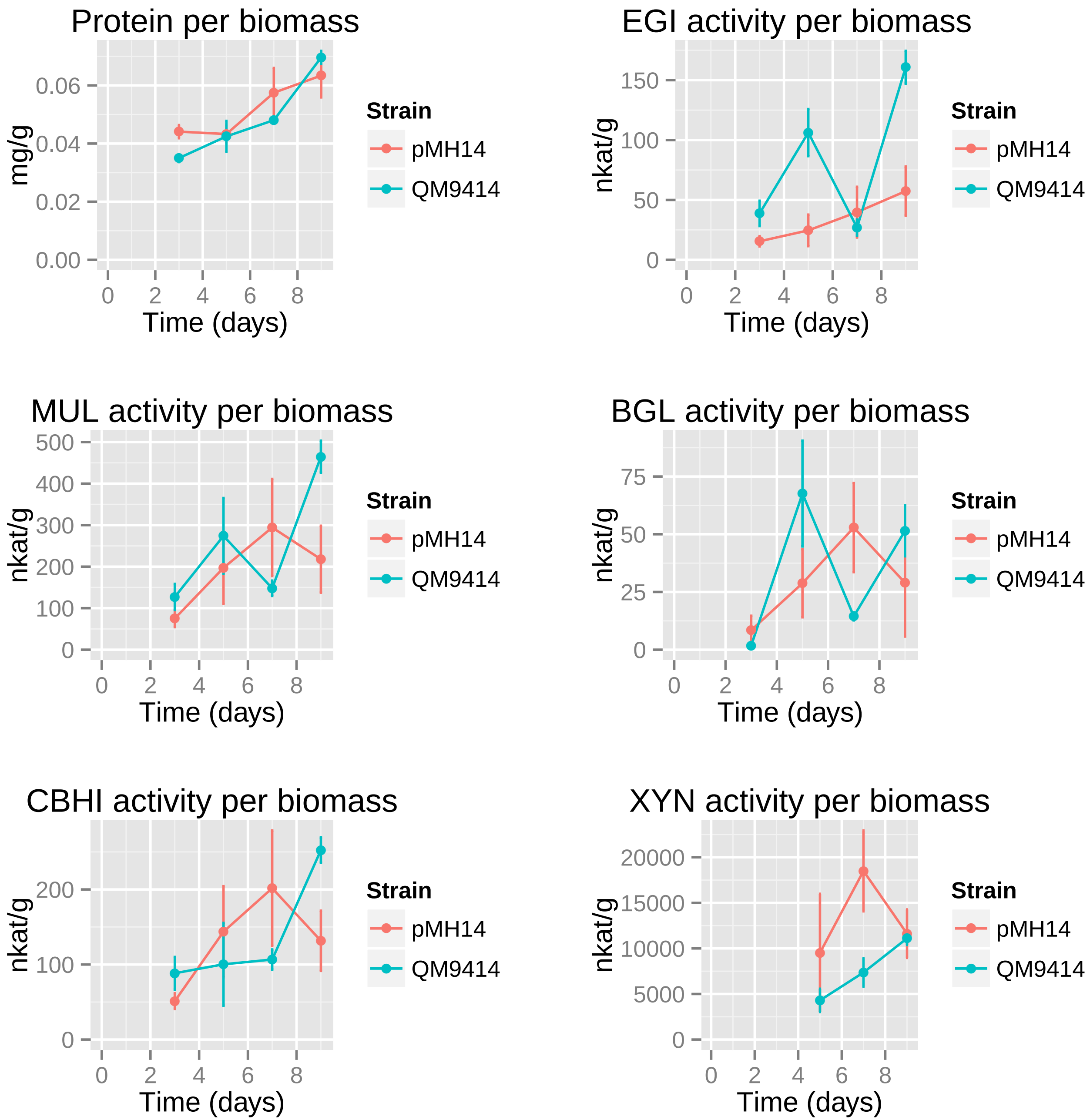
Protein per biomass
mg/g
0.00
0.02
0.04
0.06
0
2
4
6
8
Time (days)
Strain
pMH14
QM9414
EGI activity per biomass
nkat/g
0
50
100
150
0
2
4
6
8
Time (days)
Strain
pMH14
QM9414
MUL activity per biomass
nkat/g
0
100
200
300
400
500
0
2
4
6
8
Time (days)
Strain
pMH14
QM9414
BGL activity per biomass
nkat/g
0
25
50
75
0
2
4
6
8
Time (days)
Strain
pMH14
QM9414
CBHI activity per biomass
nkat/g
0
100
200
0
2
4
6
8
Time (days)
Strain
pMH14
QM9414
XYN activity per biomass
nkat/g
0
5000
10000
15000
20000
0
2
4
6
8
Time (days)
Strain
pMH14
QM9414

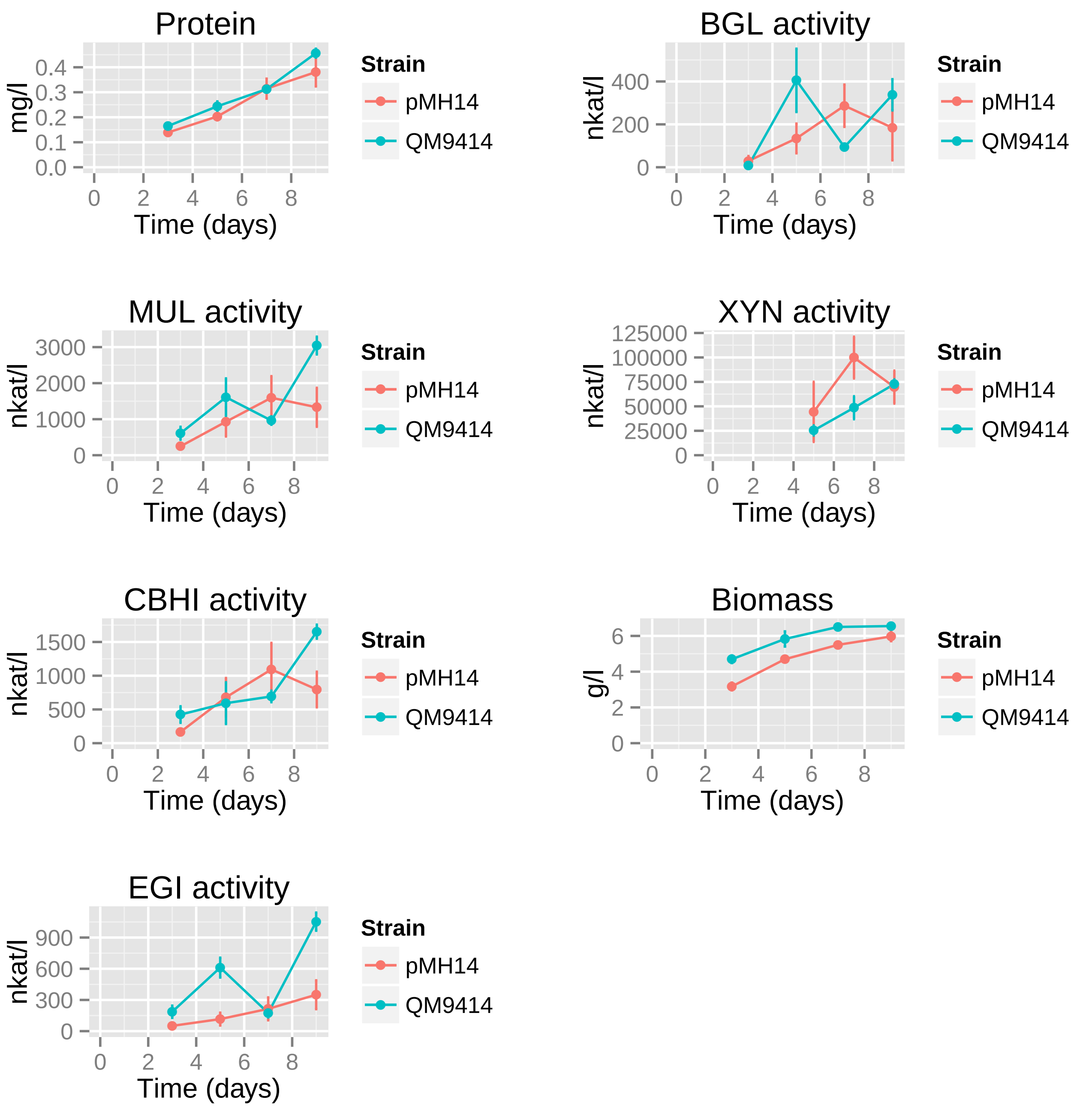
Protein
mg/l
Time (days)
BGL activity
nkat/l
MUL activity
XYN activity
CBHI activity
Biomass
g/l
EGI activity
Strain
pMH14
QM9414

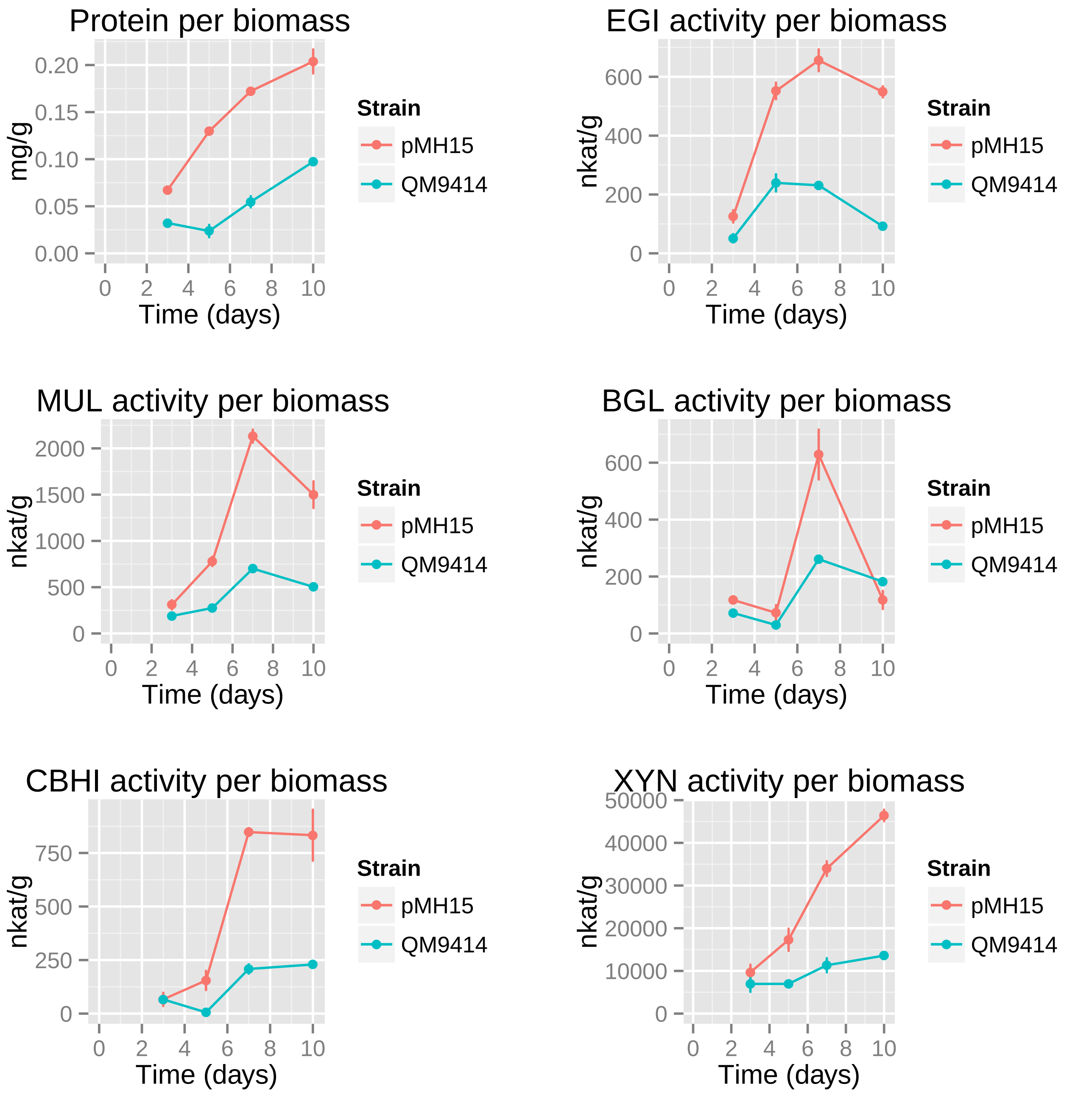

Protein per biomass
mg/g
0.00
0.05
0.10
0.15
0.20
Time (days)
EGI activity per biomass
nkat/g
0
200
400
600
MUL activity per biomass
0
500
1000
1500
2000
BGL activity per biomass
CBHI activity per biomass
250
750
XYN activity per biomass
10000
20000
30000
40000
50000
0
2
4
6
8
10
Strain
pMH15
QM9414

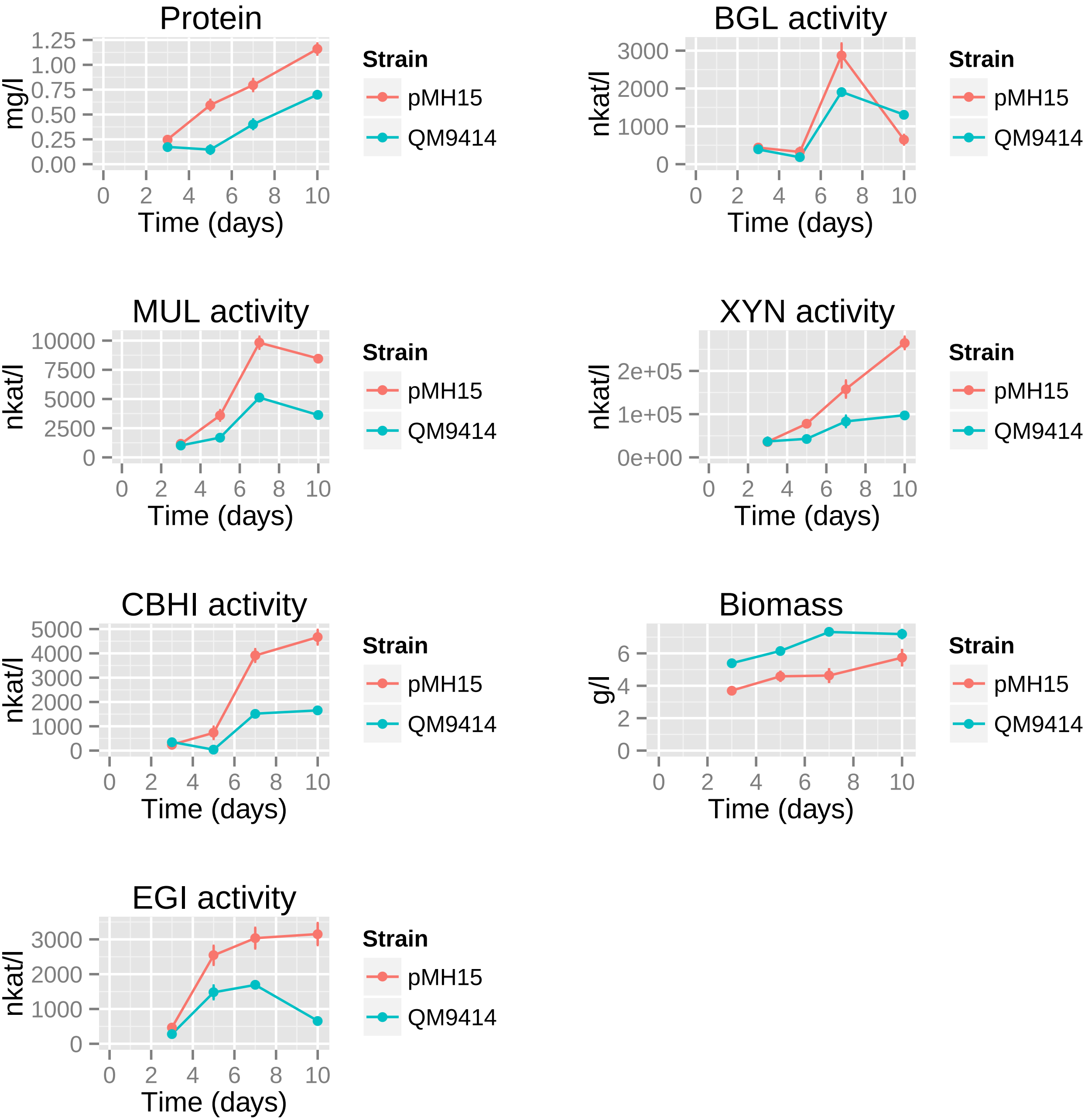
Protein
mg/l
1.25
1.00
0.75
0.50
0.25
0.00
0 2 4 6 8 10
Time (days)
Strain
pMH15
QM9414
BGL activity
nkat/l
3000
2000
1000
0
0 2 4 6 8 10
Time (days)
Strain
pMH15
QM9414
MUL activity
nkat/l
10000
7500
5000
2500
0
0 2 4 6 8 10
Time (days)
Strain
pMH15
QM9414
XYN activity
nkat/l
2e+05
1e+05
0e+00
0 2 4 6 8 10
Time (days)
Strain
pMH15
QM9414
CBHI activity
nkat/l
5000
4000
3000
2000
1000
0
0 2 4 6 8 10
Time (days)
Strain
pMH15
QM9414
Biomass
g/l
6
4
2
0
0 2 4 6 8 10
Time (days)
Strain
pMH15
QM9414
EGI activity
nkat/l
3000
2000
1000
0
0 2 4 6 8 10
Time (days)
Strain
pMH15
QM9414

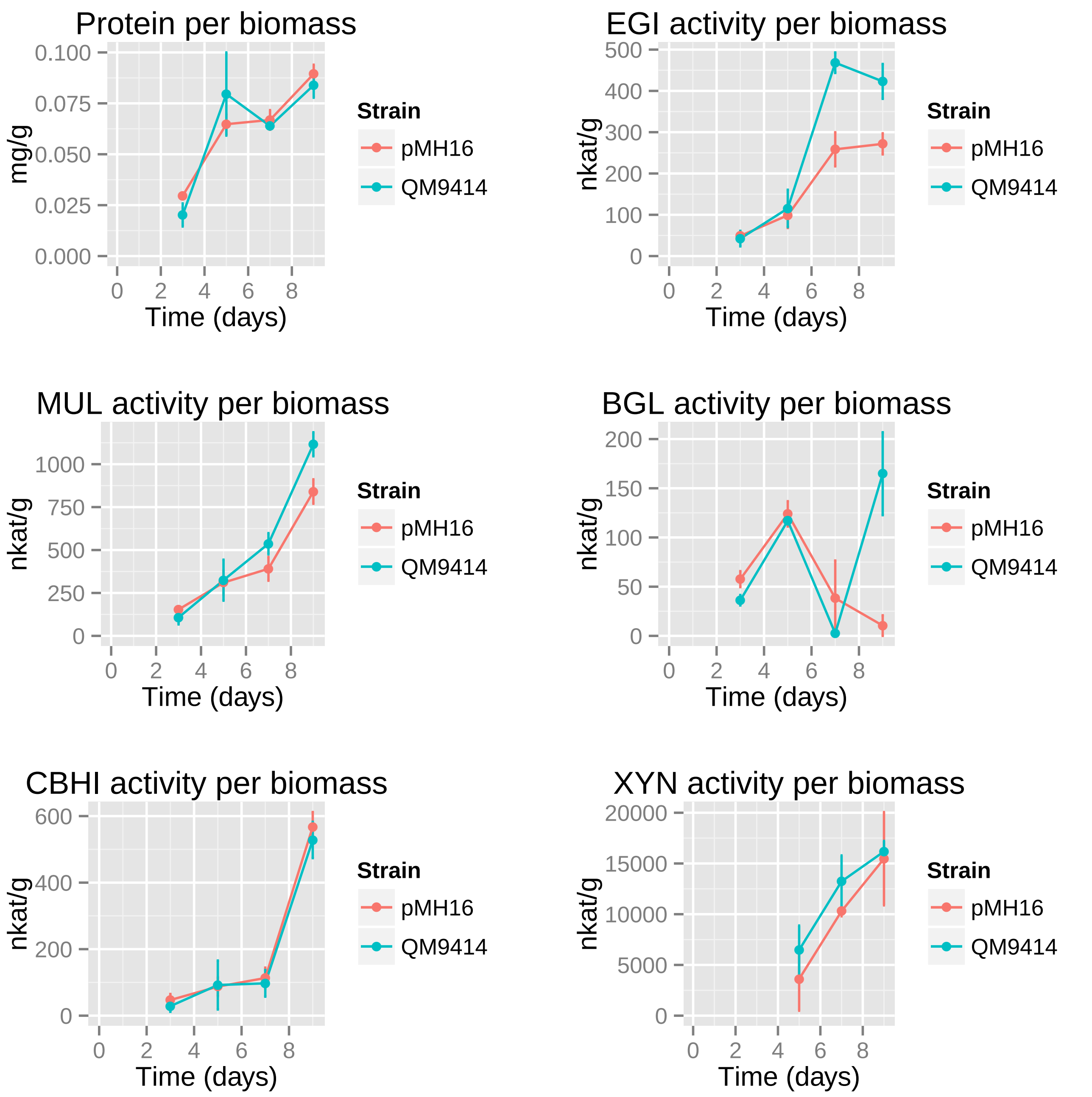

Protein per biomass
mg/g
0.100
0.075
0.050
0.025
0.000
0
2
4
6
8
Time (days)
Strain
pMH16
QM9414
EGI activity per biomass
nkat/g
500
400
300
200
100
0
MUL activity per biomass
1000
750
500
250
BGL activity per biomass
200
150
100
50
CBHI activity per biomass
600
400
200
XYN activity per biomass
20000
15000
10000
5000

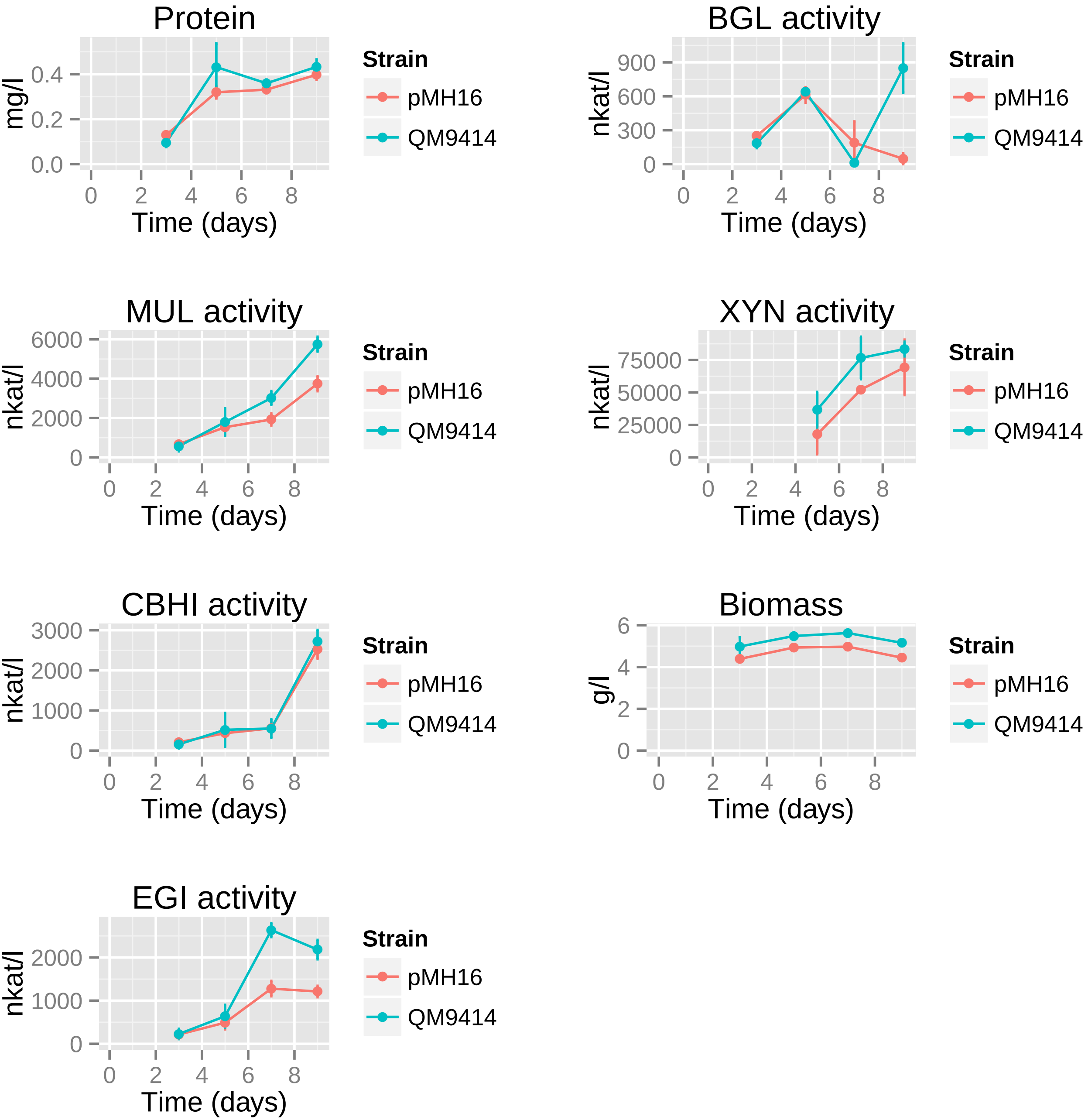
Protein
mg/l
0.0
0.2
0.4
0
2
4
6
8
Time (days)
Strain
pMH16
QM9414
BGL activity
nkat/l
0
300
600
900
Time (days)
Strain
pMH16
QM9414
MUL activity
nkat/l
0
2000
4000
6000
Time (days)
Strain
pMH16
QM9414
XYN activity
nkat/l
0
25000
50000
75000
Time (days)
Strain
pMH16
QM9414
CBHI activity
nkat/l
0
1000
2000
3000
Time (days)
Strain
pMH16
QM9414
Biomass
g/l
0
2
4
6
Time (days)
Strain
pMH16
QM9414
EGI activity
nkat/l
0
1000
2000
Time (days)
Strain
pMH16
QM9414

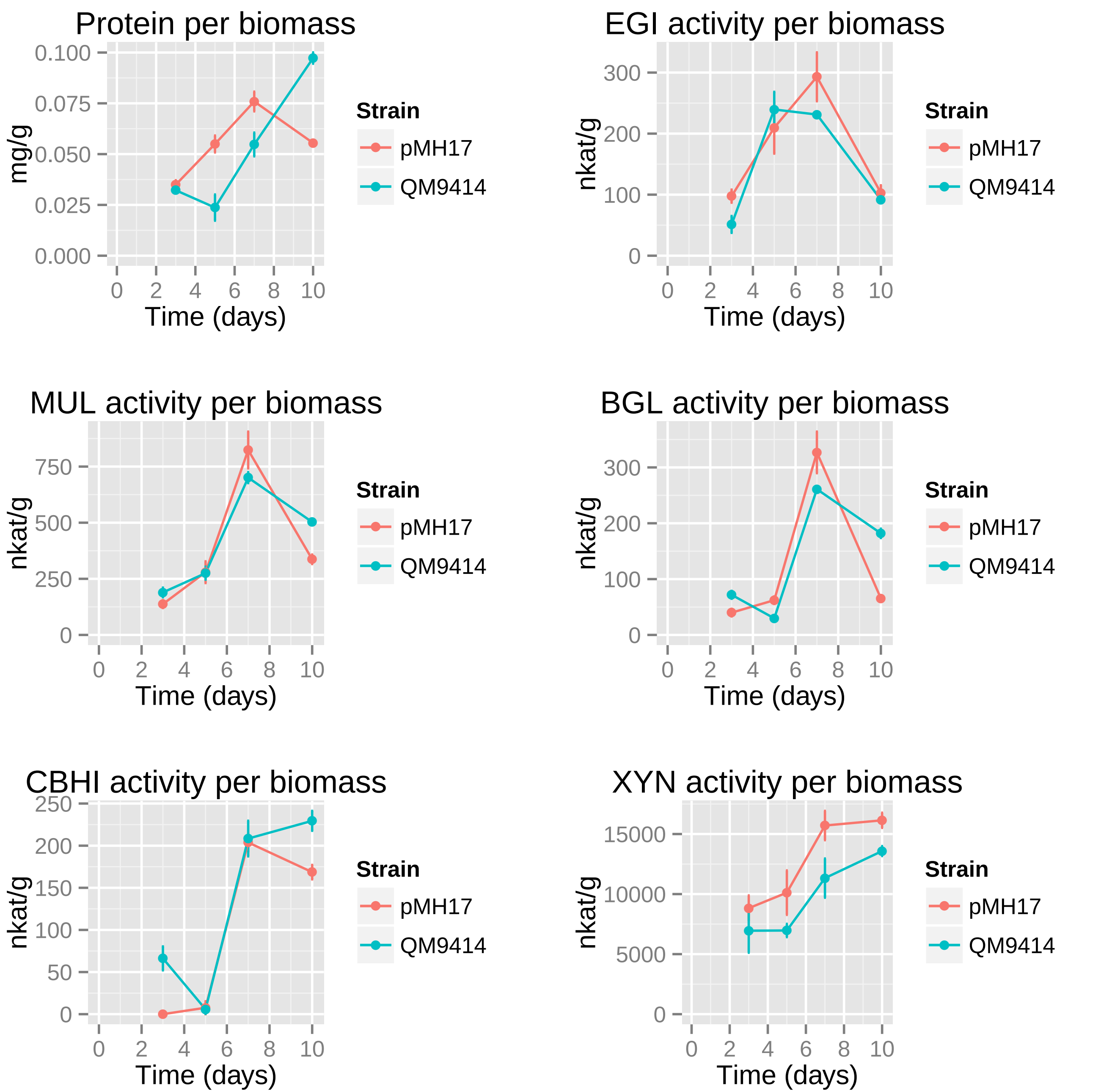
Protein per biomass
mg/g
0.100
0.075
0.050
0.025
0.000
0
2
4
6
8
10
Time (days)
Strain
pMH17
QM9414
EGI activity per biomass
nkat/g
300
200
100
0
Time (days)
MUL activity per biomass
750
500
250
BGL activity per biomass
CBHI activity per biomass
250
200
150
100
50
XYN activity per biomass
15000
10000
5000

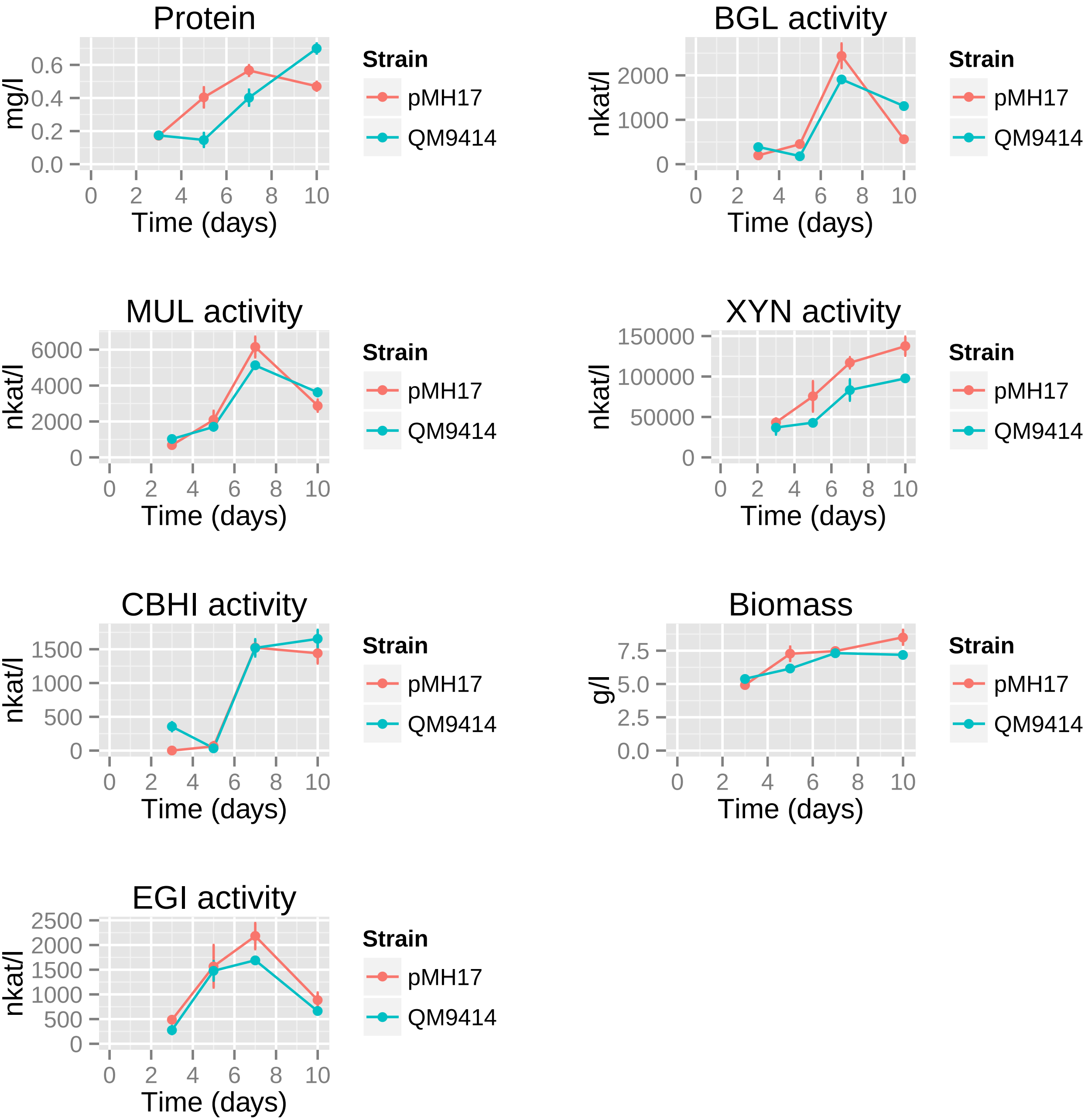
Protein
mg/l
Time (days)
BGL activity
nkat/l
MUL activity
XYN activity
CBHI activity
Biomass
g/l
EGI activity
Strain
pMH17
QM9414

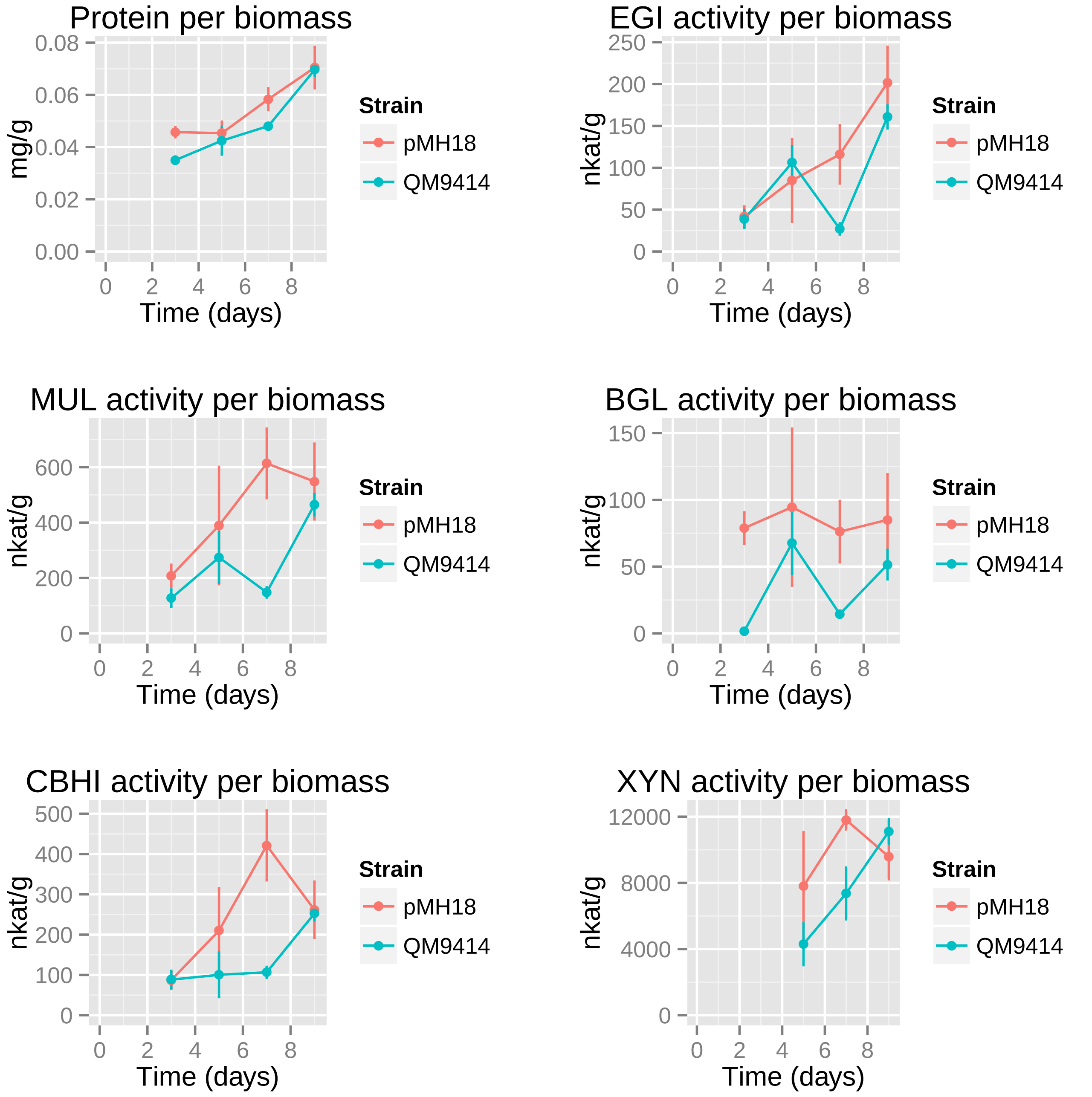
Protein per biomass
mg/g
0.00
0.02
0.04
0.06
0.08
0
2
4
6
8
Time (days)
Strain
pMH18
QM9414
EGI activity per biomass
nkat/g
0
50
100
150
200
250
0
2
4
6
8
Time (days)
Strain
pMH18
QM9414
MUL activity per biomass
nkat/g
0
200
400
600
0
2
4
6
8
Time (days)
Strain
pMH18
QM9414
BGL activity per biomass
nkat/g
0
50
100
150
0
2
4
6
8
Time (days)
Strain
pMH18
QM9414
CBHI activity per biomass
nkat/g
0
100
200
300
400
500
0
2
4
6
8
Time (days)
Strain
pMH18
QM9414
XYN activity per biomass
nkat/g
0
4000
8000
12000
0
2
4
6
8
Time (days)
Strain
pMH18
QM9414

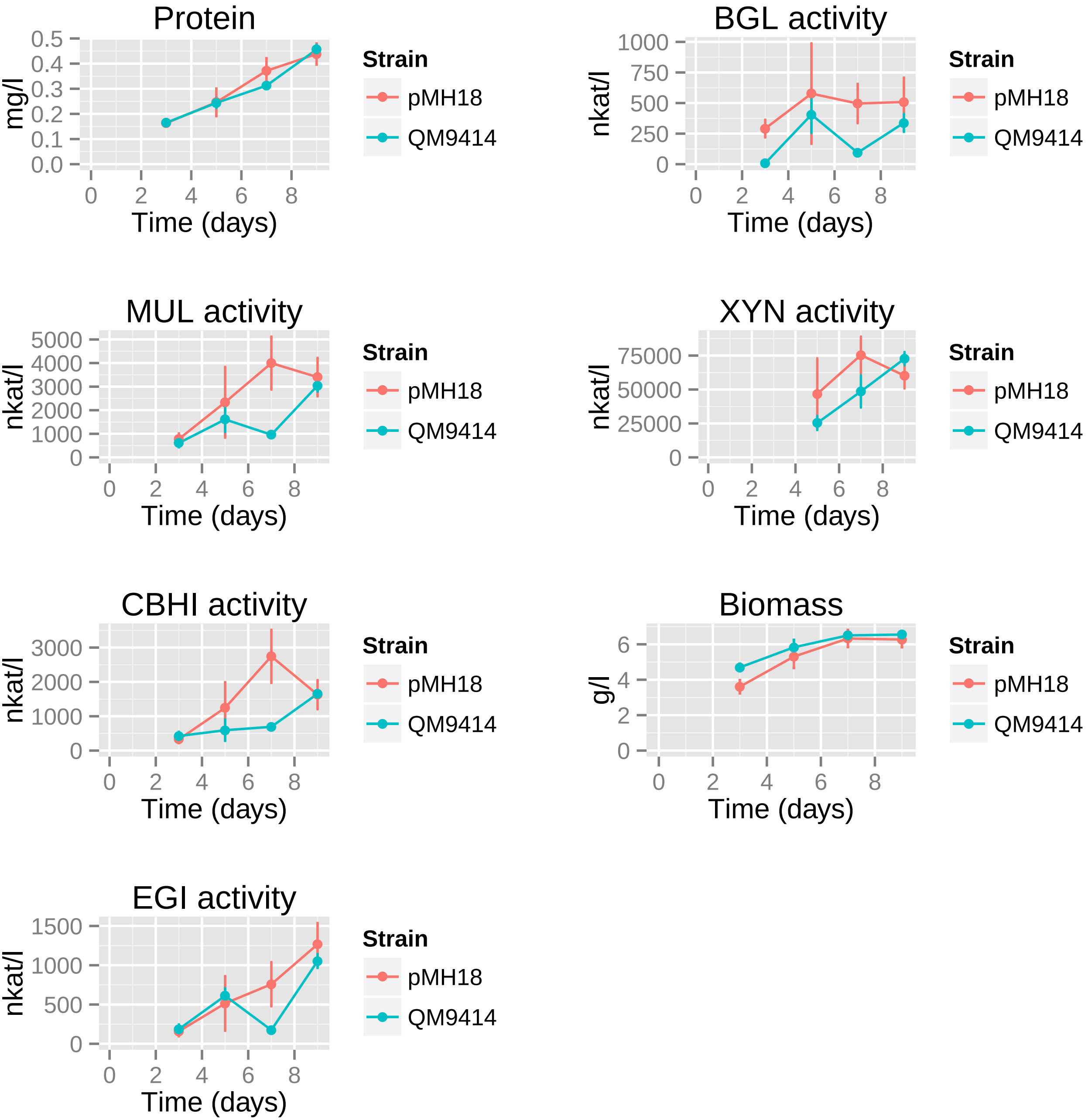
Protein
mg/l
0.0
0.1
0.2
0.3
0.4
0.5
Time (days)
BGL activity
nkat/l
0
250
500
750
1000
MUL activity
0
1000
2000
3000
4000
5000
XYN activity
0
25000
50000
75000
CBHI activity
0
1000
2000
3000
Biomass
g/l
0
2
4
6
EGI activity
0
500
1000
1500
0
2
4
6
8
Strain
pMH18
QM9414

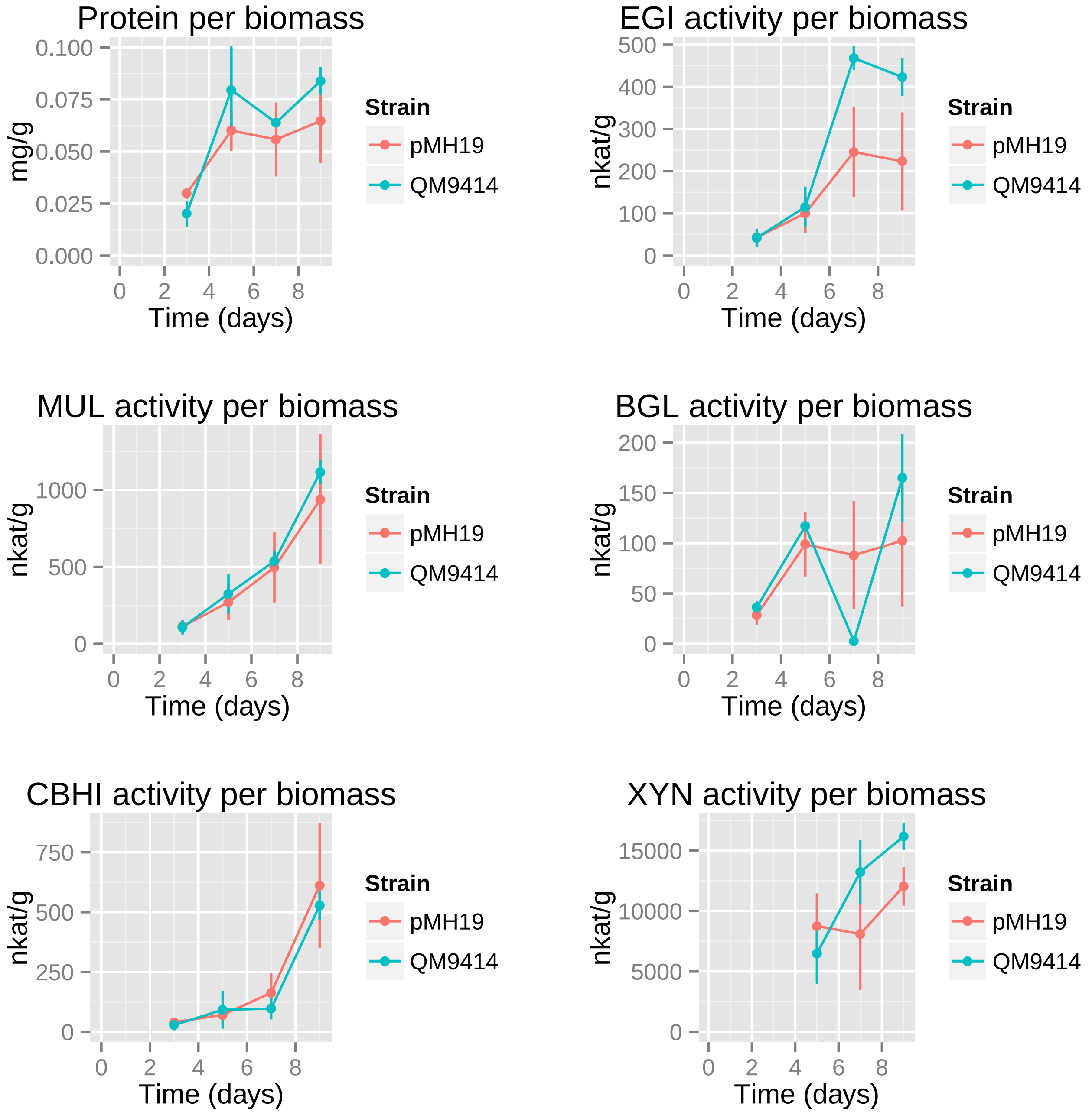
Protein per biomass
mg/g
0.100
0.075
0.050
0.025
0.000
0
2
4
6
8
Time (days)
Strain
pMH19
QM9414
EGI activity per biomass
nkat/g
500
400
300
200
100
0
MUL activity per biomass
1000
500
BGL activity per biomass
200
150
100
50
CBHI activity per biomass
750
250
XYN activity per biomass
15000
10000
5000

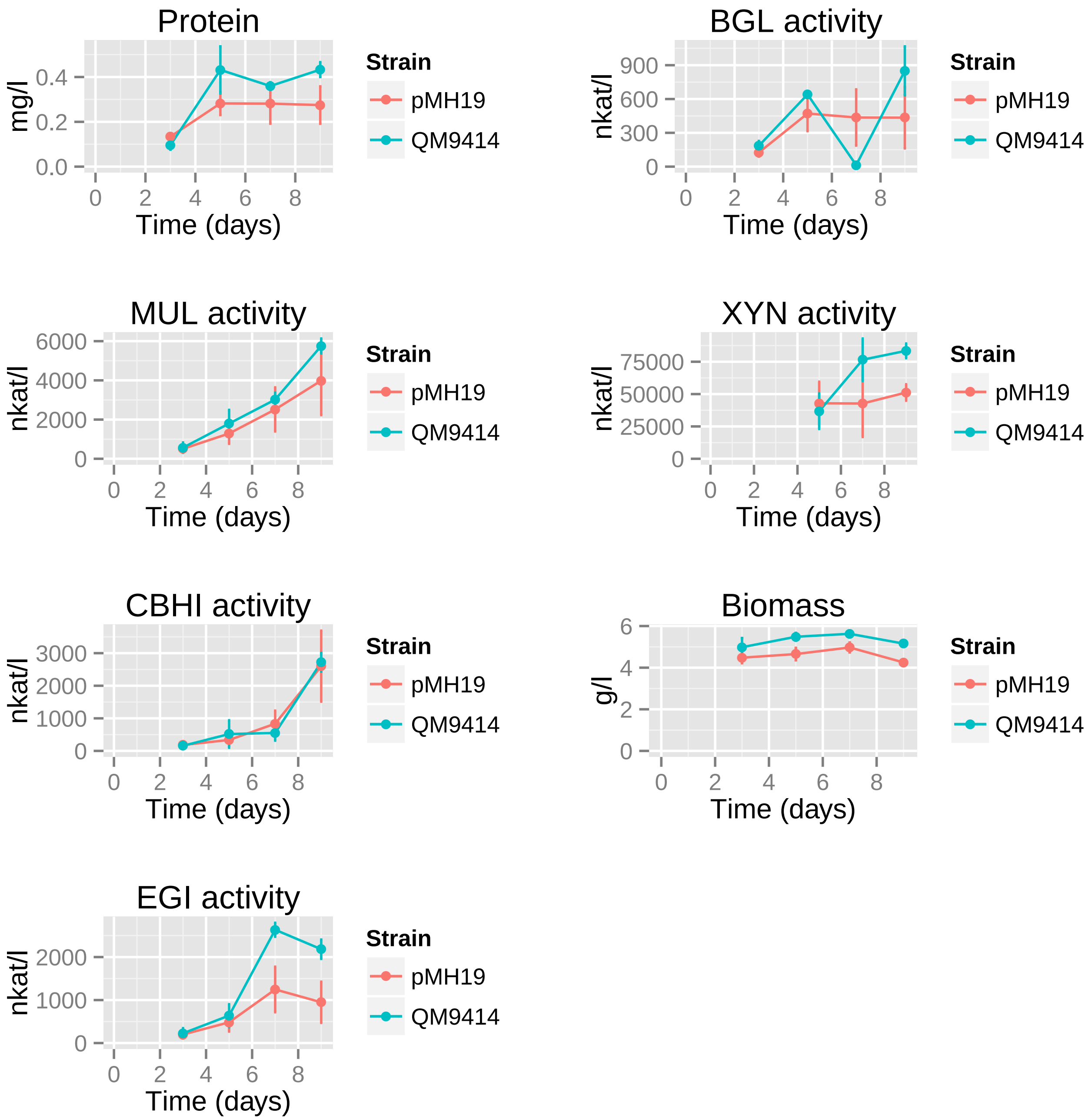
Protein
mg/l
0.0
0.2
0.4
Time (days)
0
2
4
6
8
Strain
pMH19
QM9414
BGL activity
nkat/l
0
300
600
900
Time (days)
Strain
pMH19
QM9414
MUL activity
nkat/l
0
2000
4000
6000
Time (days)
Strain
pMH19
QM9414
XYN activity
nkat/l
0
25000
50000
75000
Time (days)
Strain
pMH19
QM9414
CBHI activity
nkat/l
0
1000
2000
3000
Time (days)
Strain
pMH19
QM9414
Biomass
g/l
0
2
4
6
Time (days)
Strain
pMH19
QM9414
EGI activity
nkat/l
0
1000
2000
Time (days)
Strain
pMH19
QM9414

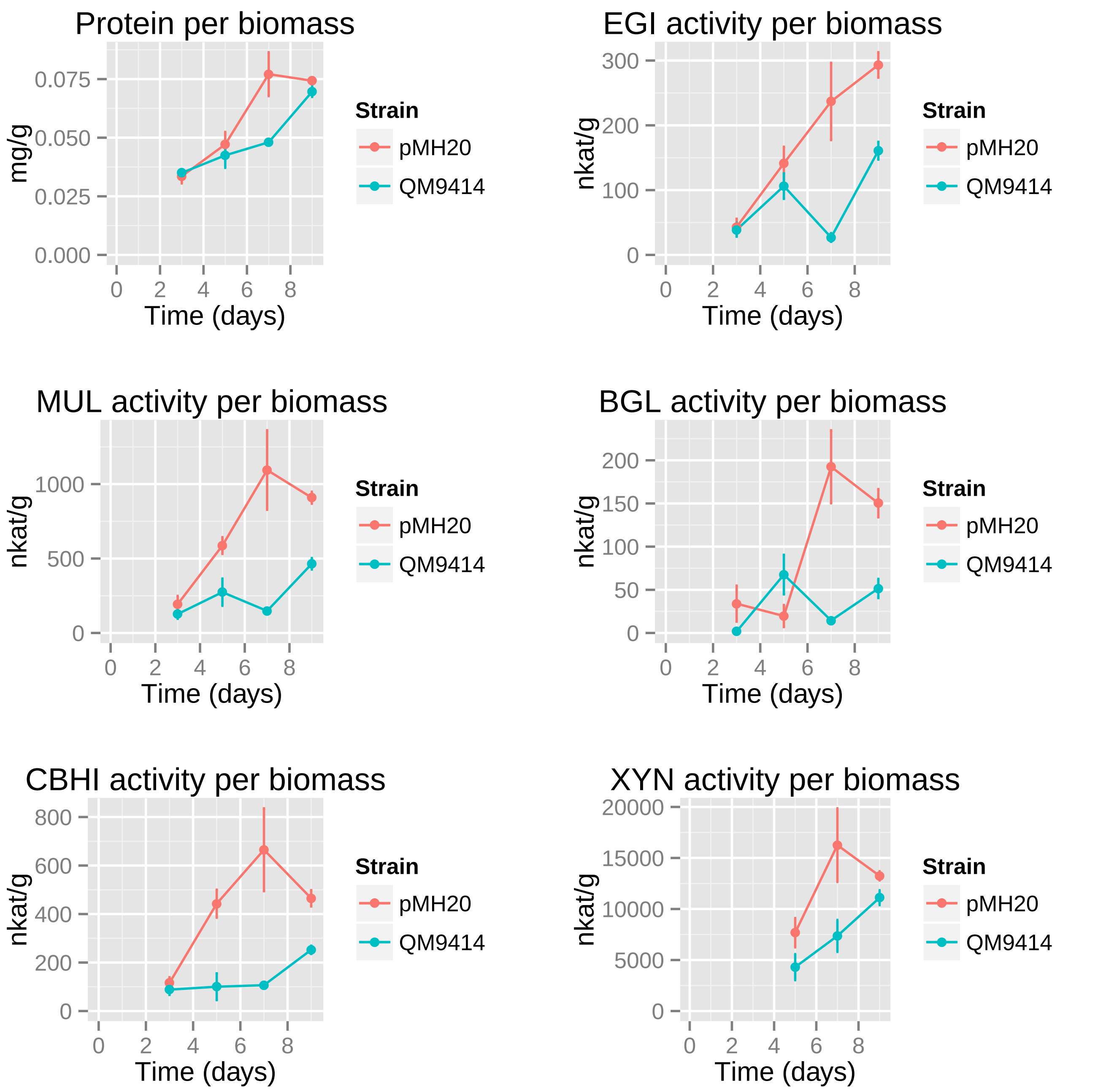

Protein per biomass
mg/g
0.000
0.025
0.050
0.075
Time (days)
EGI activity per biomass
MUL activity per biomass
BGL activity per biomass
CBHI activity per biomass
XYN activity per biomass
nkat/g
Strain
pMH20
QM9414

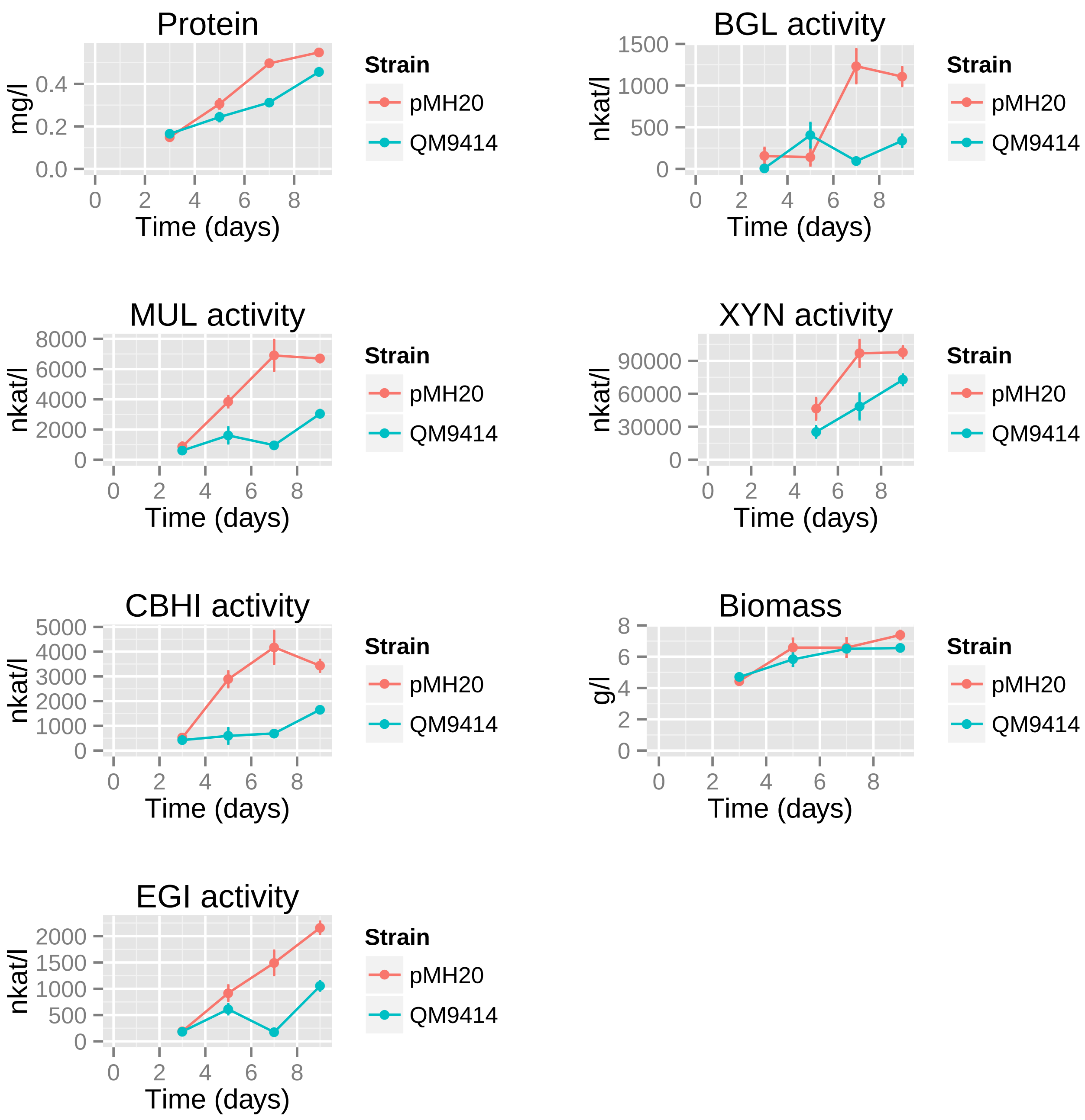
Protein
mg/l
Time (days)
BGL activity
nkat/l
MUL activity
XYN activity
CBHI activity
Biomass
g/l
EGI activity
Strain
pMH20
QM9414

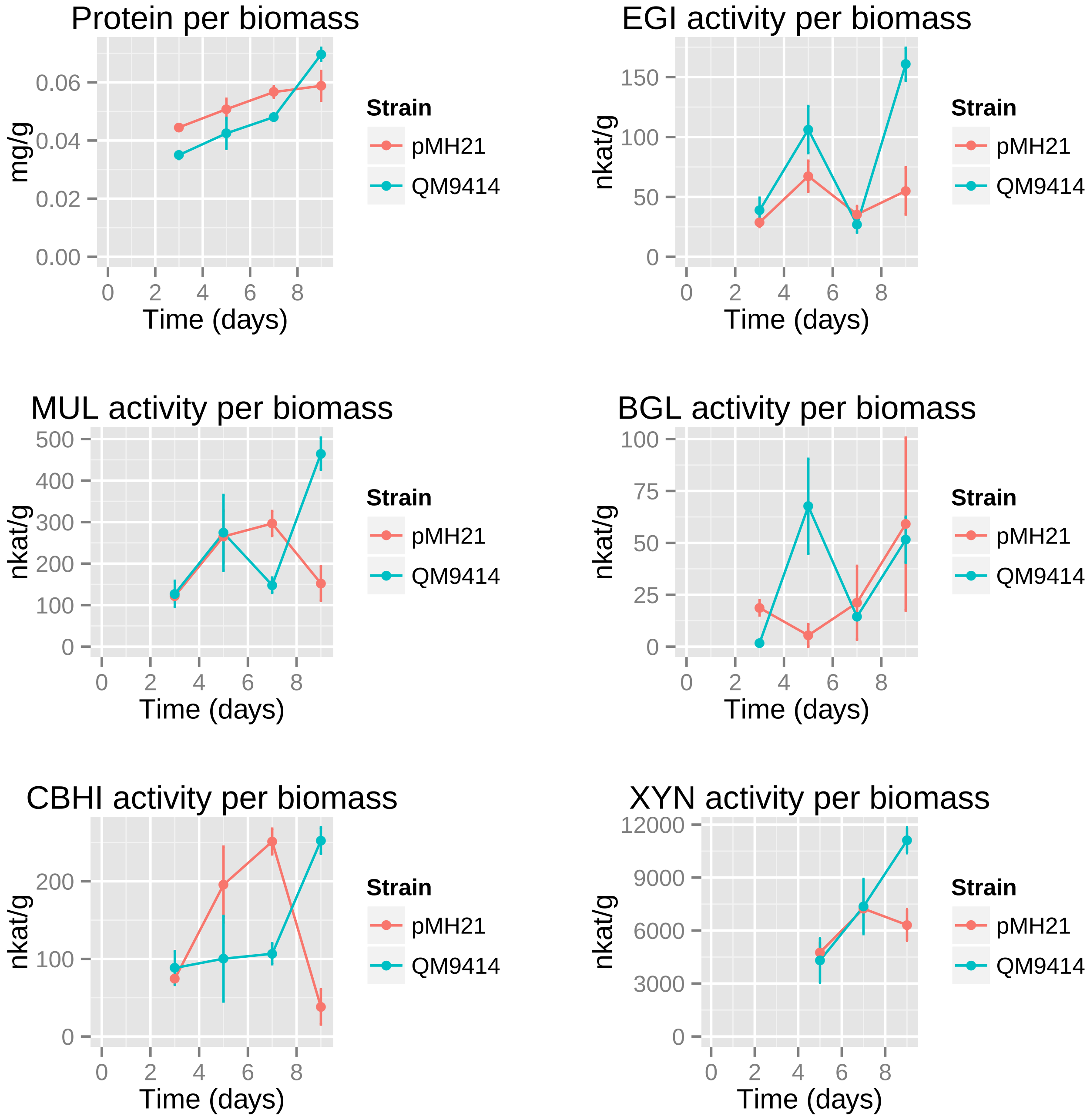

Protein per biomass
mg/g
0.00
0.02
0.04
0.06
Time (days)
0
2
4
6
8
Strain
pMH21
QM9414
EGI activity per biomass
nkat/g
0
50
100
150
MUL activity per biomass
0
100
200
300
400
500
BGL activity per biomass
0
25
50
75
100
CBHI activity per biomass
0
100
200
XYN activity per biomass
0
3000
6000
9000
12000

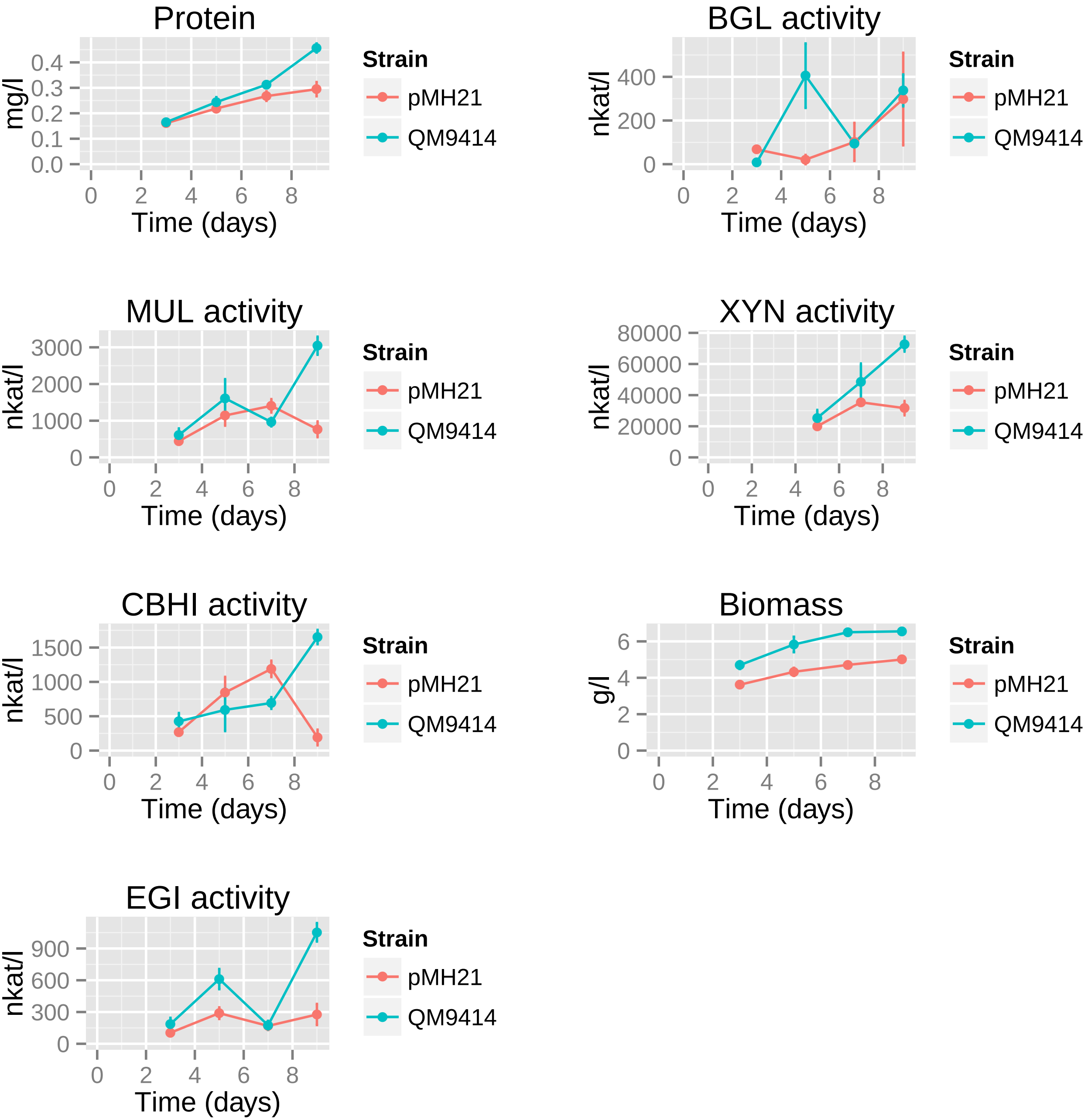
Protein
mg/l
0.0
0.1
0.2
0.3
0.4
0
2
4
6
8
Time (days)
Strain
pMH21
QM9414
BGL activity
nkat/l
0
200
400
0
2
4
6
8
Time (days)
Strain
pMH21
QM9414
MUL activity
nkat/l
0
1000
2000
3000
0
2
4
6
8
Time (days)
Strain
pMH21
QM9414
XYN activity
nkat/l
0
20000
40000
60000
80000
0
2
4
6
8
Time (days)
Strain
pMH21
QM9414
CBHI activity
nkat/l
0
500
1000
1500
0
2
4
6
8
Time (days)
Strain
pMH21
QM9414
Biomass
g/l
0
2
4
6
0
2
4
6
8
Time (days)
Strain
pMH21
QM9414
EGI activity
nkat/l
0
300
600
900
0
2
4
6
8
Time (days)
Strain
pMH21
QM9414

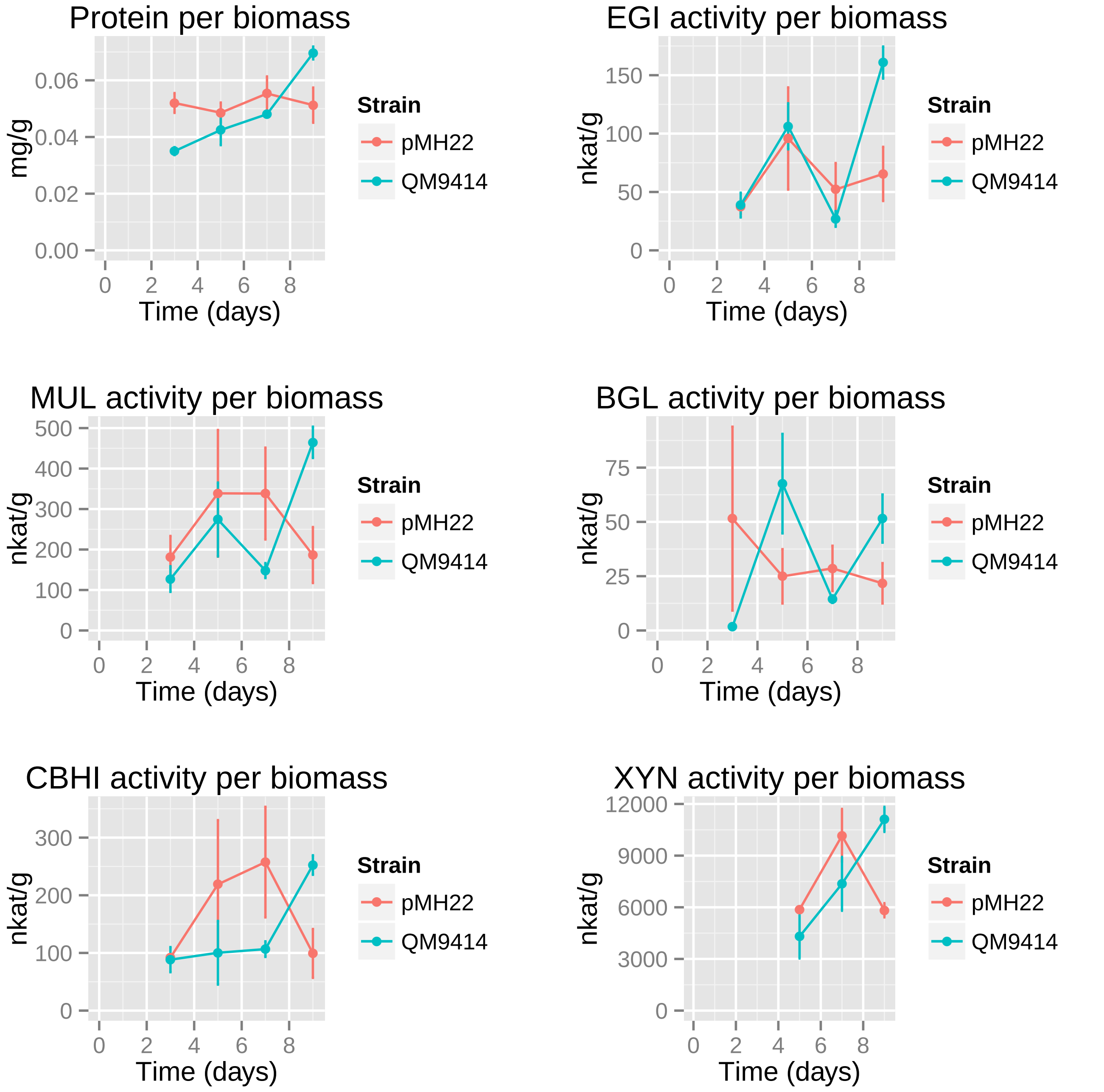

Protein per biomass
mg/g
0.00
0.02
0.04
0.06
Time (days)
EGI activity per biomass
nkat/g
0
50
100
150
MUL activity per biomass
0
100
200
300
400
500
BGL activity per biomass
0
25
50
75
CBHI activity per biomass
0
100
200
300
XYN activity per biomass
0
3000
6000
9000
12000
0
2
4
6
8
Strain
pMH22
QM9414

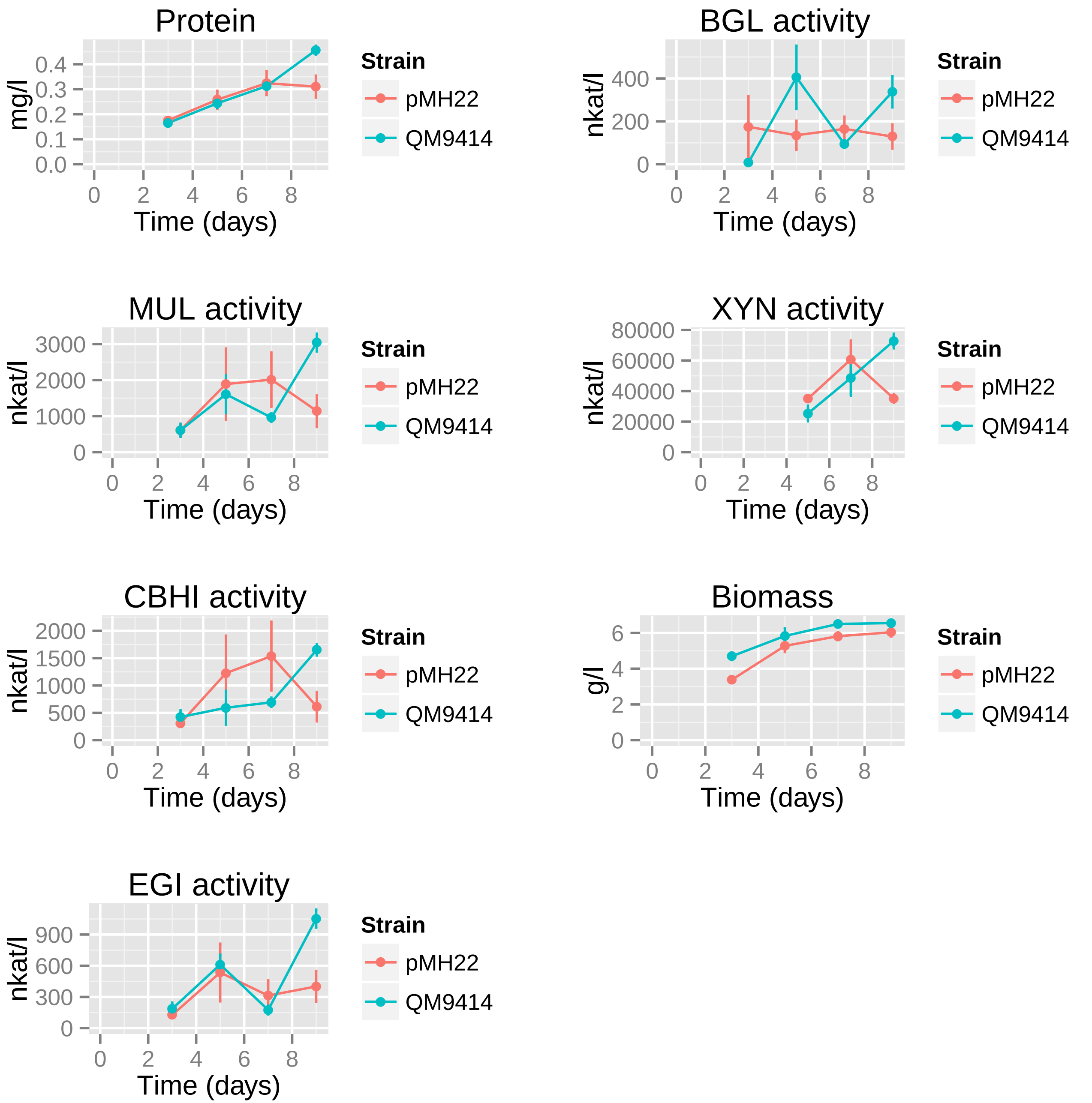
Protein
mg/l
0.0
0.1
0.2
0.3
0.4
0
2
4
6
8
Time (days)
Strain
pMH22
QM9414
BGL activity
nkat/l
0
200
400
0
2
4
6
8
Time (days)
Strain
pMH22
QM9414
MUL activity
nkat/l
0
1000
2000
3000
0
2
4
6
8
Time (days)
Strain
pMH22
QM9414
XYN activity
nkat/l
0
20000
40000
60000
80000
0
2
4
6
8
Time (days)
Strain
pMH22
QM9414
CBHI activity
nkat/l
0
500
1000
1500
2000
0
2
4
6
8
Time (days)
Strain
pMH22
QM9414
Biomass
g/l
0
2
4
6
0
2
4
6
8
Time (days)
Strain
pMH22
QM9414
EGI activity
nkat/l
0
300
600
900
0
2
4
6
8
Time (days)
Strain
pMH22
QM9414

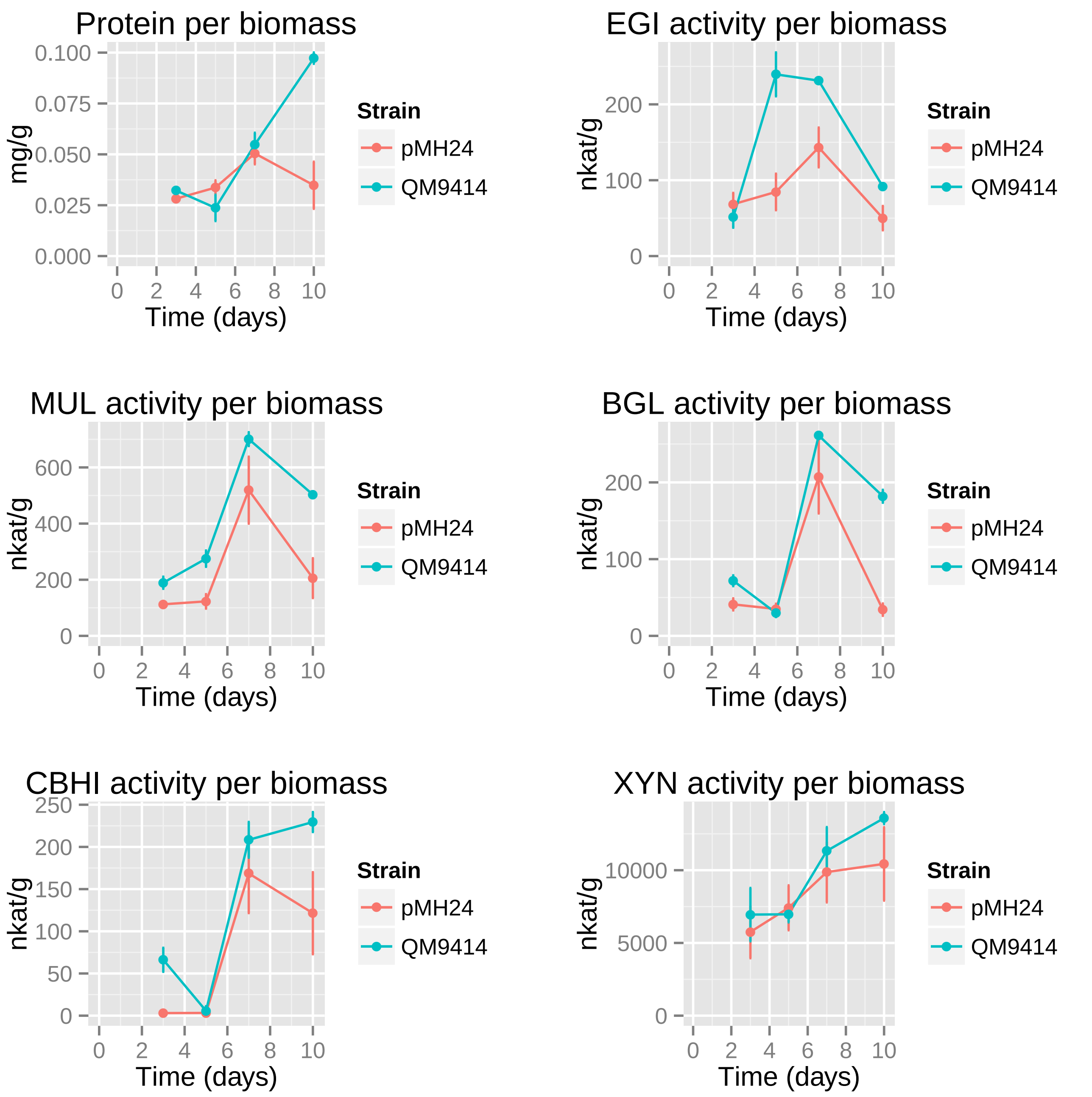

Protein per biomass
mg/g
0.100
0.075
0.050
0.025
0.000
0
2
4
6
8
10
Time (days)
Strain
pMH24
QM9414
EGI activity per biomass
nkat/g
200
100
0
MUL activity per biomass
600
400
200
BGL activity per biomass
CBHI activity per biomass
250
150
50
XYN activity per biomass
10000
5000

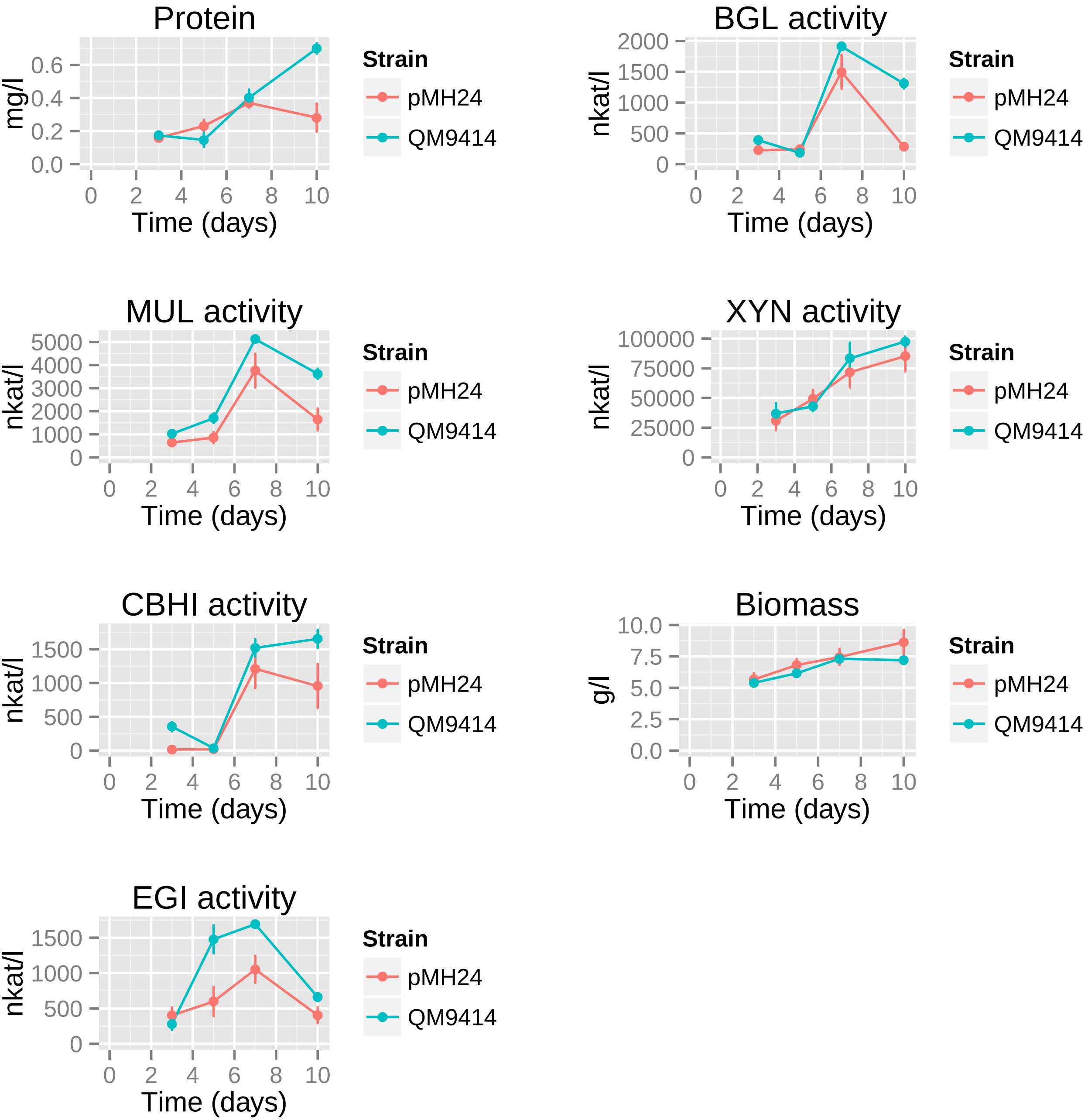
Protein
mg/l
0.0
0.2
0.4
0.6
Time (days)
0
2
4
6
8
10
BGL activity
nkat/l
0
500
1000
1500
2000
MUL activity
5000
4000
3000
2000
1000
XYN activity
100000
75000
50000
25000
CBHI activity
Biomass
g/l
10.0
7.5
5.0
2.5
EGI activity
Strain
pMH24
QM9414

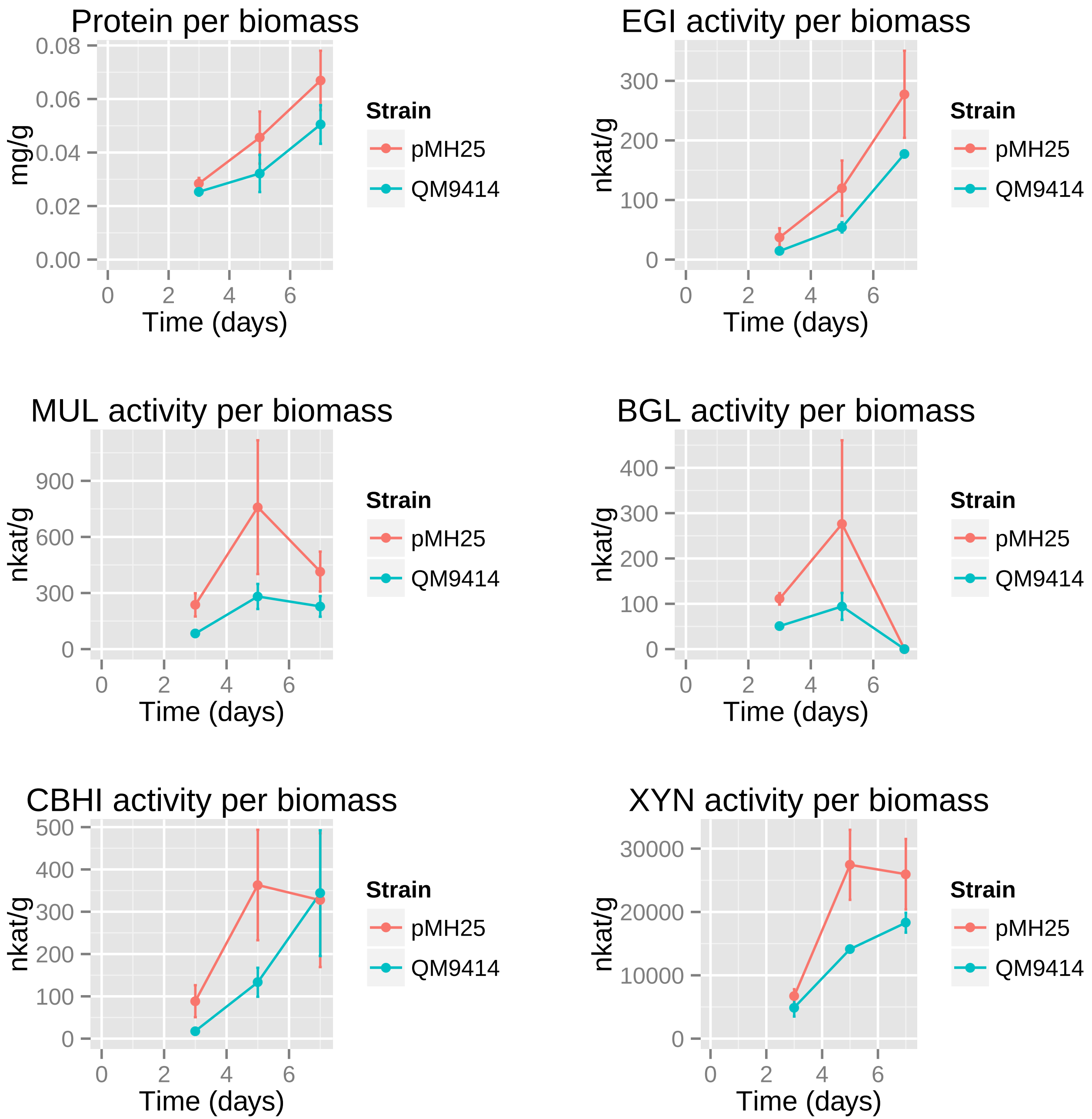
Protein per biomass
mg/g
0.00
0.02
0.04
0.06
0.08
0
2
4
6
Time (days)
Strain
pMH25
QM9414
EGI activity per biomass
nkat/g
0
100
200
300
Time (days)
Strain
pMH25
QM9414
MUL activity per biomass
nkat/g
0
300
600
900
Time (days)
Strain
pMH25
QM9414
BGL activity per biomass
nkat/g
0
100
200
300
400
Time (days)
Strain
pMH25
QM9414
CBHI activity per biomass
nkat/g
0
100
200
300
400
500
Time (days)
Strain
pMH25
QM9414
XYN activity per biomass
nkat/g
0
10000
20000
30000
Time (days)
Strain
pMH25
QM9414

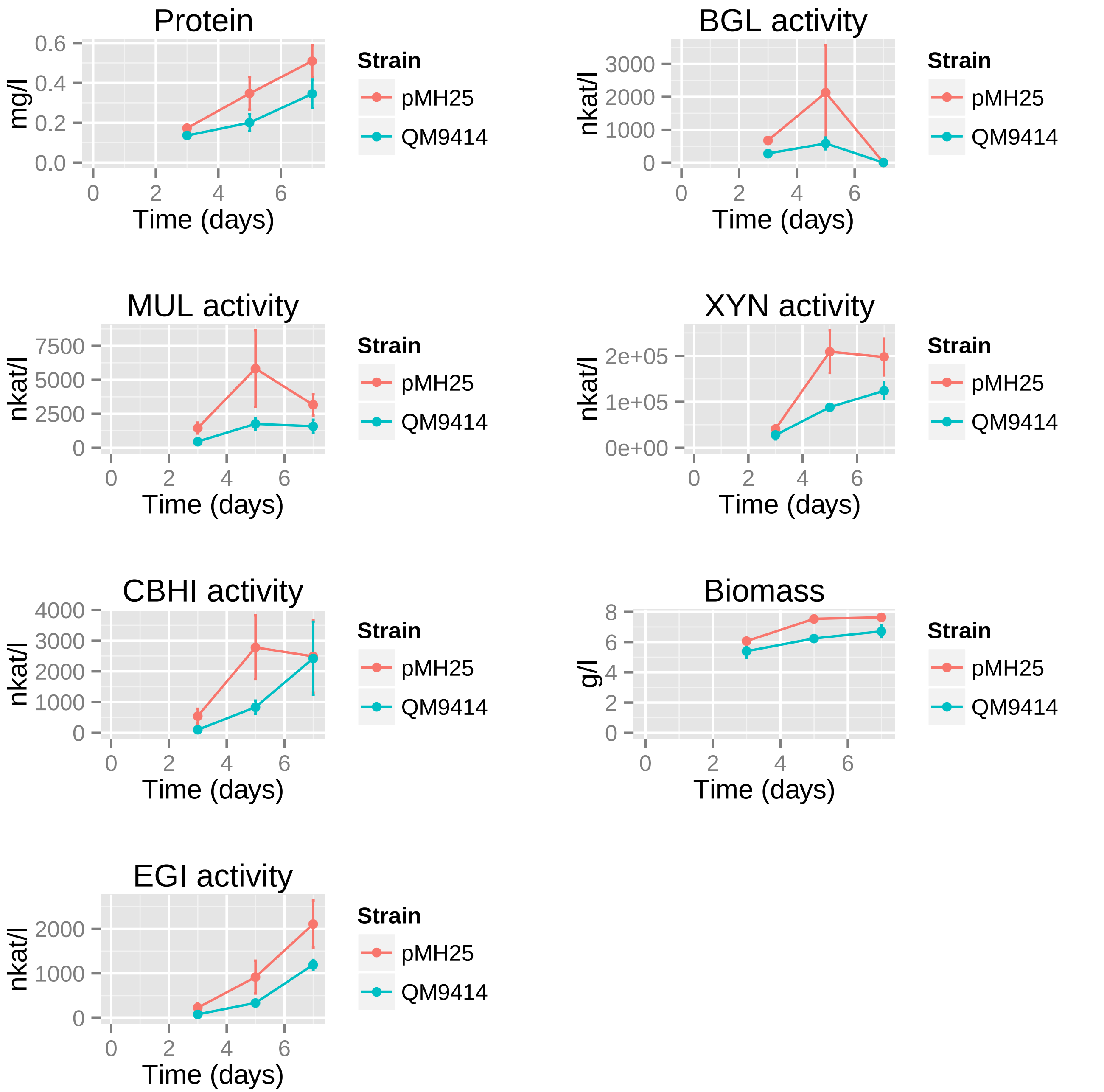

Protein
mg/l
0.0
0.2
0.4
0.6
Time (days)
0
2
4
6
Strain
pMH25
QM9414
BGL activity
nkat/l
0
1000
2000
3000
Time (days)
MUL activity
nkat/l
0
2500
5000
7500
Time (days)
XYN activity
nkat/l
0e+00
1e+05
2e+05
Time (days)
CBHI activity
nkat/l
0
1000
2000
3000
4000
Time (days)
Biomass
g/l
0
2
4
6
8
Time (days)
EGI activity
nkat/l
0
1000
2000
Time (days)

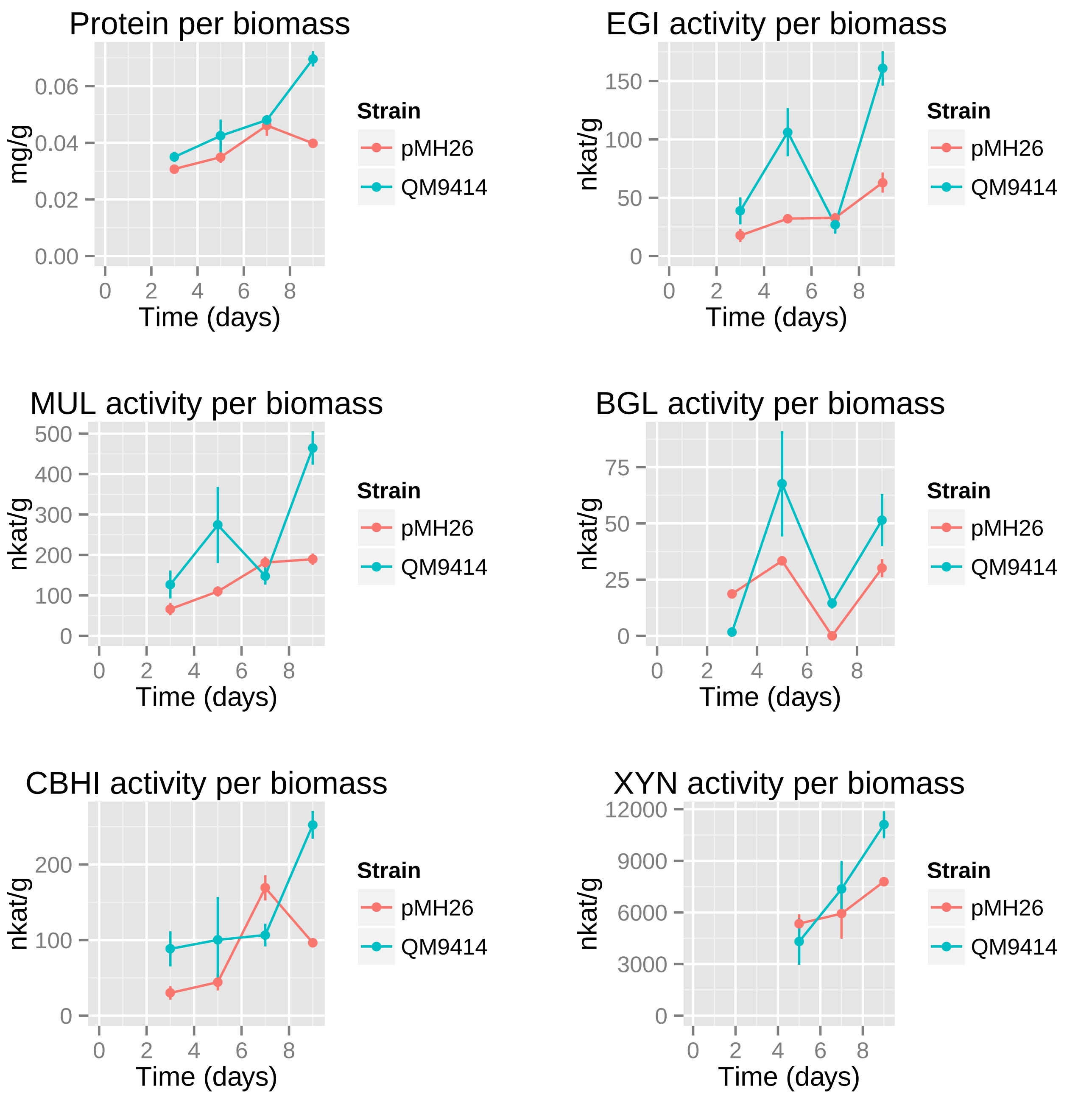
Protein per biomass
mg/g
0.00
0.02
0.04
0.06
Time (days)
0
2
4
6
8
Strain
pMH26
QM9414
EGI activity per biomass
nkat/g
0
50
100
150
MUL activity per biomass
0
100
200
300
400
500
BGL activity per biomass
0
25
50
75
CBHI activity per biomass
0
100
200
XYN activity per biomass
0
3000
6000
9000
12000

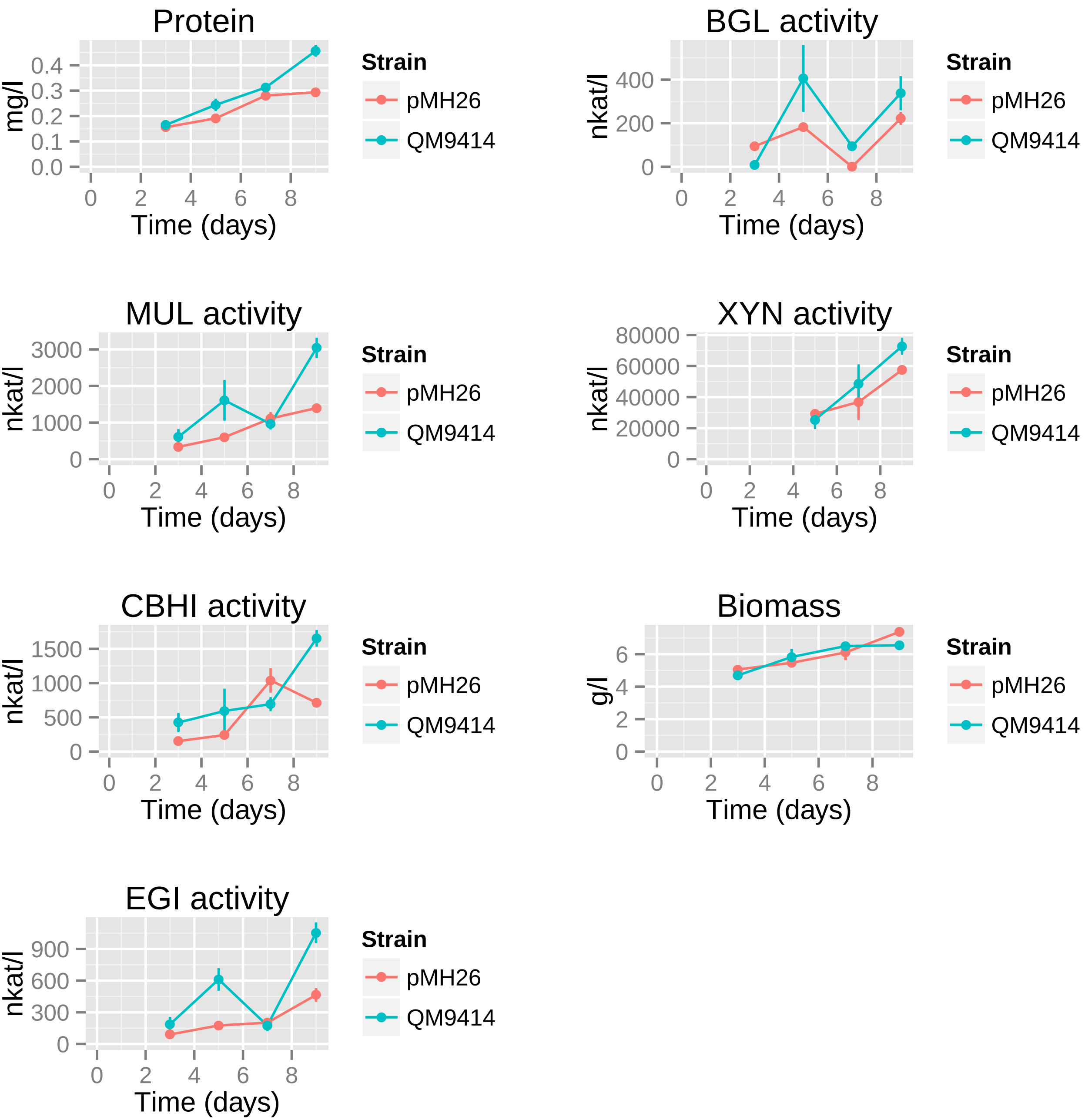
Protein
mg/l
0.0
0.1
0.2
0.3
0.4
Time (days)
0
2
4
6
8
Strain
pMH26
QM9414
BGL activity
nkat/l
0
200
400
Time (days)
Strain
pMH26
QM9414
MUL activity
nkat/l
0
1000
2000
3000
Time (days)
Strain
pMH26
QM9414
XYN activity
nkat/l
0
20000
40000
60000
80000
Time (days)
Strain
pMH26
QM9414
CBHI activity
nkat/l
0
500
1000
1500
Time (days)
Strain
pMH26
QM9414
Biomass
g/l
0
2
4
6
Time (days)
Strain
pMH26
QM9414
EGI activity
nkat/l
0
300
600
900
Time (days)
Strain
pMH26
QM9414

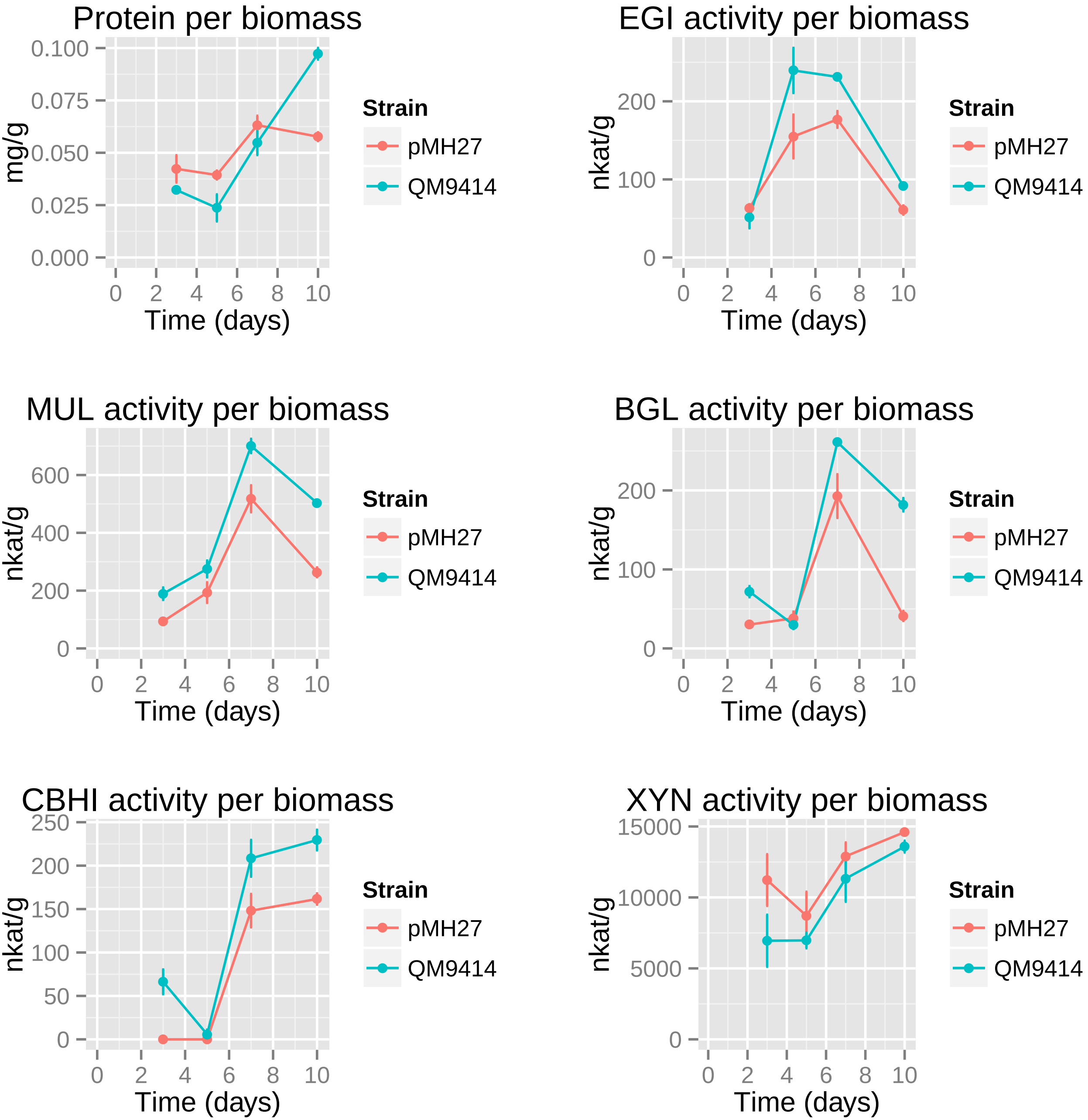
Protein per biomass
mg/g
0.000
0.025
0.050
0.075
0.100
EGI activity per biomass
MUL activity per biomass
BGL activity per biomass
CBHI activity per biomass
XYN activity per biomass
nkat/g
0
50
100
150
200
250
400
600
5000
10000
15000
0 2 4 6 8 10
Time (days)
Strain
pMH27
QM9414

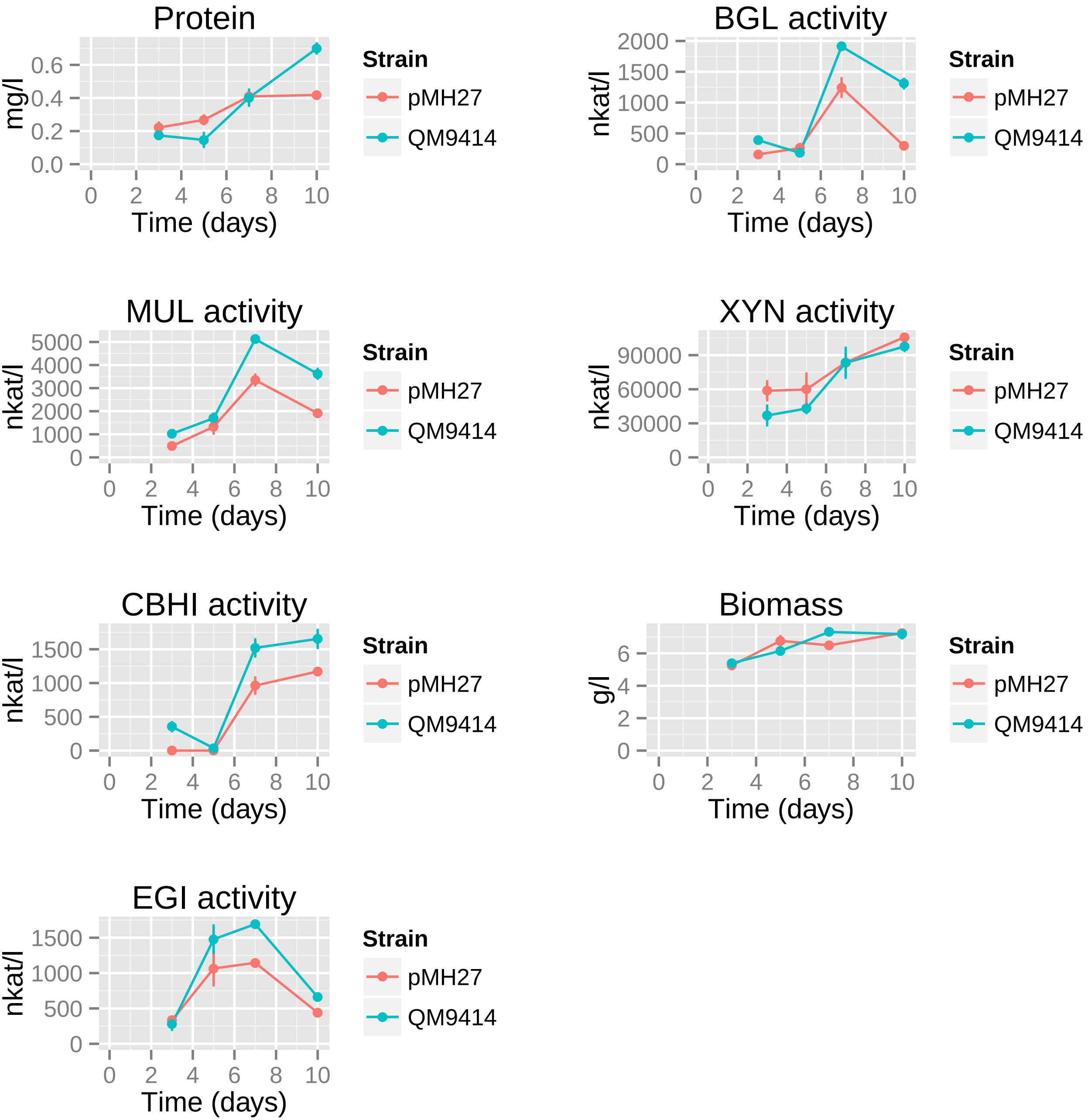
Protein
mg/l
0.0
0.2
0.4
0.6
Time (days)
0
2
4
6
8
10
Strain
pMH27
QM9414
BGL activity
nkat/l
0
500
1000
1500
2000
MUL activity
0
1000
2000
3000
4000
5000
XYN activity
30000
60000
90000
CBHI activity
Biomass
g/l
EGI activity

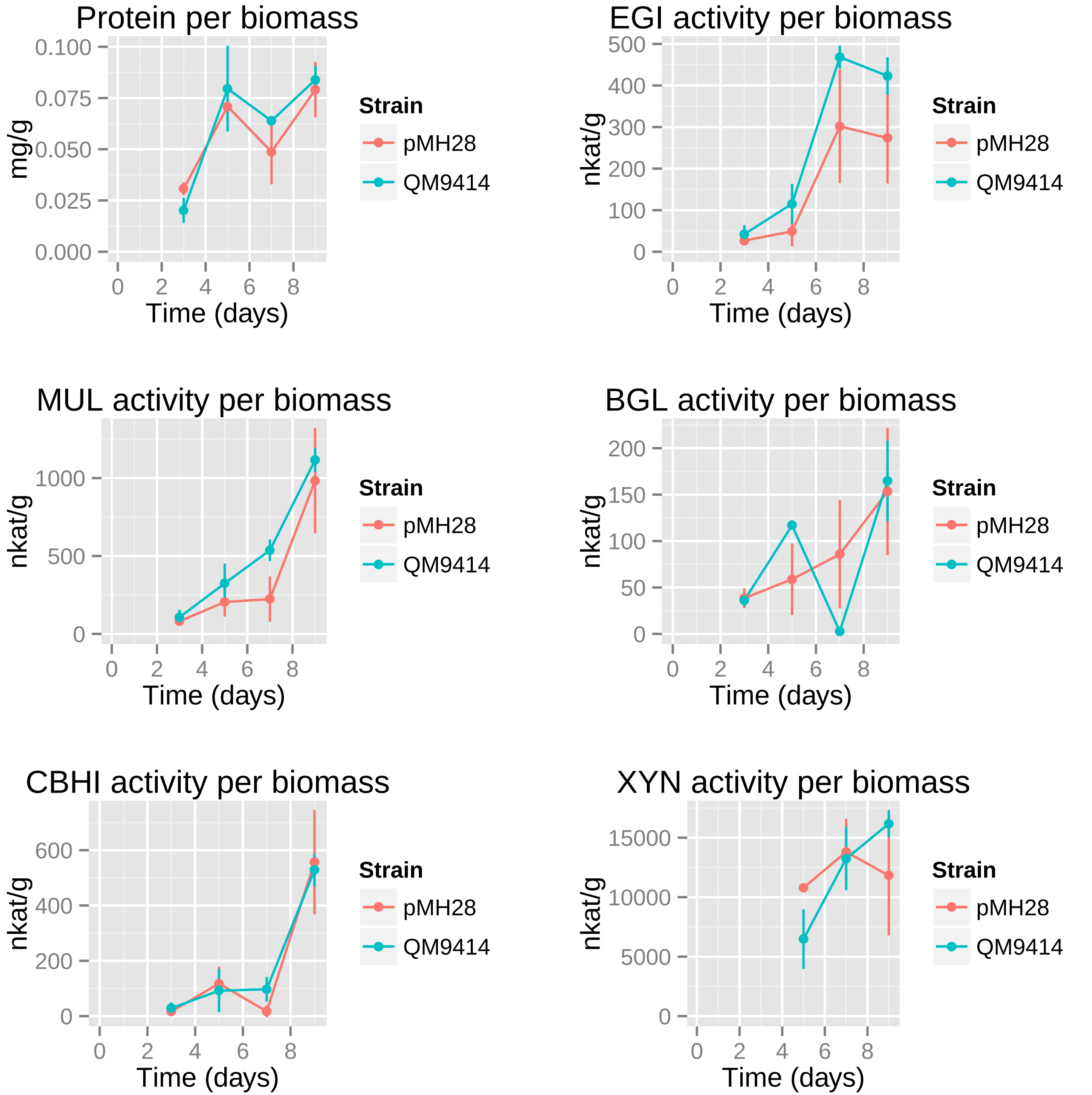

Protein per biomass
mg/g
0.100
0.075
0.050
0.025
0.000
0
2
4
6
8
Time (days)
Strain
pMH28
QM9414
EGI activity per biomass
nkat/g
500
400
300
200
100
0
Time (days)
MUL activity per biomass
1000
500
BGL activity per biomass
200
150
100
50
CBHI activity per biomass
600
400
200
XYN activity per biomass
15000
10000
5000

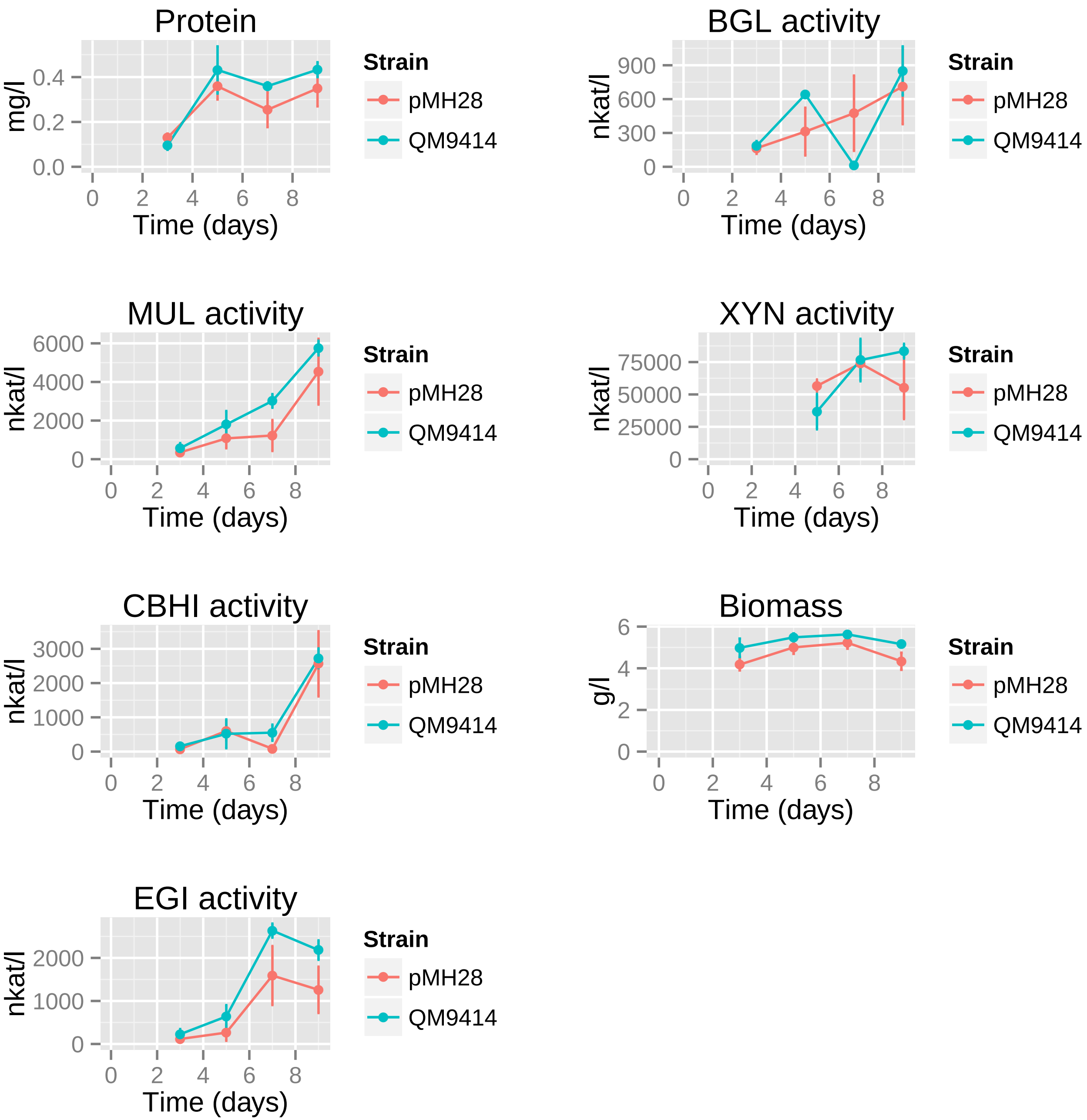
Protein
mg/l
0.0
0.2
0.4
0 2 4 6 8
Time (days)
Strain
pMH28
QM9414
BGL activity
nkat/l
0
300
600
900
0 2 4 6 8
Time (days)
Strain
pMH28
QM9414
MUL activity
nkat/l
0
2000
4000
6000
0 2 4 6 8
Time (days)
Strain
pMH28
QM9414
XYN activity
nkat/l
0
25000
50000
75000
0 2 4 6 8
Time (days)
Strain
pMH28
QM9414
CBHI activity
nkat/l
0
1000
2000
3000
0 2 4 6 8
Time (days)
Strain
pMH28
QM9414
Biomass
g/l
0
2
4
6
0 2 4 6 8
Time (days)
Strain
pMH28
QM9414
EGI activity
nkat/l
0
1000
2000
0 2 4 6 8
Time (days)
Strain
pMH28
QM9414

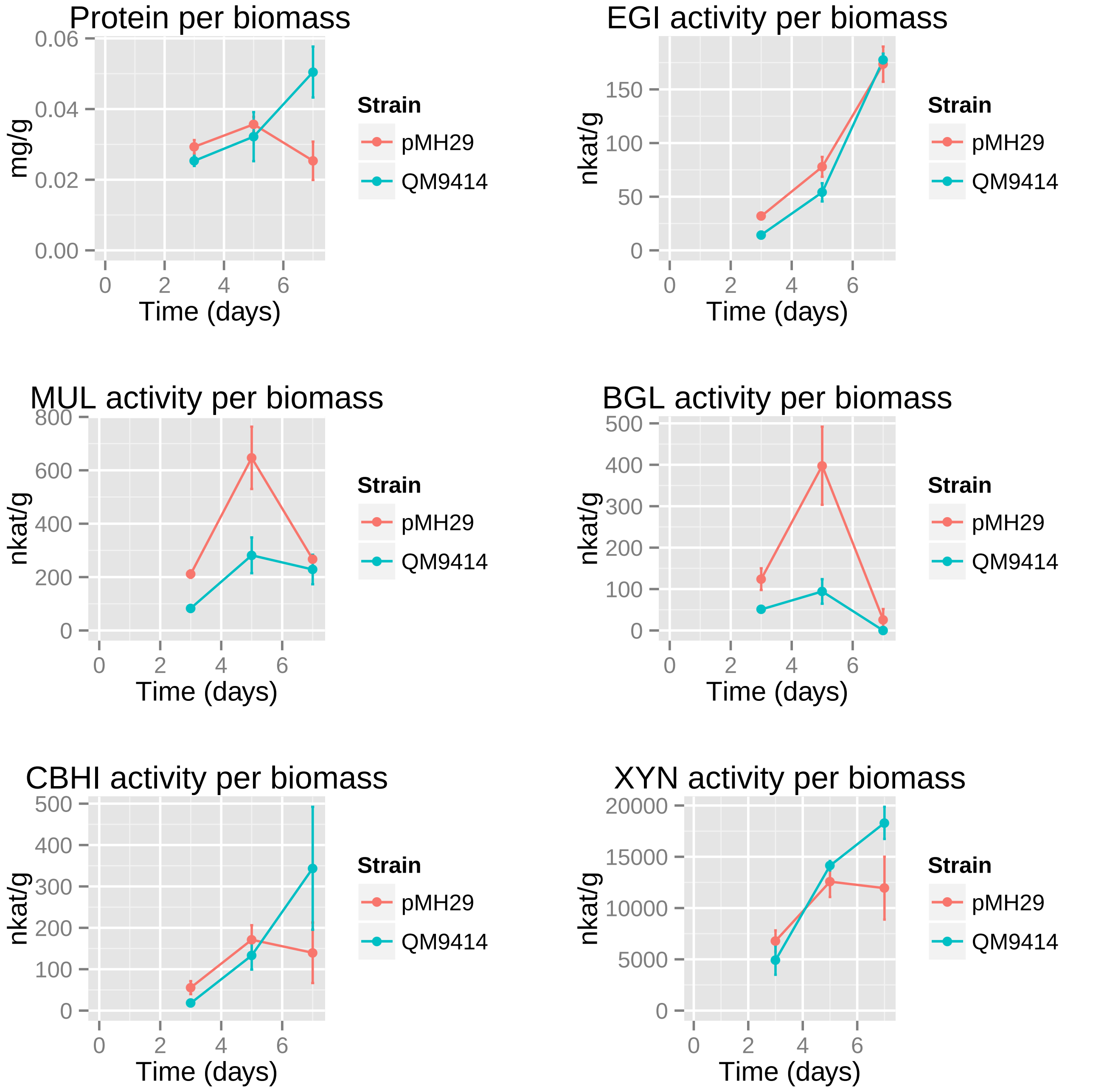

Protein per biomass
mg/g
0.00
0.02
0.04
0.06
Time (days)
0
2
4
6
Strain
pMH29
QM9414
EGI activity per biomass
nkat/g
0
50
100
150
MUL activity per biomass
nkat/g
0
200
400
600
800
BGL activity per biomass
nkat/g
0
100
200
300
400
500
CBHI activity per biomass
nkat/g
XYN activity per biomass
nkat/g
0
5000
10000
15000
20000

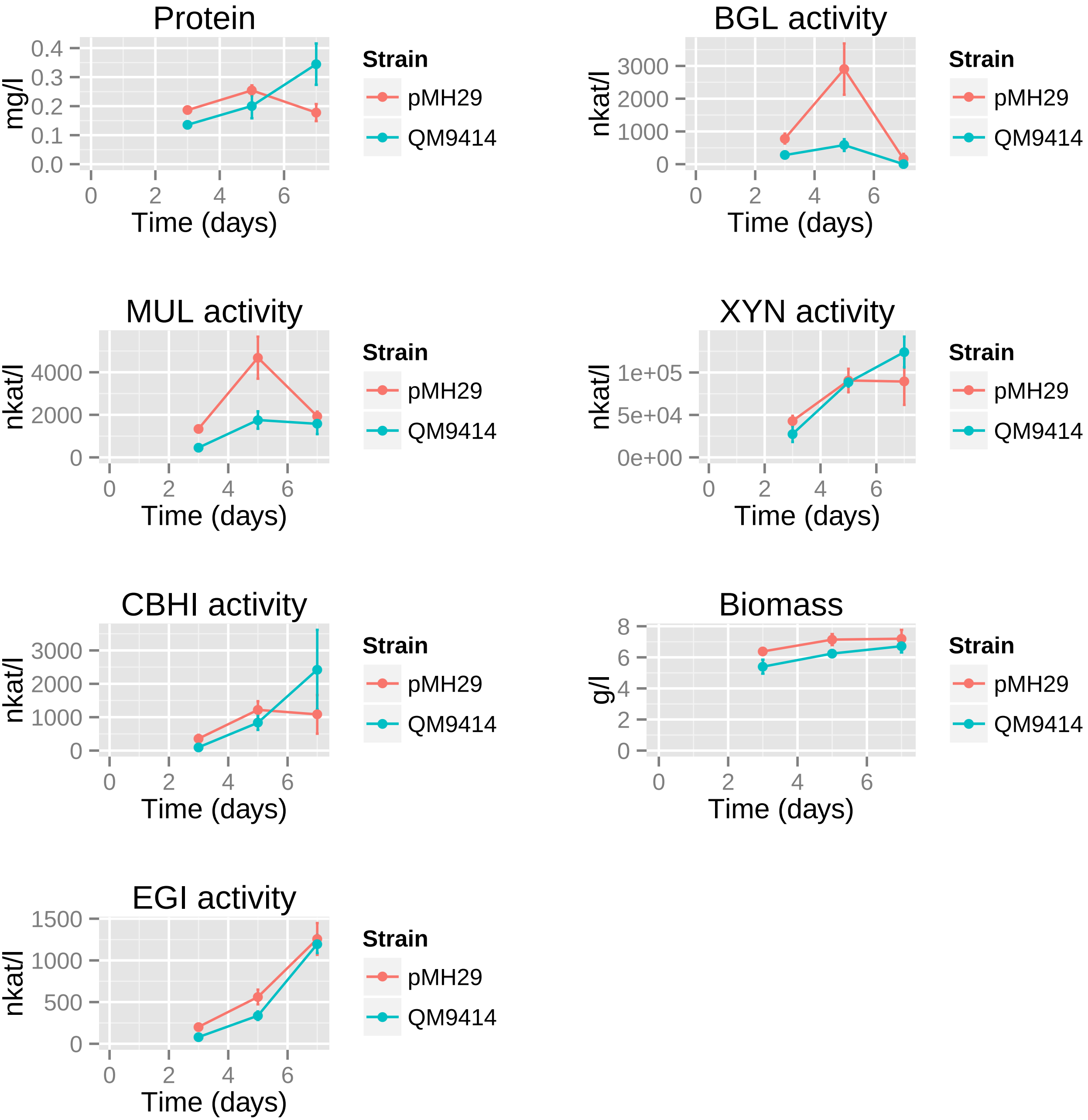
Protein
mg/l
0.4
0.3
0.2
0.1
0.0
0
2
4
6
Time (days)
Strain
pMH29
QM9414
BGL activity
nkat/l
3000
2000
1000
0
0
2
4
6
Time (days)
Strain
pMH29
QM9414
MUL activity
nkat/l
4000
2000
0
0
2
4
6
Time (days)
Strain
pMH29
QM9414
XYN activity
nkat/l
1e+05
5e+04
0e+00
0
2
4
6
Time (days)
Strain
pMH29
QM9414
CBHI activity
nkat/l
3000
2000
1000
0
0
2
4
6
Time (days)
Strain
pMH29
QM9414
Biomass
g/l
8
6
4
2
0
0
2
4
6
Time (days)
Strain
pMH29
QM9414
EGI activity
nkat/l
1500
1000
500
0
0
2
4
6
Time (days)
Strain
pMH29
QM9414

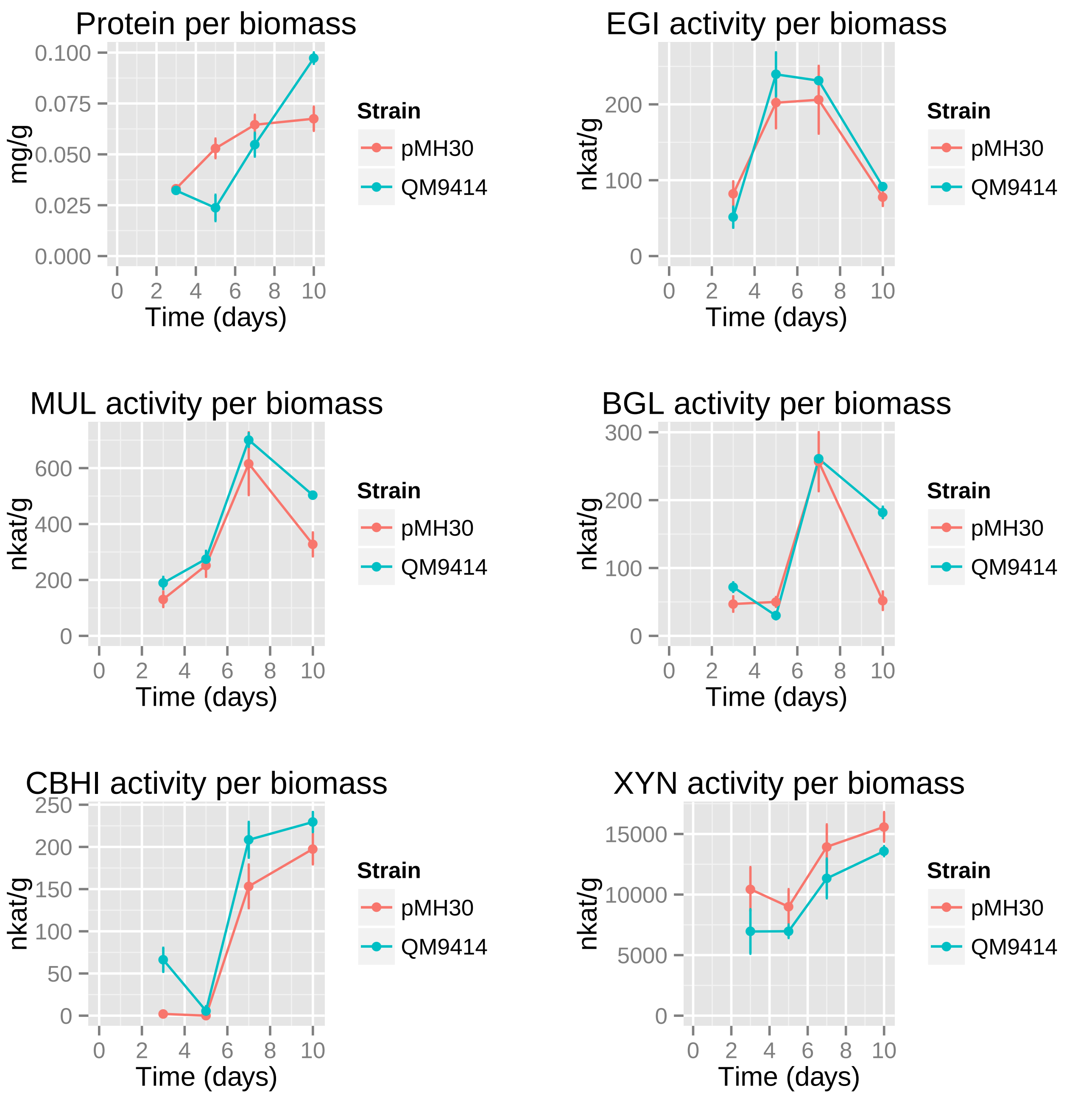

Protein per biomass
mg/g
0.000
0.025
0.050
0.075
0.100
0
2
4
6
8
10
Time (days)
Strain
pMH30
QM9414
EGI activity per biomass
nkat/g
0
100
200
Time (days)
MUL activity per biomass
nkat/g
0
200
400
600
Time (days)
BGL activity per biomass
nkat/g
0
100
200
300
Time (days)
CBHI activity per biomass
nkat/g
0
50
100
150
200
250
Time (days)
XYN activity per biomass
nkat/g
0
5000
10000
15000
Time (days)

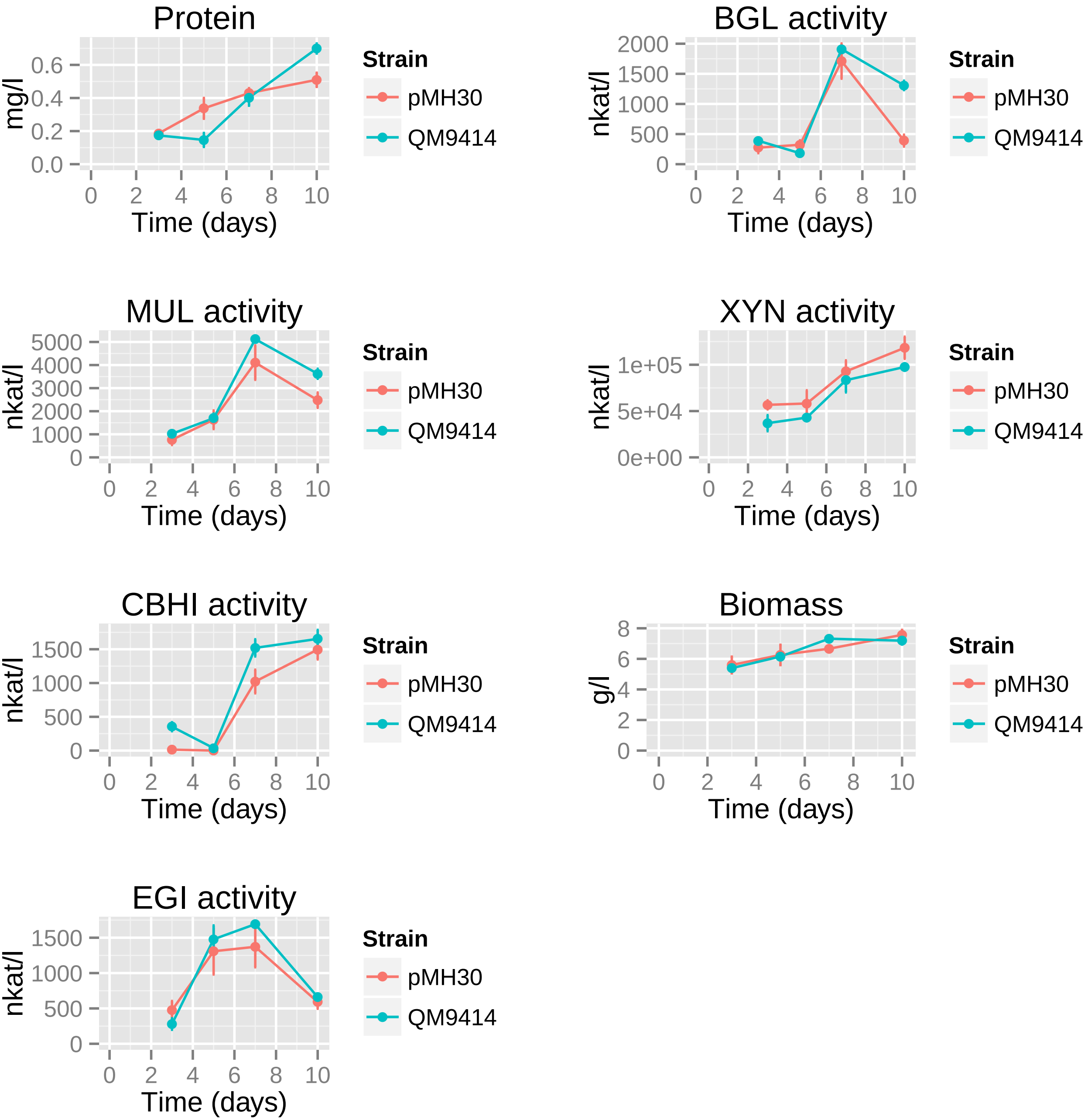
Protein
mg/l
0.0
0.2
0.4
0.6
Time (days)
0
2
4
6
8
10
Strain
pMH30
QM9414
BGL activity
nkat/l
0
500
1000
1500
2000
Time (days)
MUL activity
nkat/l
0
1000
2000
3000
4000
5000
Time (days)
XYN activity
nkat/l
0e+00
5e+04
1e+05
Time (days)
CBHI activity
nkat/l
0
500
1000
1500
Time (days)
Biomass
g/l
0
2
4
6
8
Time (days)
EGI activity
nkat/l
0
500
1000
1500
Time (days)

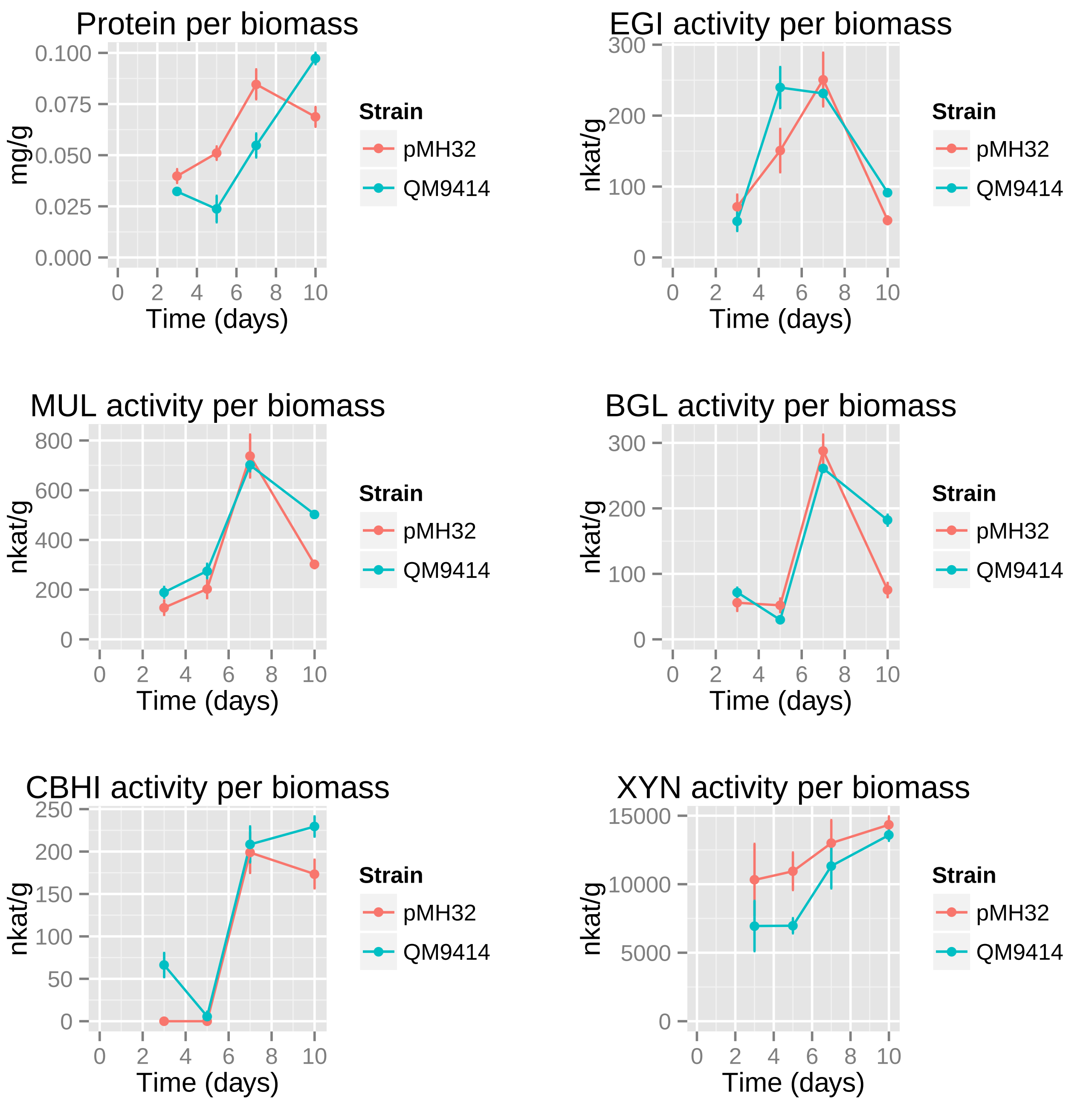
Protein per biomass
mg/g
Time (days)
EGI activity per biomass
MUL activity per biomass
BGL activity per biomass
CBHI activity per biomass
XYN activity per biomass
nkat/g
Strain
pMH32
QM9414

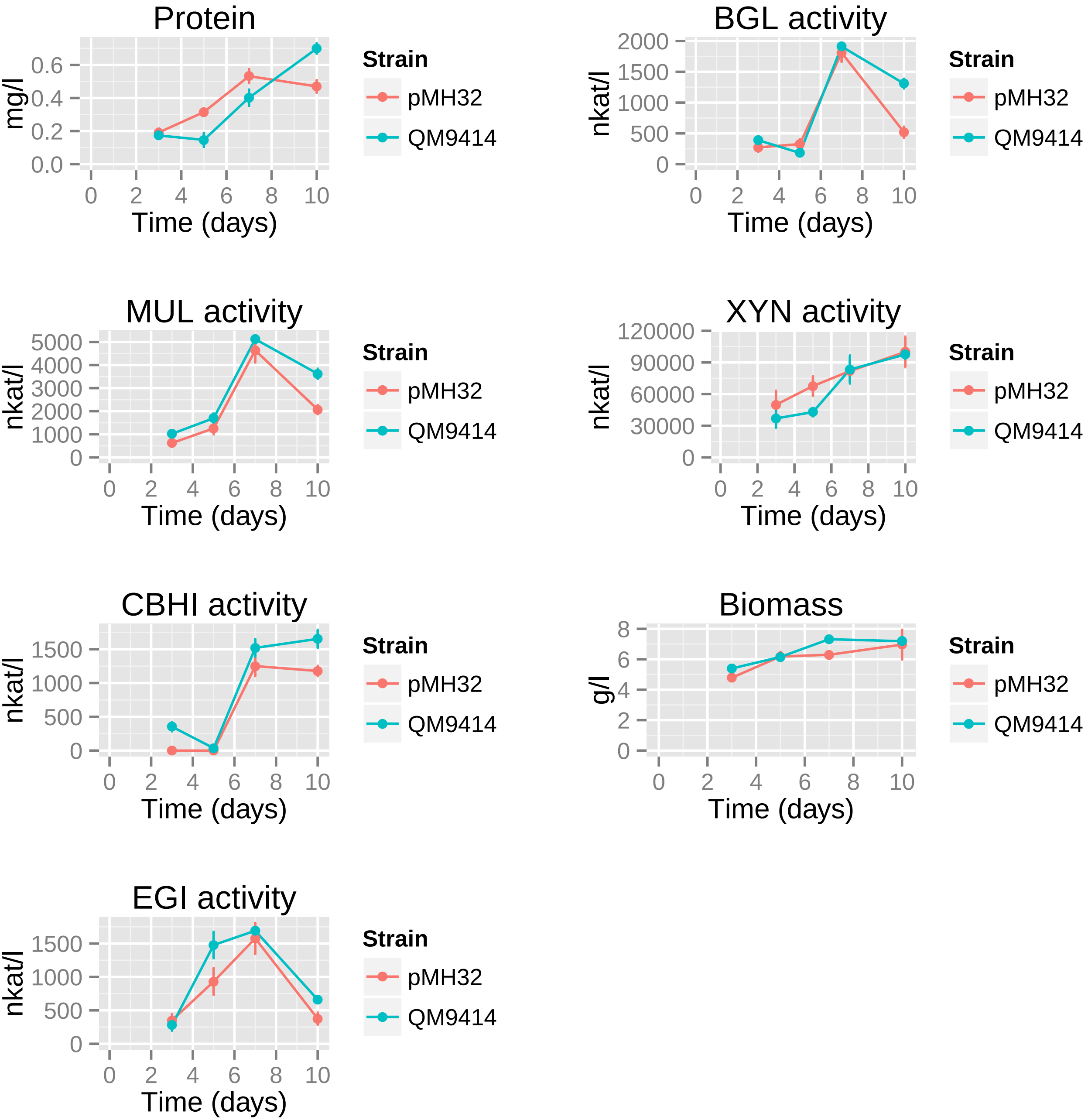

Protein
mg/l
0.0
0.2
0.4
0.6
Time (days)
0
2
4
6
8
10
Strain
pMH32
QM9414
BGL activity
nkat/l
0
500
1000
1500
2000
MUL activity
nkat/l
0
1000
2000
3000
4000
5000
XYN activity
nkat/l
0
30000
60000
90000
120000
CBHI activity
nkat/l
0
500
1000
1500
Biomass
g/l
0
2
4
6
8
EGI activity
nkat/l
0
500
1000
1500

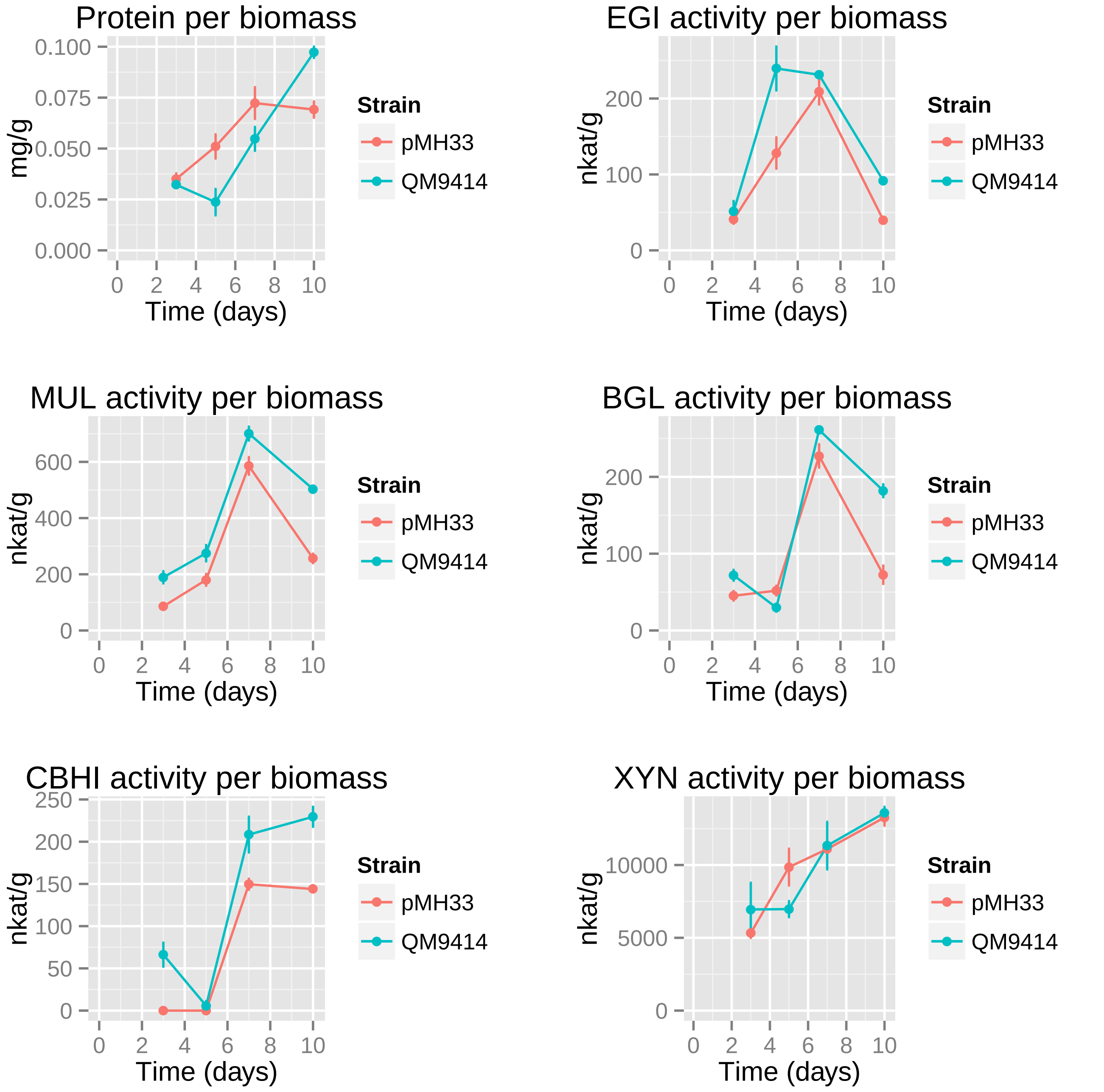
Protein per biomass
mg/g
0.100
0.075
0.050
0.025
0.000
0
2
4
6
8
10
Time (days)
Strain
pMH33
QM9414
EGI activity per biomass
nkat/g
200
100
0
MUL activity per biomass
600
400
BGL activity per biomass
CBHI activity per biomass
250
150
50
XYN activity per biomass
10000
5000

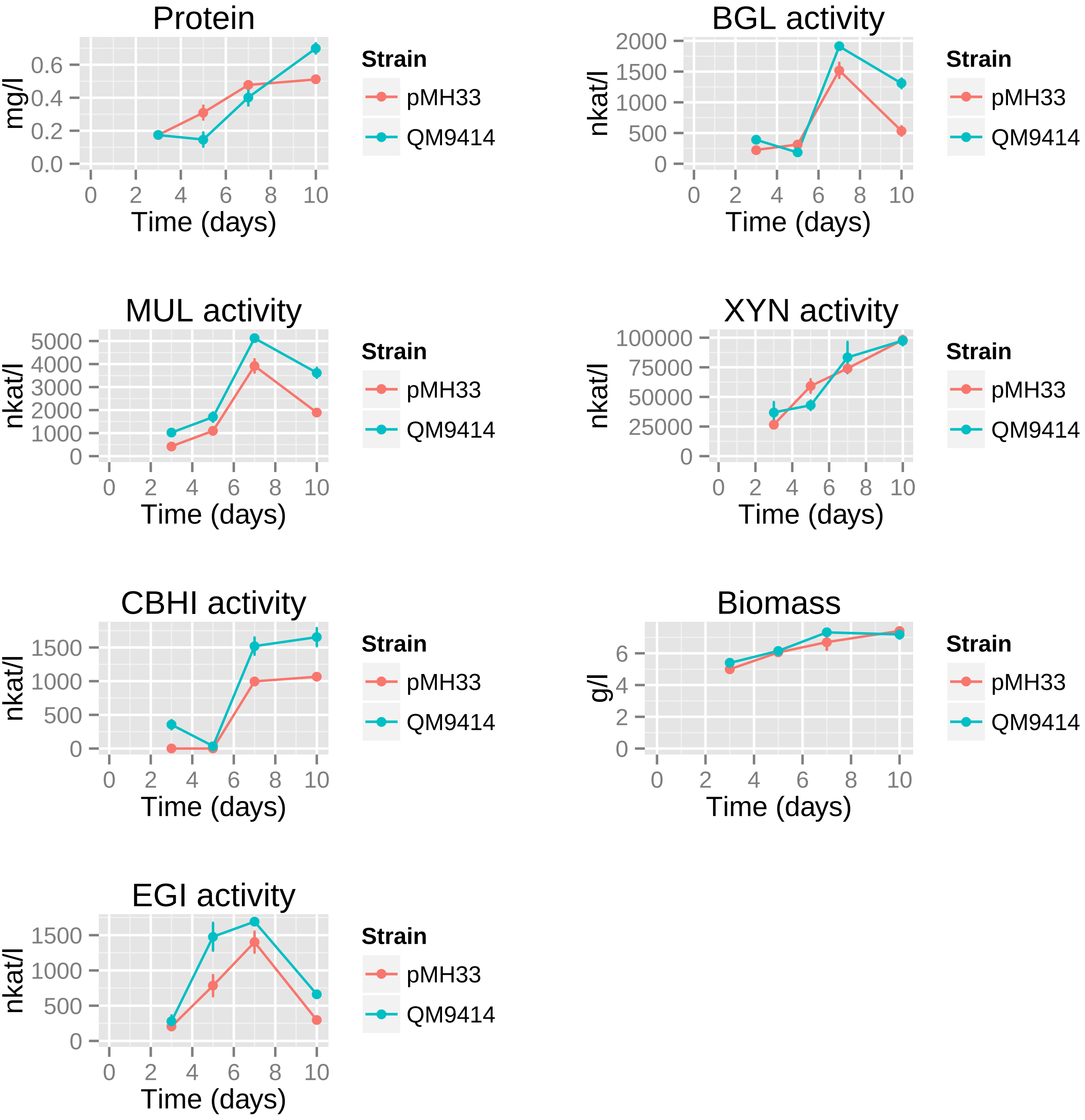
Protein
mg/l
0.0
0.2
0.4
0.6
0
2
4
6
8
10
Time (days)
Strain
pMH33
QM9414
BGL activity
nkat/l
0
500
1000
1500
2000
Time (days)
Strain
pMH33
QM9414
MUL activity
nkat/l
0
1000
2000
3000
4000
5000
Time (days)
Strain
pMH33
QM9414
XYN activity
nkat/l
0
25000
50000
75000
100000
Time (days)
Strain
pMH33
QM9414
CBHI activity
nkat/l
0
500
1000
1500
Time (days)
Strain
pMH33
QM9414
Biomass
g/l
0
2
4
6
Time (days)
Strain
pMH33
QM9414
EGI activity
nkat/l
0
500
1000
1500
Time (days)
Strain
pMH33
QM9414

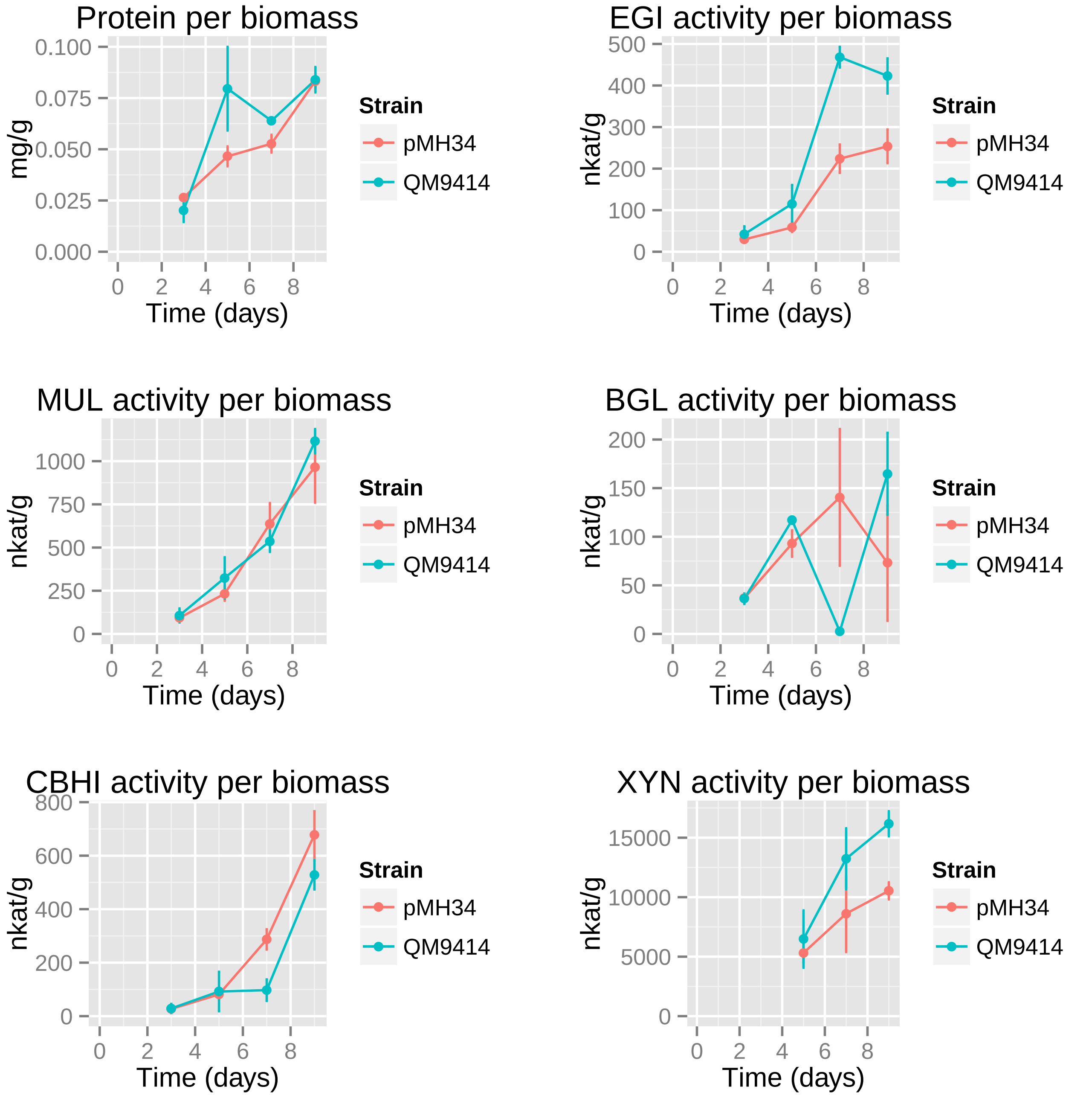
Protein per biomass
mg/g
0.100
0.075
0.050
0.025
0.000
0
2
4
6
8
Time (days)
EGI activity per biomass
nkat/g
500
400
300
200
100
0
Time (days)
MUL activity per biomass
nkat/g
1000
750
500
250
0
Time (days)
BGL activity per biomass
nkat/g
200
150
100
50
0
Time (days)
CBHI activity per biomass
nkat/g
800
600
400
200
0
Time (days)
XYN activity per biomass
nkat/g
15000
10000
5000
0
Time (days)
Strain
pMH34
QM9414

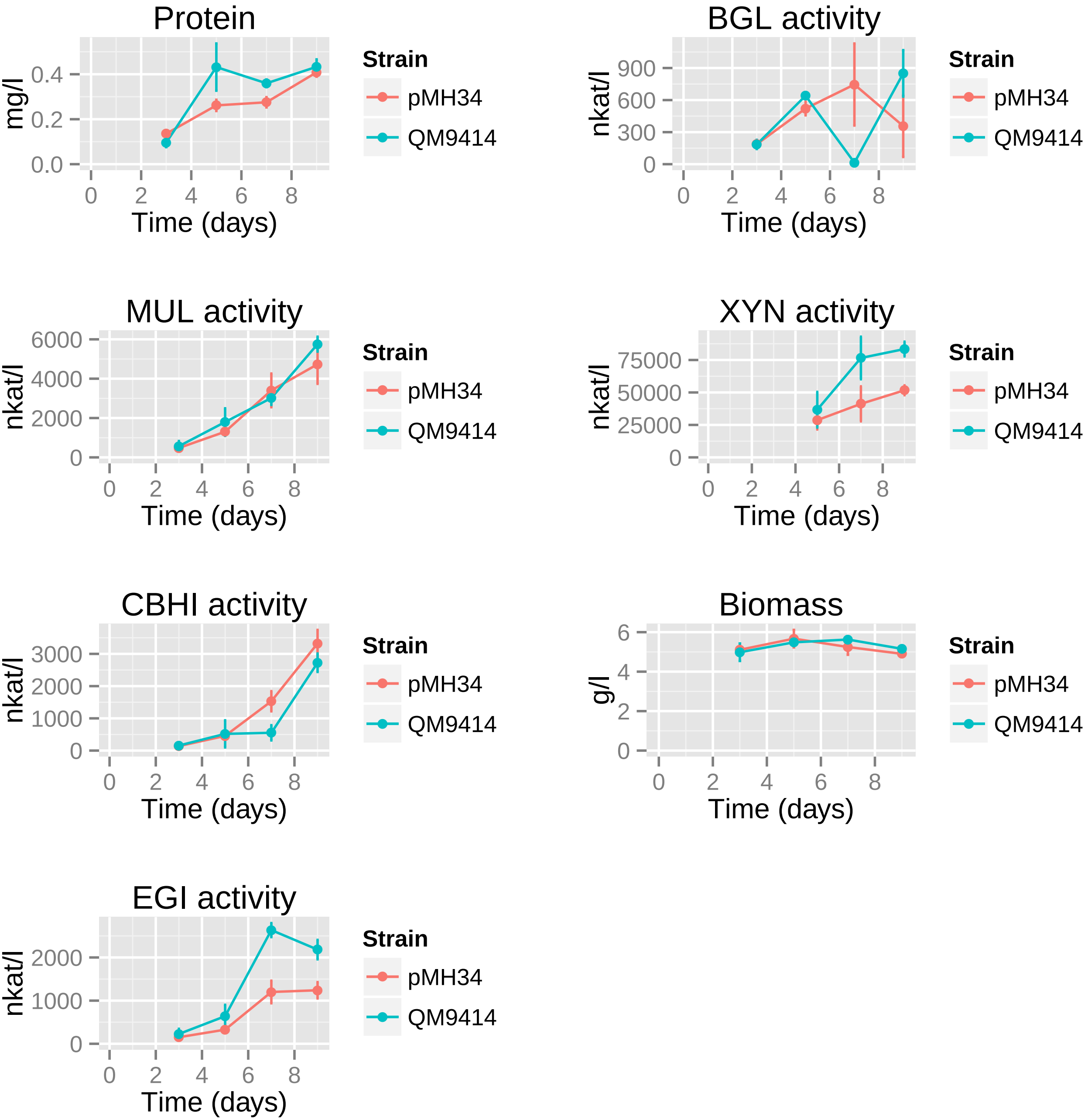

Protein
mg/l
0.0
0.2
0.4
BGL activity
nkat/l
0
300
600
900
MUL activity
nkat/l
0
2000
4000
6000
XYN activity
nkat/l
0
25000
50000
75000
CBHI activity
nkat/l
0
1000
2000
3000
Biomass
g/l
0
2
4
6
EGI activity
nkat/l
0
1000
2000
Time (days)
0
2
4
6
8
Strain
pMH34
QM9414

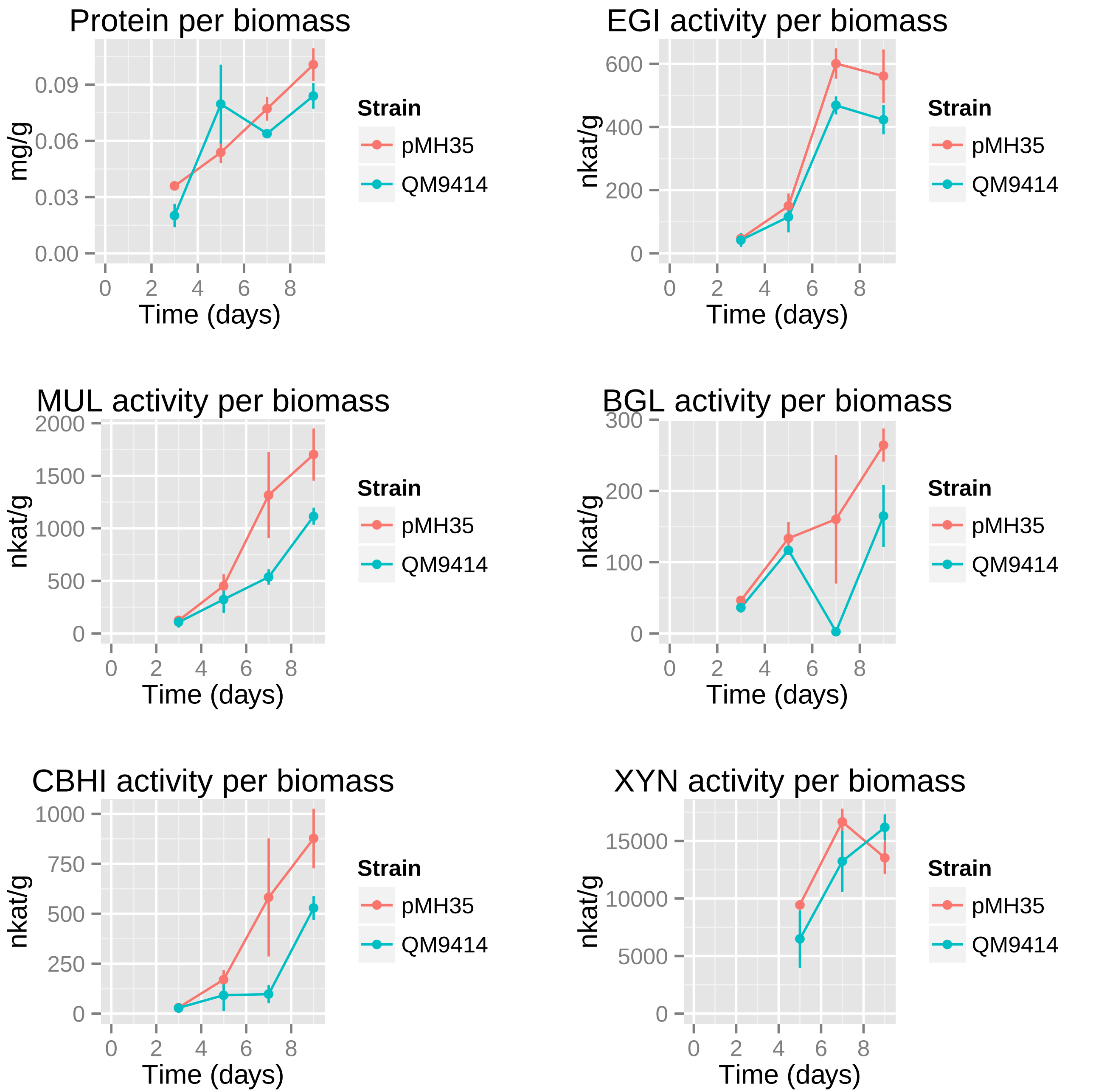
Protein per biomass
mg/g
0.00
0.03
0.06
0.09
0
2
4
6
8
Time (days)
Strain
pMH35
QM9414
EGI activity per biomass
nkat/g
0
200
400
600
0
2
4
6
8
Time (days)
Strain
pMH35
QM9414
MUL activity per biomass
nkat/g
0
500
1000
1500
2000
0
2
4
6
8
Time (days)
Strain
pMH35
QM9414
BGL activity per biomass
nkat/g
0
100
200
300
0
2
4
6
8
Time (days)
Strain
pMH35
QM9414
CBHI activity per biomass
nkat/g
0
250
500
750
1000
0
2
4
6
8
Time (days)
Strain
pMH35
QM9414
XYN activity per biomass
nkat/g
0
5000
10000
15000
0
2
4
6
8
Time (days)
Strain
pMH35
QM9414

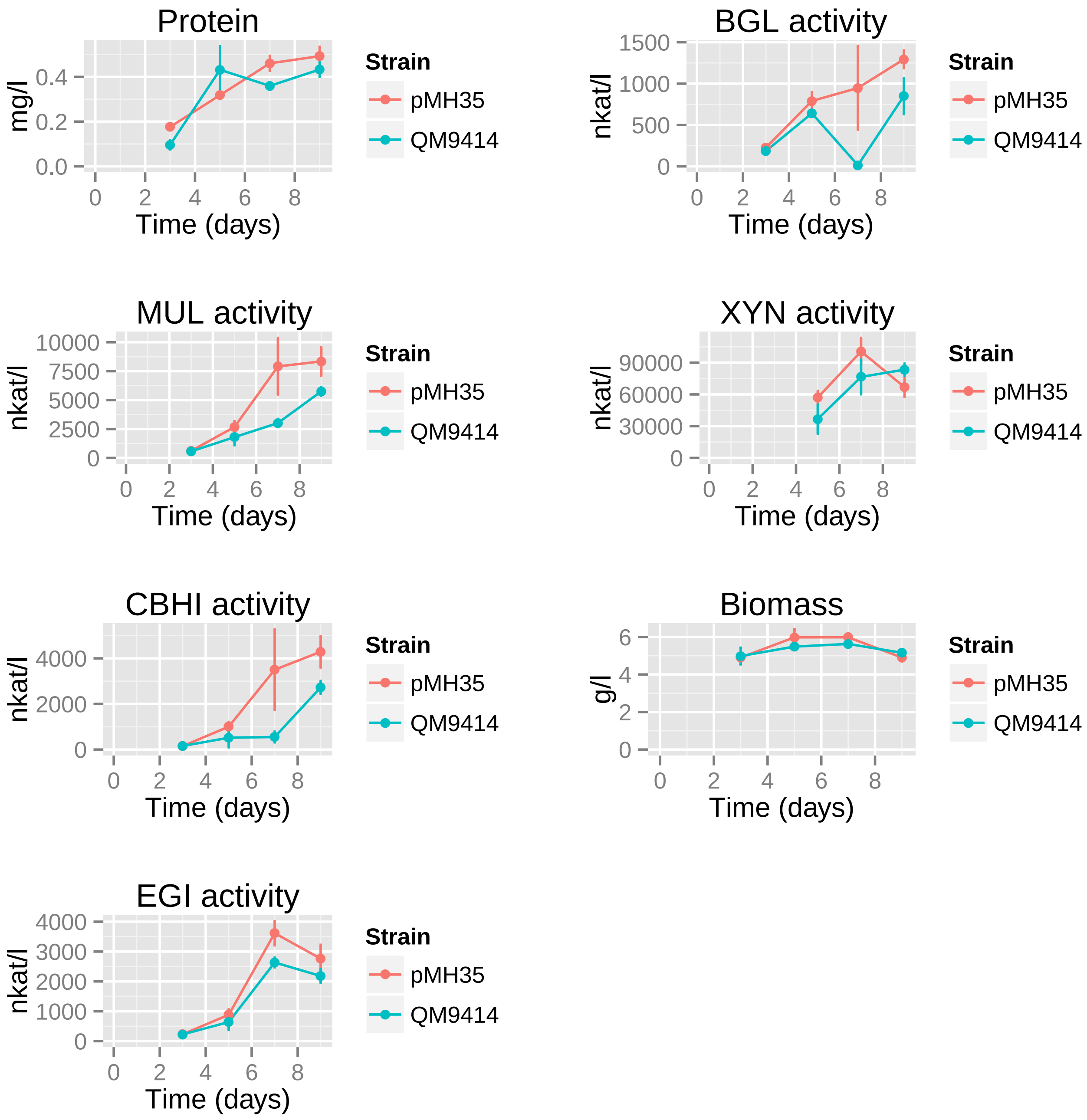
Protein
mg/l
0.0
0.2
0.4
BGL activity
MUL activity
XYN activity
CBHI activity
Biomass
g/l
EGI activity
nkat/l
Time (days)
0
2
4
6
8
Strain
pMH35
QM9414

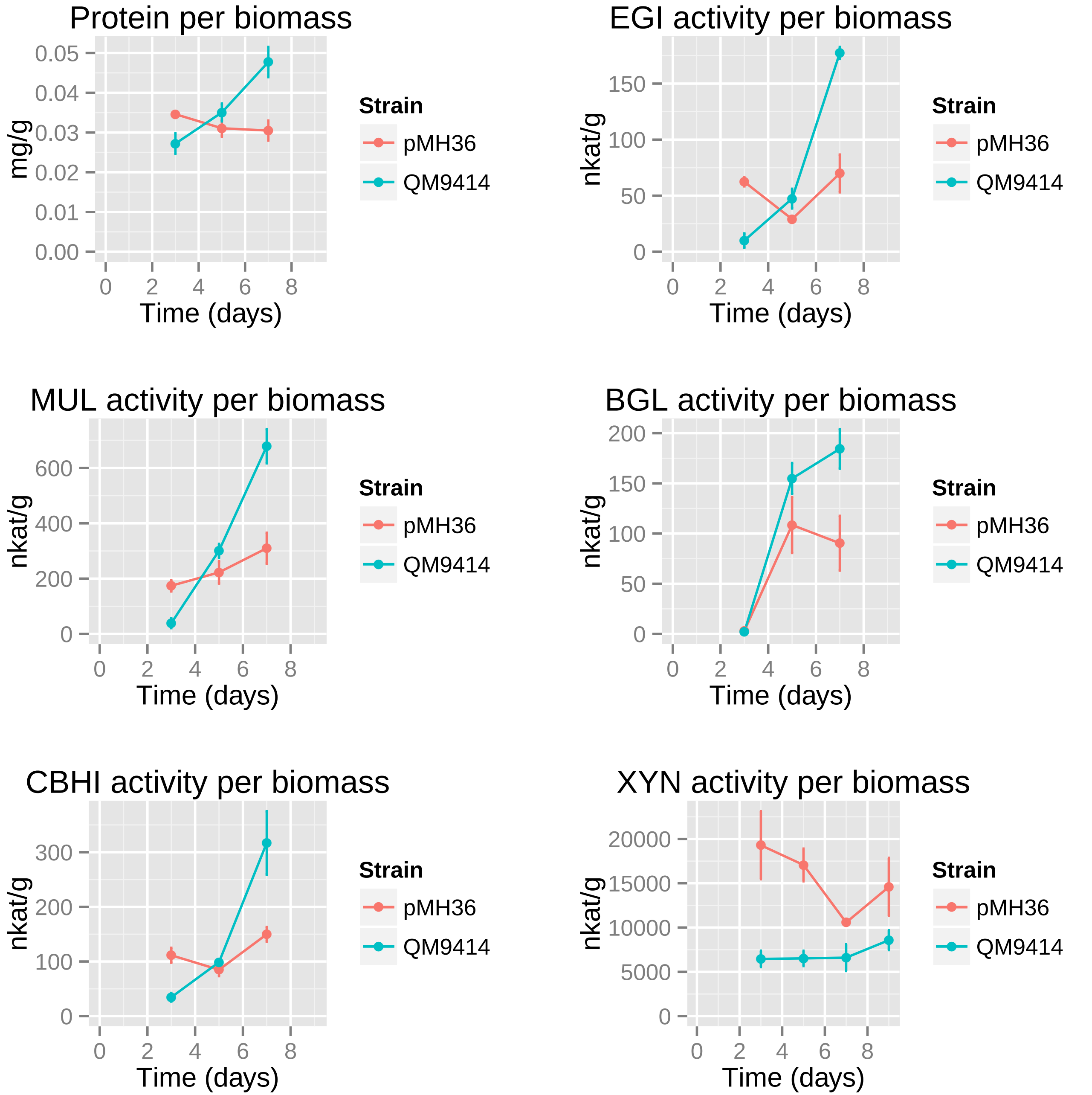

Protein per biomass
mg/g
0.05
0.04
0.03
0.02
0.01
0.00
0
2
4
6
8
Time (days)
Strain
pMH36
QM9414
EGI activity per biomass
nkat/g
150
100
50
0
Time (days)
MUL activity per biomass
nkat/g
600
400
200
0
Time (days)
BGL activity per biomass
nkat/g
200
150
100
50
0
Time (days)
CBHI activity per biomass
nkat/g
300
200
100
0
Time (days)
XYN activity per biomass
nkat/g
20000
15000
10000
5000
0
Time (days)

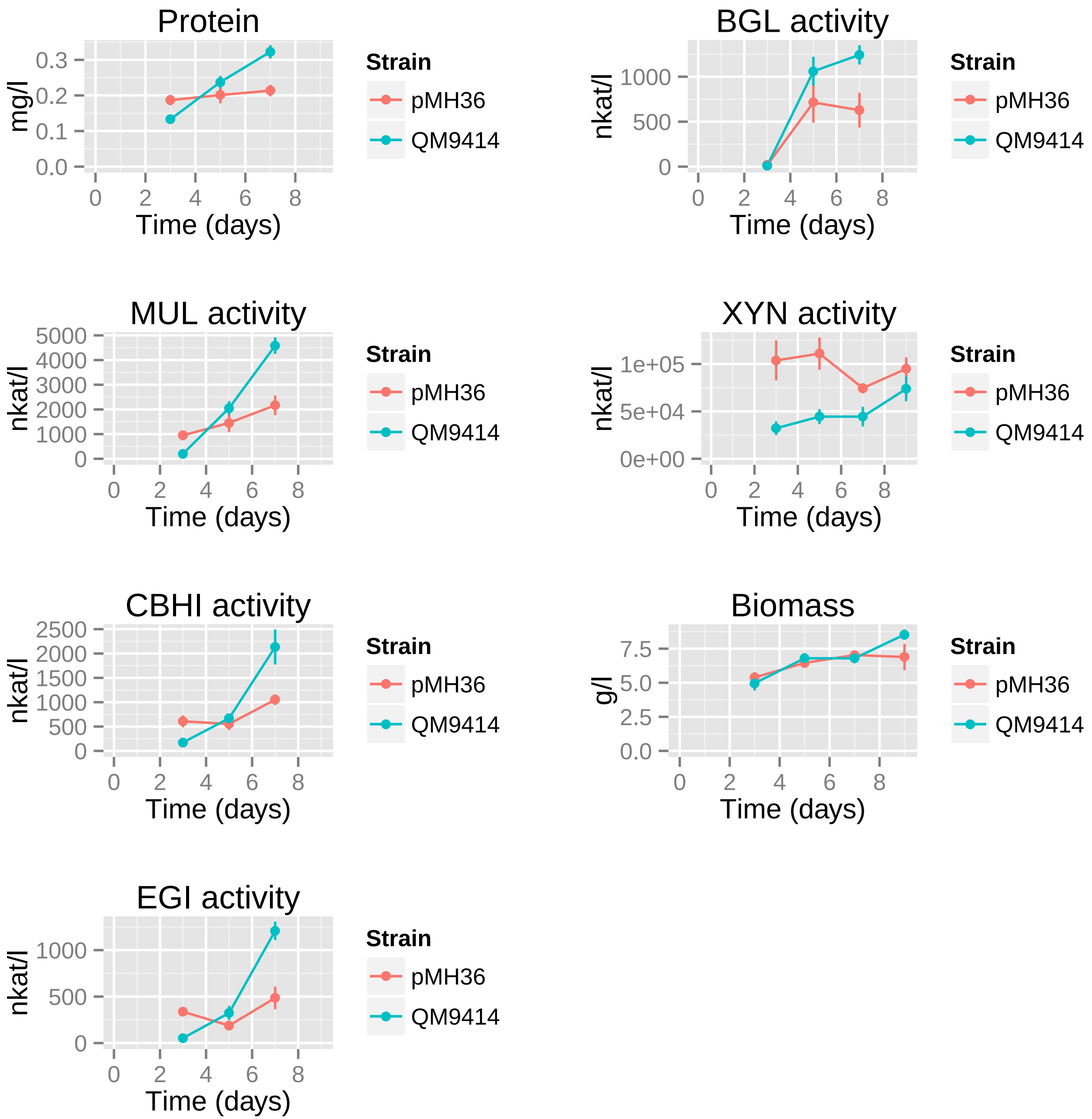
Protein
mg/l
0.0
0.1
0.2
0.3
Time (days)
0
2
4
6
8
Strain
pMH36
QM9414
BGL activity
nkat/l
0
500
1000
MUL activity
nkat/l
0
1000
2000
3000
4000
5000
XYN activity
nkat/l
0e+00
5e+04
1e+05
CBHI activity
nkat/l
0
500
1000
1500
2000
2500
Biomass
g/l
0.0
2.5
5.0
7.5
EGI activity
nkat/l
0
500
1000

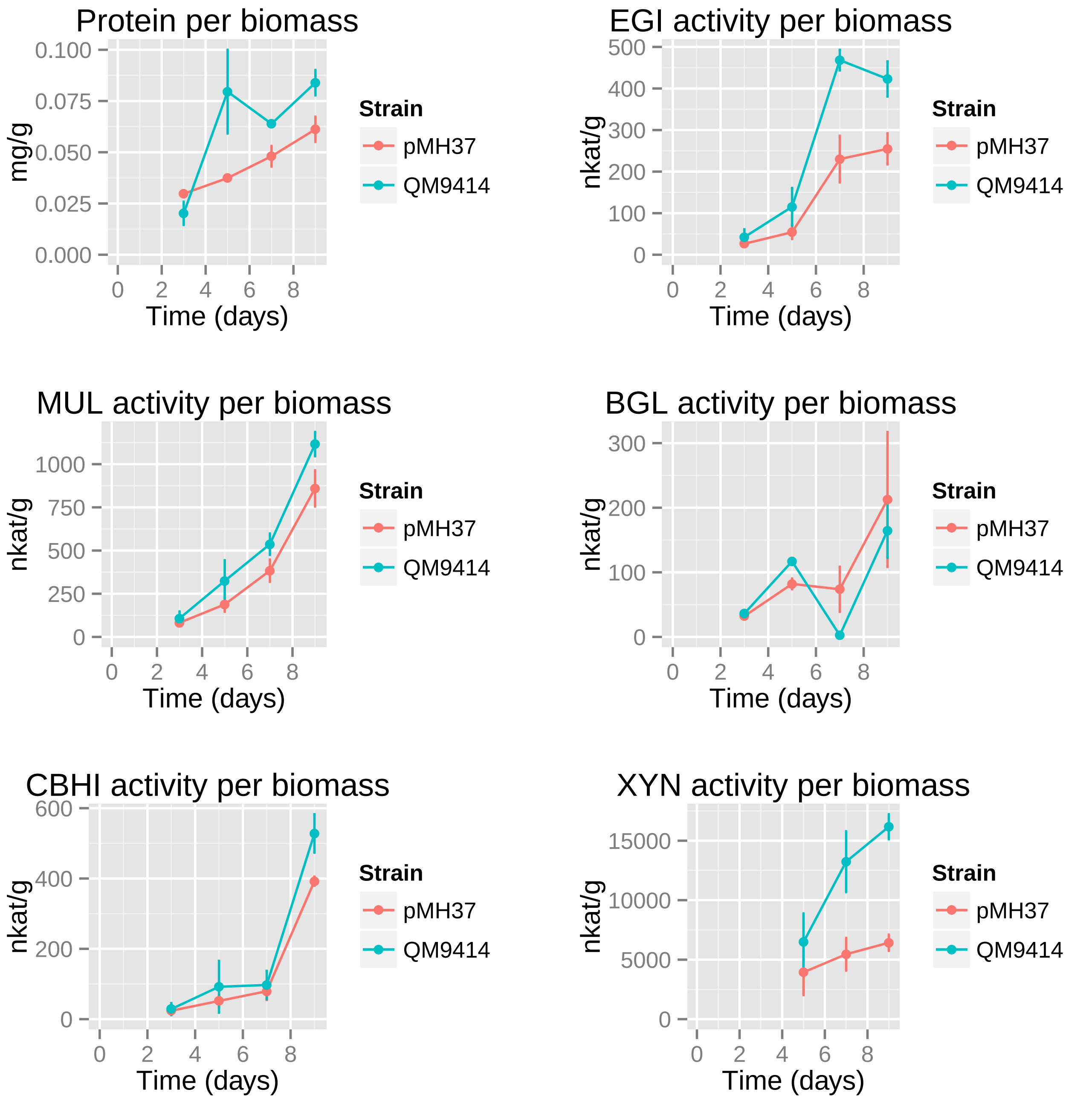

Protein per biomass
mg/g
0.100
0.075
0.050
0.025
0.000
0
2
4
6
8
Time (days)
Strain
pMH37
QM9414
EGI activity per biomass
nkat/g
500
400
300
200
100
0
Time (days)
MUL activity per biomass
nkat/g
1000
750
500
250
0
Time (days)
BGL activity per biomass
nkat/g
300
200
100
0
Time (days)
CBHI activity per biomass
nkat/g
600
400
200
0
Time (days)
XYN activity per biomass
nkat/g
15000
10000
5000
0
Time (days)

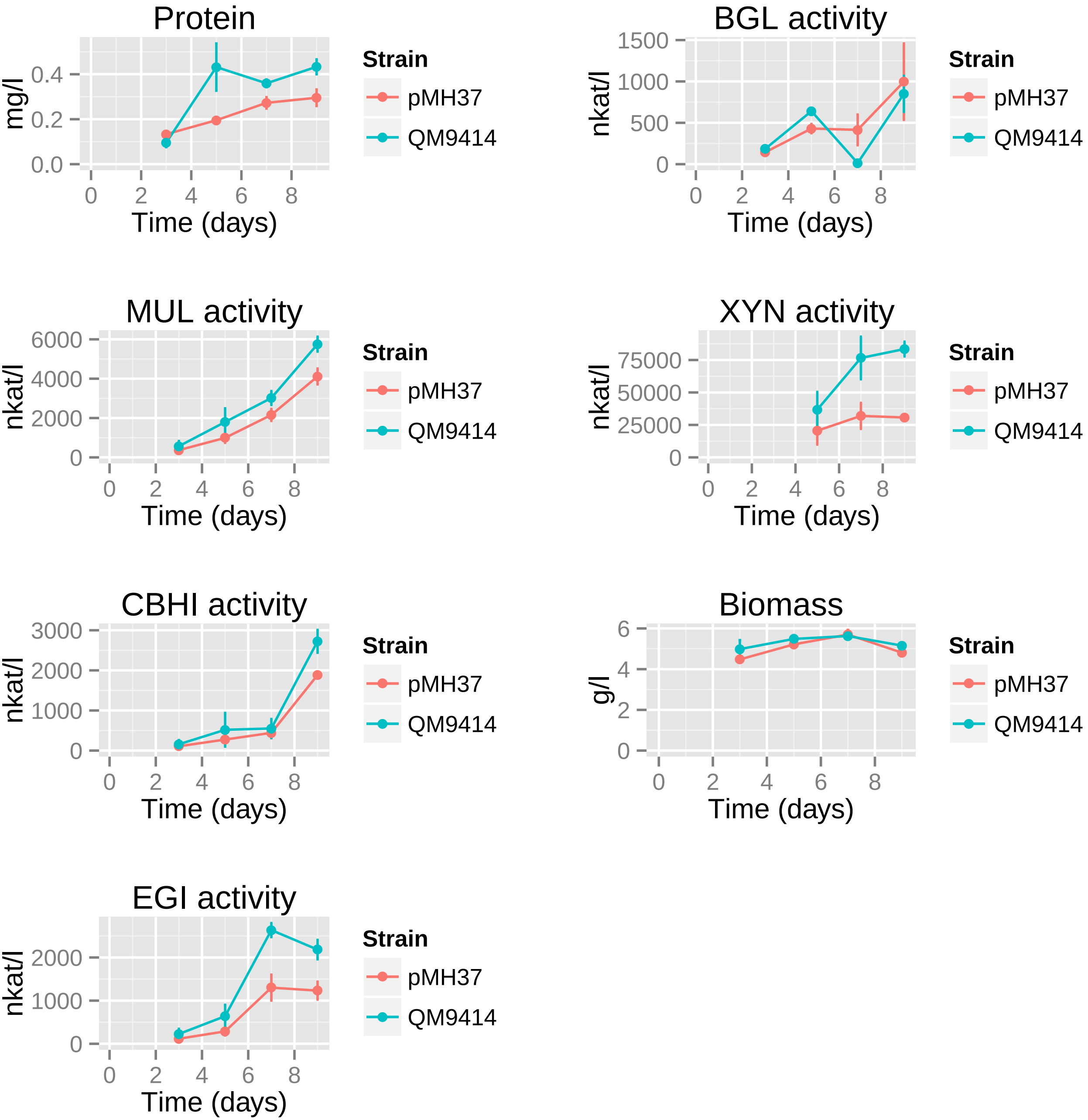
Protein
mg/l
0.0
0.2
0.4
0
2
4
6
8
Time (days)
Strain
pMH37
QM9414
BGL activity
nkat/l
0
500
1000
1500
0
2
4
6
8
Time (days)
Strain
pMH37
QM9414
MUL activity
nkat/l
0
2000
4000
6000
0
2
4
6
8
Time (days)
Strain
pMH37
QM9414
XYN activity
nkat/l
0
25000
50000
75000
0
2
4
6
8
Time (days)
Strain
pMH37
QM9414
CBHI activity
nkat/l
0
1000
2000
3000
0
2
4
6
8
Time (days)
Strain
pMH37
QM9414
Biomass
g/l
0
2
4
6
0
2
4
6
8
Time (days)
Strain
pMH37
QM9414
EGI activity
nkat/l
0
1000
2000
0
2
4
6
8
Time (days)
Strain
pMH37
QM9414
