# Supplementary material for: Screening of candidate regulators for cellulase and hemicellulase production in Trichoderma reesei and identification of a factor essential for cellulase production
Source: Biotechnol Biofuels. 2014 Jan 28;7:14. doi: 10.1186/1754-6834-7-14 (PMC3922861; doi:10.1186/1754-6834-7-14)
Supplement: Additional file 3 — Results of Southern hybridizations. Position of the molecular weight size marker is shown as kb on the left. The restriction enzymes used for the digestion in the analysis are indicated by the letters: A, NcoI + BstXI; B, BglII; C, SpeI + BclI; D, ClaI + BamHI; E, SacI; F, NaeI; G, ClaI + XbaI; H, SnaBI + XbaI; I, StuI; J, SacI; K, StuI; L, XmnI; M, BstEII; N, SspI; O, StuI; P, SspI; Q, StuI. For Del77513 strain two different probes were used: hygromycin selection marker (hph) open reading frame (N and O) and fragment of the gene 77513 open reading frame (P and Q). [file 1754-6834-7-14-S3.pdf]

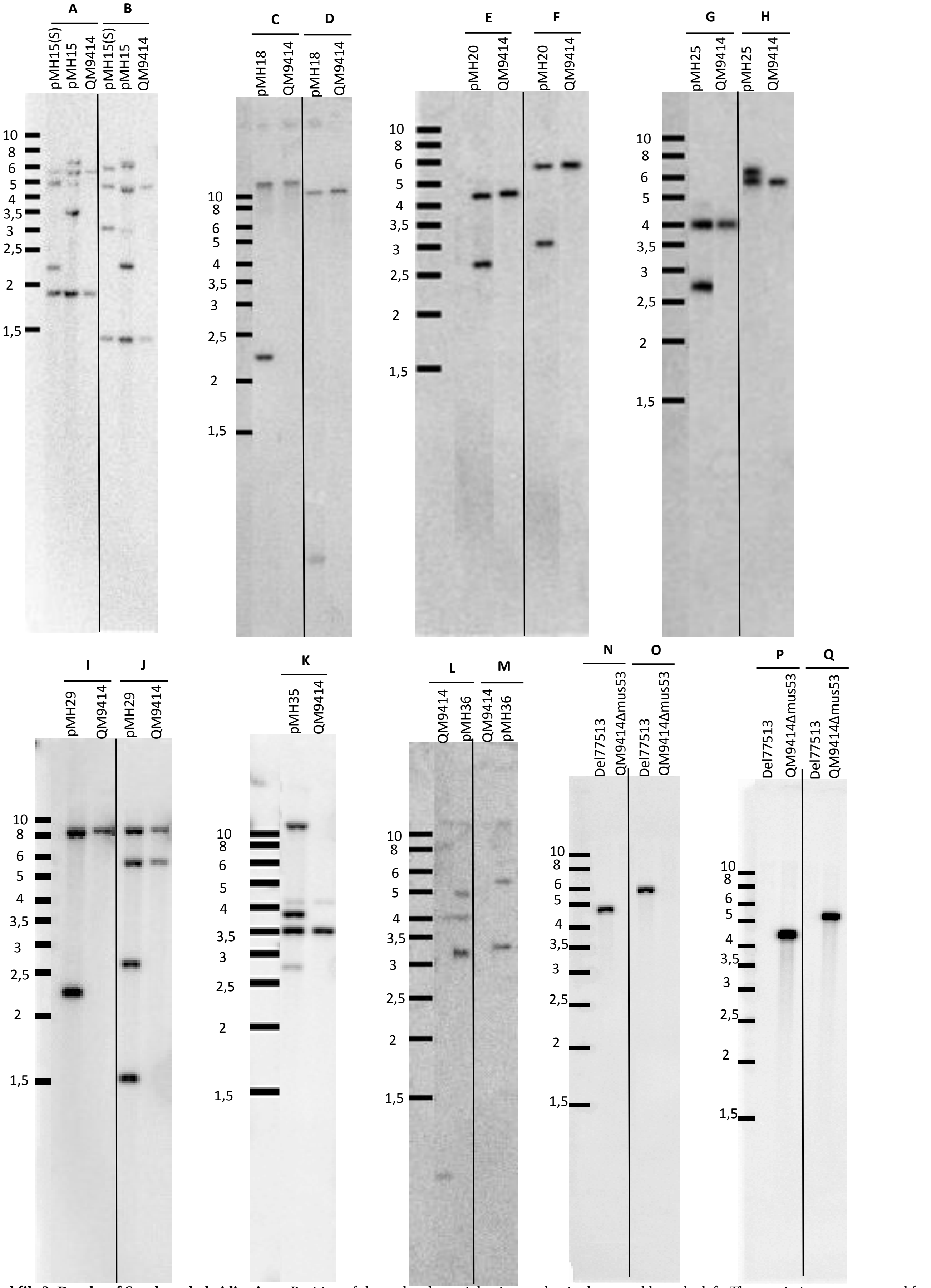

Additional file 3. Results of Southern hybridisations. Position of the molecular weight size marker is shown as kb on the left. The restriction enzymes used for the digestion in the analysis are indicated by the letters: A, *NcoI+BstXI*; B, *BglII*; C, *SpeI+BclI*; D, *ClaI+BamHI*; E, *SacI*; F, *NaeI*; G, *ClaI+XbaI*; H, *SnaBI+XbaI*; I, *StuI*; J, *SacI*; K, *StuI*; L, *XmnI*; M, *BstEII*; N, *SspI*; O, *StuI*; P, *SspI*; Q, *StuI*. For Del77513 strain two different probes were used: hygromycin selection marker (hph) open reading frame (N and O) and fragment of the gene 77513 open reading frame (P and Q).
