# Supplementary material for: Screening of candidate regulators for cellulase and hemicellulase production in Trichoderma reesei and identification of a factor essential for cellulase production
Source: Biotechnol Biofuels. 2014 Jan 28;7:14. doi: 10.1186/1754-6834-7-14 (PMC3922861; doi:10.1186/1754-6834-7-14)
Supplement: Additional file 4 — Results of Northern hybridizations. Northern blot analysis of the expression of the candidate regulatory genes in the recombinant strains. (A) mRNA signals of genes 123668, 80291, 74765, 122523, 66966 and 64608 in cultures of the strains harboring the corresponding overexpression cassettes pMH18, pMH20, pMH25, pMH29, pMH35 and pMH36, respectively, are shown on the top. The mobility of the transcript encoded by the overexpression construct is indicated by an arrow in the blot. Samples collected after 3 days of cultivation (two biological replicates) were analyzed. The northern hybridization signal of actin and staining of total RNA with the SYBR Green II in the same gels are shown below each of the northern blots, as indicated. (B) mRNA signals of gene 77513 in cultures of overexpression strains pMH15 and pMH15(S), and in the Del77513 strain. Samples collected after 3 and 5 days of cultivation (two biological replicates) were analyzed. The northern hybridization signal of actin and staining of total RNA with the SYBR Green II in the same gel are shown below, as indicated. (C) Signal fold change of the northern signals in the recombinant strain versus the control strain. Signal intensities were normalized using the actin signal. [file 1754-6834-7-14-S4.pdf]

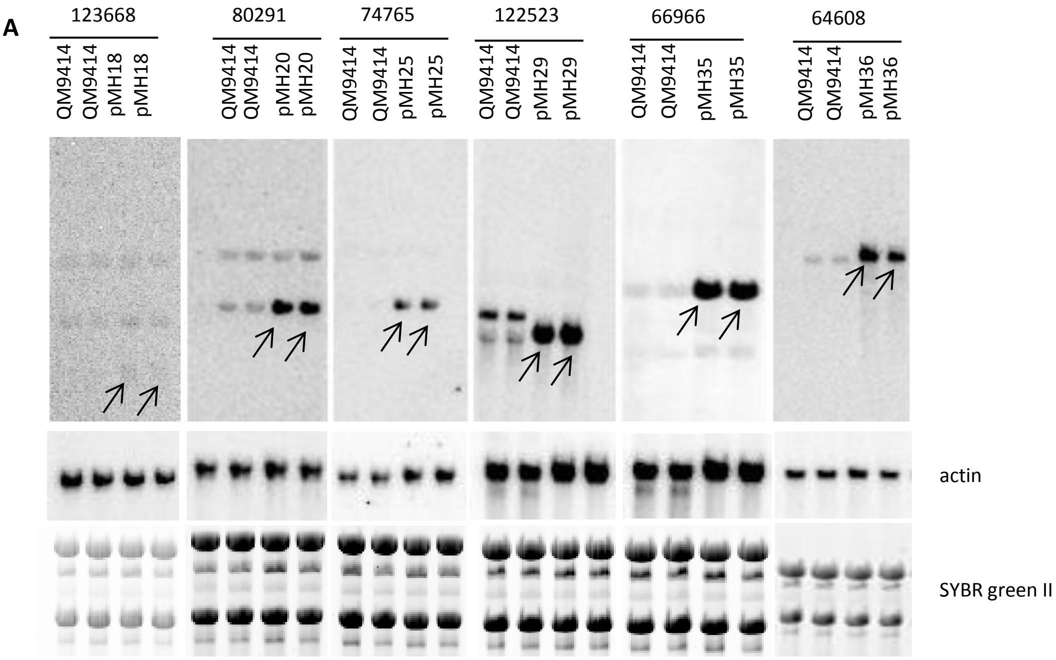

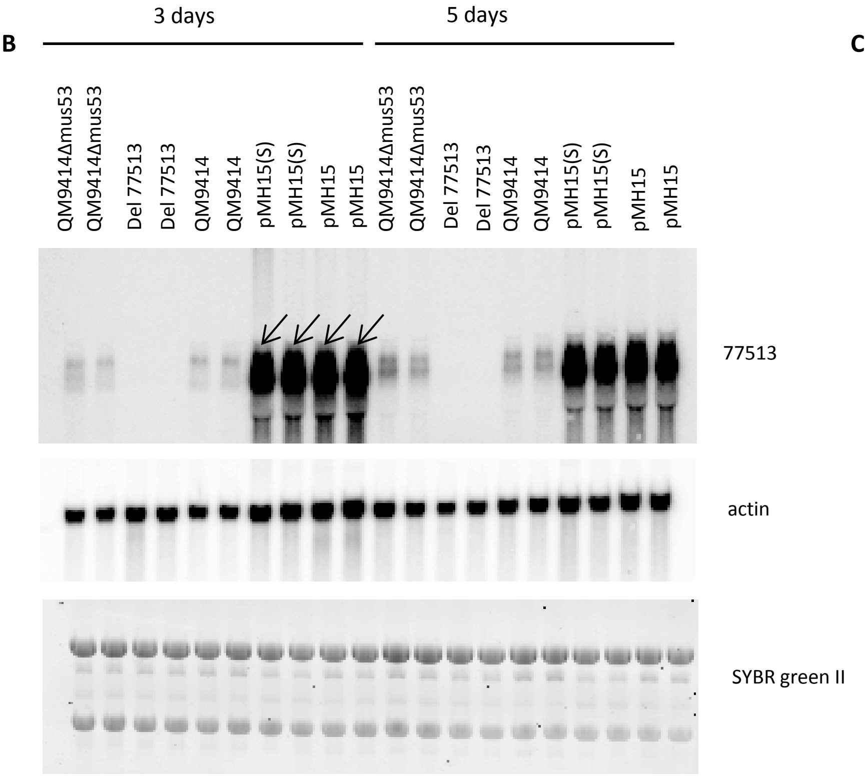

C

	Fold change vs. parental strain, 3 days	Fold change vs. parental strain, 5 days
pMH20	3.5	-
pMH25	17.4	-
pMH29	1.4	-
pMH35	8.4	-
pMH36	12.9	-
pMH15	23.6	10.6
pMH15(S)	21.9	13.9
Del77513	-0.2	-0.01

Additional file 4. Results of Northern hybridisations. Northern blot analysis of the expression of the candidate regulatory genes in the recombinant strains. A. mRNA signals of the genes 123668, 80291, 74765, 122523, 66966 and 64608 in cultures of the strains harbouring the corresponding over-expression cassettes pMH18, pMH20, pMH25, pMH29, pMH35 and pMH36, respectively, are shown on the top. The mobility of the transcript encoded by the overexpression construct is indicated by an arrow in the blot. Samples collected after 3 days of cultivation (two biological replicates) were analysed. The Northern hybridisation signal of actin and staining of total RNA with the SYBR Green II in the same gels are shown below each of the Northern blot, as indicated. B. mRNA signals of the gene 77513 in cultures of overexpression strains pMH15 and pMH15(S), and in the Del77513 strain. Samples collected after 3 and 5 days of cultivation (two biological replicates) were analysed. The Northern hybridisation signal of actin and staining of total RNA with the SYBR Green II in the same gel are shown below, as indicated. C. Signal fold change of the Northern signals in the recombinant strain vs. the control strain. Signal intensities were normalised using the actin signal.
