# Supplementary material for: Screening of candidate regulators for cellulase and hemicellulase production in Trichoderma reesei and identification of a factor essential for cellulase production
Source: Biotechnol Biofuels. 2014 Jan 28;7:14. doi: 10.1186/1754-6834-7-14 (PMC3922861; doi:10.1186/1754-6834-7-14)
Supplement: Additional file 5 — pMS204 vector with hygromycin resistance gene and gateway cloning cassette under gpdA promoter and trpC terminator. AmpR, ampicillin resistance gene; attR1/attR2, att sites for recombination; ccdB, ccdB gene for negative selection; CmR, chloramphenicol resistance gene; hph, hygromycin resistance gene; MCS, multiple cloning site; ORI, origin of replication. [file 1754-6834-7-14-S5.pdf]

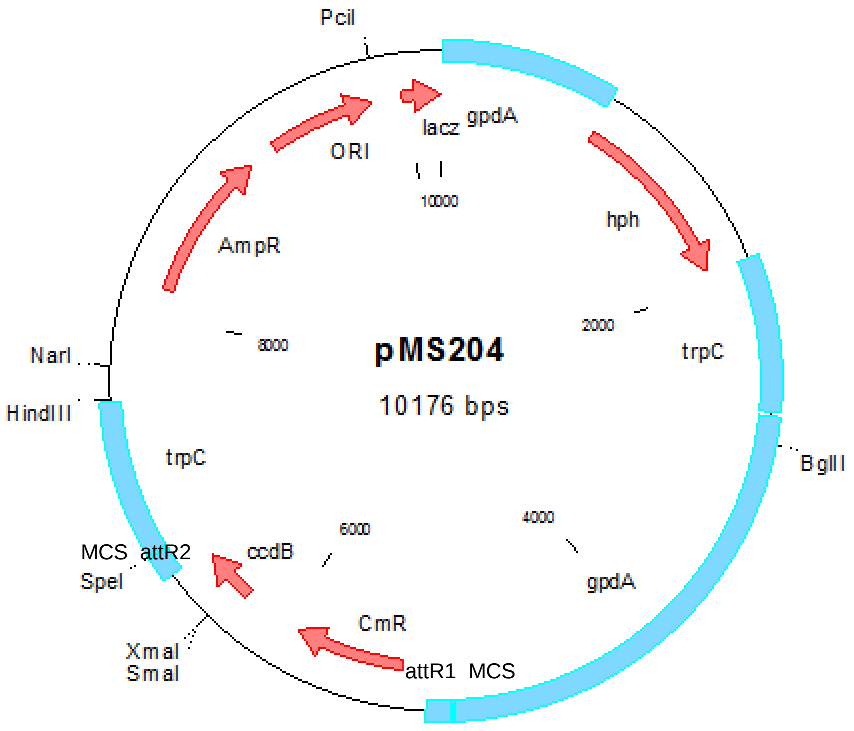

Additional file 5. pMS204 vector with hygromycin resistance gene and gateway cloning cassette under gpdA promoter and trpC terminator. AmpR, ampicillin resistance gene; ORI, origin of replication; hph, hygromycin resistance gene; MCS, multiple cloning site; attR1/attR2, att sites for recombination; ccdB, ccdB gene for negative selection; CmR, Chloramphenicol resistance gene.
